# Late effects of cancer in children, teenagers and young adults: Population-based study on the burden of 183 conditions, in-patient and critical care admissions and years of life lost

**DOI:** 10.1016/j.lanepe.2021.100248

**Published:** 2021-11-14

**Authors:** Wai Hoong Chang, Michail Katsoulis, Yen Yi Tan, Stefanie H. Mueller, Katherine Green, Alvina G. Lai

**Affiliations:** a Institute of Health Informatics, University College London, London, United Kingdom of Great Britain and Northern Ireland; b Great Ormond Street Hospital, London, United Kingdom of Great Britain and Northern Ireland; c Institute of Child Health, University College London, London, United Kingdom of Great Britain and Northern Ireland

**Keywords:** Cancer late effects, Children, teenagers and young adults, Cancer treatment, Primary care, Hospitalisation, Years of life lost

## Abstract

**Background:**

Children, teenagers and young adults who survived cancer are prone to developing late effects. The burden of late effects across a large number of conditions, in-patient hospitalisation and critical care admissions have not been described using a population-based dataset. We aim to systematically quantify the cumulative burden of late effects across all cancer subtypes, treatment modalities and chemotherapy drug classes.

**Methods:**

We employed primary care records linked to hospitals, the death registry and cancer registry from 1998–2020. CTYA survivors were 25 years or younger at the time of cancer diagnosis had survived ≥5 years post-diagnosis. Year-of-birth and sex-matched community controls were used for comparison. We considered nine treatment types, nine chemotherapy classes and 183 physical and mental health late effects. Cumulative burden was estimated using mean cumulative count, which considers recurring events. Multivariable logistic regression was used to investigate the association between treatment exposures and late effects. Excess years of life lost (YLL) attributable to late effects were estimated.

**Findings:**

Among 4,063 patients diagnosed with cancer, 3,466 survived ≥ 5 years (85%); 13,517 matched controls were identified. The cumulative burden of late effects at age 35 was the highest in survivors of leukaemia (23.52 per individual [95% CI:19.85–29.33]) and lowest in survivors of germ cell tumours (CI:6.04 [5.32–6.91]). In controls, the cumulative burden was 3.99 (CI:3.93–4.08) at age 35 years. When survivors reach age 45, the cumulative burden for immunological conditions and infections was the highest (3.27 [CI:3.01–3.58]), followed by cardiovascular conditions (3.08 [CI:1.98–3.29]). Survivors who received chemotherapy and radiotherapy had the highest disease burden compared to those who received surgery only. These patients also had the highest burden of hospitalisation (by age 45: 10.43 [CI:8.27–11.95]). Survivors who received antimetabolite chemotherapy had the highest disease and hospitalisation burden, while the lowest burden is observed in those receiving antitumour antibiotics. Regression analyses revealed that survivors who received only surgery had lower odds of developing cardiovascular (adjusted odds ratio 0.73 [CI:0.56–0.94]), haematological (aOR 0.51 [CI:0.37–0.70]), immunology and infection (aOR 0.84 [CI:0.71–0.99]) and renal (aOR 0.51 [CI:0.39–0.66]) late effects. By contrast, the opposite trend was observed in survivors who received chemo-radiotherapy. High antimetabolite chemotherapy cumulative dose was associated with increased risks of subsequent cancer (aOR 2.32 [CI:1.06–4.84]), metastatic cancer (aOR 4.44 [CI:1.29–11.66]) and renal (aOR 3.48 [CI:1.36–7.86]) conditions. Patients who received radiation dose of ≥50 Gy experienced higher risks of developing metastatic cancer (aOR 5.51 [CI:2.21–11.86]), cancer (aOR 3.77 [CI:2.22–6.34]), haematological (aOR 3.43 [CI:1.54–6.83]) and neurological (aOR 3.24 [CI:1.78–5.66]) conditions. Similar trends were observed in survivors who received more than three teletherapy fields. Cumulative burden analyses on 183 conditions separately revealed varying dominance of different late effects across cancer types, socioeconomic deprivation and treatment modalities. Late effects are associated with excess YLL (i.e., the difference in YLL between survivors with or without late effects), which was the most pronounced among survivors with haematological comorbidities.

**Interpretation:**

To our knowledge, this is the first study to dissect and quantify the importance of late morbidities on subsequent survival using linked electronic health records from multiple settings. The burden of late effects is heterogeneous, as is the risk of premature mortality associated with late effects. We provide an extensive knowledgebase to help inform treatment decisions at the point of diagnosis, future interventional trials and late-effects screening centred on the holistic needs of this vulnerable population.

**Research in context**

**Evidence before this study**

We searched PubMed, Google Scholar and European PMC from database inception to 1 July 2021 for studies on late effects in children, teenagers and young adults who survived cancer. Population-based studies investigating a wide range of physical and mental health conditions were limited. Most studies have focused on a small group of late effects (e.g., cardiovascular or neurological events). Several studies have employed data from populations representing the United States and these may not be generalisable to other high-income country settings. Other studies have examined the morbidity using cancer registries without data linkage to general practices or hospitals. Importantly, most studies have not considered late effects managed in both primary care and hospital settings. Some studies used siblings as the control population. We did not identify any studies that investigate the disease burden by age, socioeconomic deprivation, detailed cancer treatment modalities for over a hundred diseases contemporaneously with a single linked dataset within a publicly funded healthcare system. We also did not identify any studies describing the burden of in-patient hospitalisation and critical care admissions in both survivors and controls.

**Added value of this study**

We present the first life course atlas of cancer survivorship, involving 183 physical and mental health conditions. We present the cumulative burden of late effects and hospitalisation stratified by cancer subtypes, socioeconomic deprivation and treatment modalities. Detailed code lists for all conditions are available open-access and although conditions were selected based on healthcare utilisation in England, they are relevant to other developed countries with similar demographics. This study employs clinically validated conditions from routine clinical care and is therefore agnostic to patients’ knowledge about a condition. Matched community controls were identified, allowing comparison of morbidity burden with survivors. We analysed records obtained from general practices using different electronic health record platforms (i.e., Vision® or EMIS® software systems); this means that our work is translatable to other platforms. Our dataset is linked to the Hospital Episode Statistics, the National Cancer Registration and Analysis Service, England index of multiple deprivation and the Office for National Statistics death registry. Our findings illustrate the varying contribution of different late effects according to age, primary cancer diagnosis and treatment during the survivorship phase. Socioeconomic differences in morbidity burden were also discernible. Survivors who developed late effects experienced premature mortality (i.e., excess years of life lost) compared with those without late effects.

**Implications of all the available evidence**

By charting the patterns of single and recurrent late effects during survivorship, this work could help empower young adults, parents and physicians to discuss potential long-term risks during the initial treatment consent phase. We present the cumulative burden of each 183 conditions individually and by organ system groups using open access electronic health record phenotypes on a real-world dataset. Survivors of leukaemia had the highest cumulative burden of late effects. Childhood cancer survivors had increased burden of in-patient hospital admissions and critical care admissions. Combined chemotherapy and radiotherapy, as well as treatment with antimetabolites were associated with increased burden of late effects and in-patient hospital admissions. Increased cumulative dose of antimetabolites, alkylating agents, plant alkaloids and antitumour antibiotics were associated with increased risk of certain late effects such as subsequent cancer, infection and immunological conditions, renal, endocrine, pulmonary and neurological conditions. Similarly, increased radiation dose and field were associated with increased risks of subsequent neoplasm and neurological conditions. Our knowledgebase on late effects and prognosis (excess years of life lost) could inform clinical guidelines on late effects screening, management and budget allocation in publicly funded healthcare systems. The heterogeneity in late effects could lead to future research into treatment for comorbidities. Disparities in disease burden between socioeconomic strata could instigate targeted policies addressing underserved and high-risk communities.

Alt-text: Unlabelled box

## Introduction

Although cancer is a major cause of death in children, teenagers and young adults, 5-year survival rates have remained high.^1^ Survivors can live well into adulthood but are at significant risk of late effects from their cancer or its treatment.^2^^,^^3^ The survivor population, however, is far from homogeneous, and given that many continue to live for decades, there is an urgent need to understand and systematically appraise previously unappreciated consequences of surviving cancer across a wide range of cancer types and disease outcomes. Cancer care is progressively adopting a model for chronic disease care. Health experiences within the long-term survivor population are likely to be different from those in the palliative or advanced disease phase. The cancer-as-chronic-disease care model requires coordination and involvement of general practitioners, specialists and multidisciplinary teams to meet the unique needs of the survivor population. The shift to chronic illness raises important points concerning patient empowerment in decision-making and awareness and monitoring of late effects^4^^,^^5^ Nonetheless, the risks of late effects are not always reviewed extensively in initial treatment consent discussions,^6^ but often during counselling sessions after completion of therapy and when patients enter the survivorship phase.^7^ While this is understandable in the face of a distressing diagnosis of childhood cancer where the initial priority is to achieve survival, most teenagers and young adults with cancer desire information about what could happen to them after cancer therapy^8^^,^^9^ and many want to be included in treatment decision-making at early stages.^10^^,^^11^ Yet, because their information needs regarding potential late effects are often unmet, participation in survivorship monitoring and care may be affected, hence causing impairments in long-term psychosocial and physical well-being. Studies have demonstrated that although receiving information on late effects can be distressing initially, teenagers and young adults considered such information to be important when deciding the best course of treatment.^12^^,^^13^ However, many felt that the information provided on late effects has been suboptimal compared with the extensive information they received about their cancer diagnosis. Unmet information needs are also linked to a lower quality of life during survivorship.^14^^,^^15^

Supplying information on late effects can encourage survivors to not only take control of treatment decisions, but also empower them to proactively engage with healthcare practitioners in survivorship care and to participate in late effects screening to help them adjust to life after cancer. Given the progress towards the cancer-as-chronic-disease care model, it is necessary to fully capture the burden of late effects across conditions managed in both primary care and hospitals, to provide tailored information about risk across healthcare settings. Utilising linked health records from four different settings (primary and secondary care, cancer registry and death registry) our study aims to address the burden of surviving cancer and associations of late effects with premature mortality. Specific objectives were: (i) to estimate the cumulative burden of 183 diseases by organ systems in cancer survivors and community controls, in the presence of death as a competing risk, (ii) to estimate the burden of in-patient hospitalisation and critical care admissions, (iii) to provide stratified cumulative burden estimates based on all cancer subtypes, socioeconomic deprivation status, treatment type and chemotherapy drug class, (iv) to estimate the association between treatment exposures and diagnosis of late effects and (v) to estimate excess years of life lost attributable to late effects. Since late effects risk communication practices may differ across diseases and healthcare settings, our results were generated from a wide range of primary care practices and hospitals to allow the generalisability of findings. Results may be used to facilitate informed decision-making at the point of cancer diagnosis and to support life after cancer.

## Methods

### Study design and data sources

We used linked electronic health records (EHRs) from primary care obtained from the Clinical Practice Research Datalink (CPRD). CPRD has two primary care data resources, GOLD and Aurum, containing routinely collected data from primary care practices in England. The full cohort consisted of 5,343,578 individuals (603,620 from GOLD and 4,739,958 from Aurum), during the study period of 01/01/1998 to 31/10/2020. Over 1400 and 300 primary care practices contribute to the Aurum and GOLD datasets, respectively.^16^ Data from GOLD and Aurum were linked to secondary care Hospital Episode Statistics (HES), patient-level Index of Multiple Deprivation (IMD), Office for National Statistics (ONS) death registry and the National Cancer Registration and Analysis Service (NCRAS). For HES linkage, we analysed data on in-patient admissions from the Admitted Patient Care (APC) dataset and critical care admissions from the Adult Critical Care (CC) dataset. For NCRAS linkage, we analysed data on cancer registration (containing detailed information on cancer site, morphology, behaviour and treatment). Within the NCRAS dataset, we explored the Systemic Anti-Cancer Treatment (SACT) dataset containing chemotherapy drug details, and the Radiotherapy (RTDS) dataset. Information governance approval was obtained from the Medicines Healthcare Regulatory Authority Independent Scientific Advisory Committee (19_222).

### Identification of children, teenagers and young adults with cancer and community controls

All individuals who had a primary cancer diagnosis at age ≤ 25 years were considered as the cancer population. Community control participants were identified by propensity score matching (PSM) by year of birth, sex, socioeconomic deprivation and primary care practice identifier. PSM was performed using the nearest-neighbour matching method (1:4, cancer survivors: control match) with a calliper width of 0.2 of the standard deviation of the logit of the propensity score. Follow-up for survivors started at age 18 years or 5 years from their primary cancer diagnosis, whichever occurred later. Follow-up for control participants started at age 18 years. At-risk status for individuals ended on 31/10/2020 (administrative censoring), date of deregistration from the practice or on the date of death, whichever occurred first.

### Electronic health records coding and phenotypes

Cancer classification codes in NCRAS were based on the International Classification of Childhood Cancer (ICCC-3) and morphology codes in ICD-O-3. Detailed cancer classification coding list was obtained from the 2021 children, teenagers and young adults UK cancer statistics report,^17^ where cancer diagnostic groups were identified based on a combination of morphology, behaviour and site codes. EHR phenotypes for 183 conditions were obtained from the open-access CALIBER phenotype library (https://portal.caliberresearch.org/) and have been previously validated.^18^, ^19^, ^20^ Phenotypes for CPRD GOLD were generated using version 2 Read codes. Phenotypes for CPRD Aurum data were generated using a combination of SNOMED CT, Read version 2 and EMIS Web codes. Phenotypes for HES were generated in ICD-10. The 183 conditions were classified into 13 organ system categories. Common Terminology Criteria for Adverse Events (CTCAE) was not used in calculating the cumulative burden and each event, regardless of medical complexity, was added uniformly and agnostic of severity.

We considered nine cancer treatment variables: all chemotherapy (i.e., everyone who received chemotherapy), all radiotherapy, all surgery, chemotherapy only (i.e., individuals who received chemotherapy only and nothing else), radiotherapy only, surgery only, chemotherapy and radiotherapy, chemotherapy and surgery and radiotherapy and surgery. We considered nine types of chemotherapy drug variables: alkylating agents, anthracyclines, antimetabolites, chemotherapy unspecified, hormonal agents (including corticosteroid hormones and sex hormones), non-anthracycline antitumour antibiotics, plant alkaloids and natural products (excluding vinca alkaloids), platinum agents, vinca alkaloids.

### Statistical analyses

The 183 conditions were processed using previously described event subtypes based on definitions of chronicity and recurrence.^21^, ^22^, ^23^ Each condition was assigned to one of the three event subtypes: i) single, recurrent events that can occur multiple times (e.g., stroke or myocardial infarction), ii) chronic, non-recurrent events that is considered only once at the time of disease onset (e.g., fatty liver disease or diabetes) and iii) chronic, recurrent events (e.g., cardiomyopathy or oesophageal varices). With regards to how prevalent conditions diagnosed prior to patients entering the cohort were handled, we have only captured health events that occurred during the follow-up period. For prevalent conditions that have been resolved before patients enter the cohort (no subsequent health events for that condition are observed during follow-up), these conditions were not captured. For prevalent conditions that continued to demonstrate health events during follow- up, events during follow-up will be included. Cumulative burden was estimated using the previously described and validated mean cumulative count (MCC) method.^21^^,^^24^ For example, a cumulative burden/MCC of 0.73 for renal disease per individual by age 35 means that there would be an average of 0.73 renal disease events occurring per individual, which can also be interpreted as an average of 73 renal disease events occurring per 100 individuals. Unlike cumulative incidence which estimates the cumulative probability of developing an event by considering only the first occurrence of the event for each individual, the MCC method summarises all events that occurred in a population by a given time and not just the first event.^24^ The MCC method allowed us to analyse the burden of recurrent events in the presence of competing risks within a specified time period. Death was considered a competing-risk event as it precludes the occurrence of the health event of interest. Unlike cumulative incidence which ranged from 0 to 1, MCC can be any positive number as it estimates the mean count of events per individual within a certain population rather than the probability of developing the event of interest. We estimated MCC for 183 conditions grouped by organ system categories and 95% confidence intervals (CIs) were generated using the bootstrap percentile method.^24^ For conditions grouped by organ systems, cumulative burden per individual was shown. However, for each of the individual 183 conditions, due to increased granularity, cumulative burden per 100 individuals was shown. Furthermore, MCC calculations for survivors accounted for left truncation because survivors can enter the cohort at different ages.^25^

We performed logistic regression to determine the associations between treatment exposures and diagnosis of health conditions. Models were adjusted for age at cancer diagnosis, cancer subtype, sex and deprivation status. Years of life lost (YLL) describes the number of years lost due to premature mortality and was estimated using the R package lillies,^26^ which was validated by other studies.^27^, ^28^, ^29^ We estimated excess YLL based on the specific age of disease onset at ages 32.5, 35, 37.5, 40, 42.5 and 45. Excess YLL denotes the difference in YLL between two groups: survivors who developed a health condition minus survivors who did not develop a health condition.

Data were analysed using R (3.6.3) with the following packages: tidyverse, tableone, lillies, reshape, splines, survival, etm, mstate and cmprsk.

The funders did not have any role in the study design, data collection, data analysis, interpretation, or writing of the manuscript.

## Results

**Figure 1:**
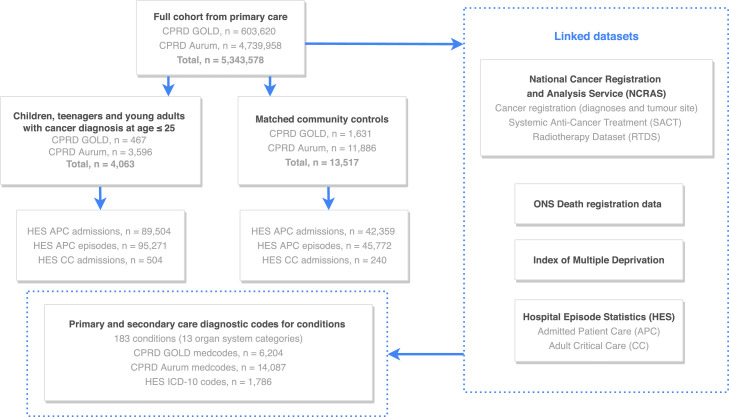
Flow diagram depicting linked electronic health records from primary care linked to secondary care hospital episode statistics (in-patient admissions and critical care admissions), cancer registry, death registry and patient-level deprivation data. Number of children, teenager and young adults with cancer and matched community controls is provided. Two primary care cohorts are used: CPRD GOLD and CPRD Aurum. The number of diagnostic and medical codes (medcodes) used for 183 conditions is provided. ONS: Office for National Statistics.

We identified 4063 children, teenagers and young adults with a cancer diagnosis at age ≤ 25 years. Of these individuals, 3466 (85%) survived for at least 5 years from the date of diagnosis and were 18 years or older (Table S1). Community controls (*n* = 13,517) matched to cancer survivors were obtained. Survivors had a total of 89,504 in-patient hospital admissions and 504 critical care admissions, while controls had 42,359 in-patient admissions and 240 critical care admissions (Fig. 1). Follow up duration were as follow: cancer survivors (median: 6.75 years, IQR: 8.67 years) and controls (median: 9.65 years, IQR: 8.92 years). Patient characteristics of all cancer patients and survivors are presented in Table S1. A map of each result to their corresponding dataset(s) is presented in Figure S1.

### Cancer survivors had an overall higher burden of disease compared with community controls

**Figure 2:**
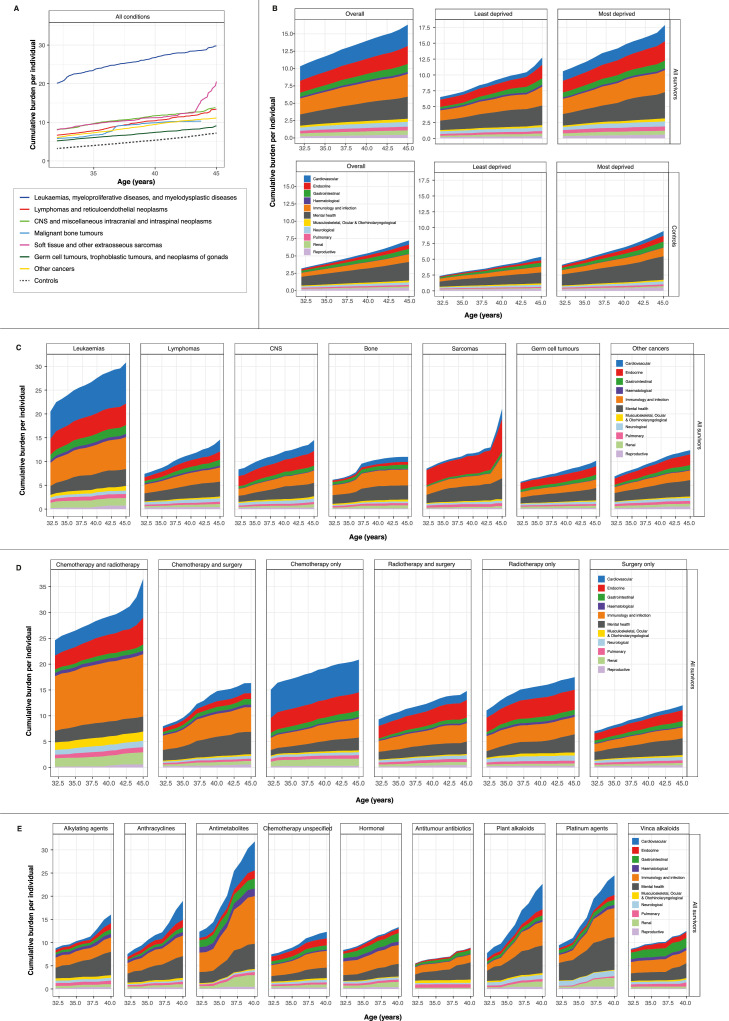
Cumulative burden of health conditions. (A) Cumulative burden of all conditions in cancer survivors (by cancer diagnostic groups) and community controls. (B) Distribution of cumulative burden of health conditions by organ systems. Cumulative burden is shown for the overall population and for least deprived and most deprived individuals. (C) Distribution of cumulative burden of health conditions by organ systems among survivors stratified by primary cancer diagnosis. (D) Distribution of cumulative burden of health conditions by organ systems among survivors stratified by type of cancer treatment. (E) Distribution of cumulative burden of health conditions by organ systems among survivors receiving chemotherapy stratified by chemotherapy type. All data and 95% confidence intervals are provided in the supplementary tables. Other cancers include adrenocortical carcinomas, carcinomas of bladder, carcinomas of breast, carcinomas of cervix uteri, carcinomas of colon, carcinomas of salivary glands, malignant melanomas, nasopharyngeal carcinomas, skin carcinomas and thyroid carcinomas. Cumulative burden is expressed as mean cumulative count per individual for each condition-specific outcome. For example, a cumulative burden of 2.5 at age 35 means that there is an average of 2.5 events occurring per person at age 35.

We analysed the cumulative burden of 183 health conditions (Table S2) which consisted of 6204 (CPRD GOLD) and 14,087 (CPRD Aurum) diagnostic codes from primary care and 1786 ICD-10 codes from secondary care. When considering all conditions, the cumulative burden at age 35 years was the highest in survivors of leukaemia (23.52 [19.85–29.33]) and lowest in survivors of germ cell tumours (6.04 [5.32–6.91]). Trends in cumulative burden were maintained at age 45 years: leukaemia (29.79 [24.66–35.95]) and germ cell tumours (9.07 [7.96–9.91]). By contrast, in the controls, the cumulative burden was 3.99 (3.93–4.08) at age 35 years and 7.19 (7.10–7.36) at age 45 years (Fig. 2A; Table S3).

### Cardiovascular and immunological conditions or infections are important late effects among survivors

We analysed cumulative burden for conditions by organ systems among survivors and observed that the burden was highest for immunological conditions and infections (age 35, 2.52 [2.14–2.98]; age 45, 3.27 [3.01–3.58]), followed by cardiovascular conditions (age 35, 2.25 [1.11–2.41]; age 45, 3.08 [1.98–3.29]) (Fig. 2B; Table S4). By contrast, in controls, the cumulative burden for immunological conditions and infections was 0.68 (0.67–0.71) at age 35 and 1.12 (1.06–1.17) at age 45. For cardiovascular conditions, the cumulative burden in controls was 0.19 (0.17–0.22) at age 35 and 0.61 (0.54–0.67) at age 45. In community controls, cumulative burden was the highest for mental health conditions: age 35 (1.56 [1.51–1.72]) and age 45 (2.68 [2.46–2.87]). Nonetheless, cancer survivors experienced an even higher burden of mental health conditions: age 35 (2.02 [1.79–2.12]) and age 45 (3.19 [2.95–3.40]) (Fig. 2B; Table S4).

### Deprivation status is an important indicator of the burden of diseases

Most deprived individuals (IMD 5) had an overall higher burden of health conditions compared with least deprived individuals (IMD 1) in both survivors and controls. For example, at age 35, cumulative burden for cardiovascular conditions was almost four times higher in most deprived survivors (1.63 [1.06–2.11]) compared with least deprived survivors (0.47 [0.35–0.59]) (Fig. 2B; Table S5). Similarly, as with all other conditions, cumulative burden for endocrine conditions at age 35 was higher in most deprived survivors (2.03 [1.43–2.57]) compared with least deprived survivors (1.25 [1.07–1.48]). The trend of a higher cumulative burden in most deprived individuals was also mirrored in the controls. For example, at age 45, the cumulative burden for gastrointestinal conditions was almost twice as high in most deprived controls (0.90 [0.75–0.95]) compared with least deprived controls (0.57 [0.50–0.64]) (Fig. 2B; Table S5).

### Variations in cumulative burden across cancer diagnostic groups and organ systems

When looking across cancer types, the cumulative burden of cardiovascular conditions increased with age to different extents. The greatest increase (when comparing age 35 with age 45) was in survivors of soft tissue sarcomas (430% increase from 0.41 at age 35 to 2.17 at age 45), followed by bone tumours (230% increase from 0.32 to 1.07), lymphoma (199%), germ cell tumours (193%), other cancers (58%), CNS tumours (41%) and leukaemia (35%) (Fig. 2C; Table S6). Among lymphoma survivors, after cardiovascular conditions, the increase in cumulative burden for other organ systems from age 35 to age 45 were as follow: gastrointestinal (107% from 0.59 to 1.22), renal (104%), endocrine (65%), musculoskeletal, ocular and otorhinolaryngological (55%), mental health (53%), immunology and infection (47%), pulmonary (37%), haematological (32%), neurological (31%) and reproductive (16%) (Fig. 2C; Table S6).

### Variations in cumulative burden of diseases across cancer treatment exposures

The highest cumulative burden of diseases was observed in survivors who received both chemotherapy and radiotherapy, while the lowest disease burden was found in survivors who received only surgery (Fig. 2D). Among survivors who received chemotherapy and radiotherapy, the cumulative burden of diseases by organ systems at age 45 ranked from highest to lowest were: immunology and infection (12.06 [6.9–16.86]), cardiovascular (7.52 [4.12–12.4]), endocrine (5.18 [3.43–6.36]), mental health (2.91 [1.62–4.2]), renal (2.35 [1.96–3.21]), musculoskeletal, ocular and otorhinolaryngological (1.77 [1.1–2.99]), neurological (1.27 [0.75–1.68]), gastrointestinal (1.19 [0.66–1.59]), pulmonary (0.98 [0.73–1.14]), haematological (0.68 [0.52–0.82]) and reproductive (0.58 [0.15–1.02]) (Fig. 2D; Table S7). By contrast, among survivors who received surgery only, the cumulative burden of diseases at age 45 were: mental health (3.27 [2.76–3.79]), immunology and infection (2.12 [1.88–2.3]), endocrine (2.11 [1.67–2.34]), gastrointestinal (1.01 [0.93–1.17]), cardiovascular (0.93 [0.77–1.15]), neurological (0.67 [0.61–0.8]), pulmonary (0.51 [0.47–0.56]), renal (0.43 [0.4–0.53]), reproductive (0.43 [0.36–0.49), musculoskeletal, ocular and otorhinolaryngological (0.35 [0.32–0.37]) and haematological (0.2 [0.13–0.27] (Fig. 2D; Table S7).

### Survivors who received antimetabolites for chemotherapy had the highest disease burden

We estimated cumulative burden by chemotherapy drug classes and found that survivors treated with antimetabolites had the highest disease burden followed by those treated with platinum agents and plant alkaloids (excluding vinca alkaloids). Among survivors who were treated with antimetabolites, cumulative burden of diseases at age 40 ranked from highest to lowest were: immunology and infection (10.27 [7.06–14.92]), cardiovascular (6.23 [3.23–8.32]), mental health (5.45 [2.74–7.11]), renal (2.51 [0.7–4.19]), gastrointestinal (2.15 [0.73–2.4]), endocrine (1.77 [0.72–2.11]), haematological (1.64 [0.98–1.89]), pulmonary (0.67 [0.56–1.07]), reproductive (0.46 [0.27–0.68]), neurological (0.41 [0.24–0.53]) and musculoskeletal, ocular and otorhinolaryngological (0.25 [0.1–0.42]) (Fig. 2E; Table S8). By contrast, survivors who received antitumour antibiotic treatments (excluding anthracyclines) had the lowest cumulative burden at age 40: mental health (3.70 [2.73–6.27]), immunology and infection (1.69 [1.65–2.35]), gastrointestinal (0.84 [0.39–0.93]), pulmonary (0.75 [0.54–1.19]), musculoskeletal, ocular and otorhinolaryngological (0.72 [0.36–1.21]), endocrine (0.39 [0.36–0.64]), neurological (0.29 [0.21–0.45]), renal (0.18 [0.11–0.29]), haematological (0.15 [0.08–0.24]), cardiovascular (0.10 [0.04–0.15]), reproductive (0.07 [0.02–0.09]) (Fig. 2E; Table S8).

### Survivors had a higher cumulative burden of in-patient and critical care admissions, which is exacerbated by socioeconomic deprivation

**Figure 3:**
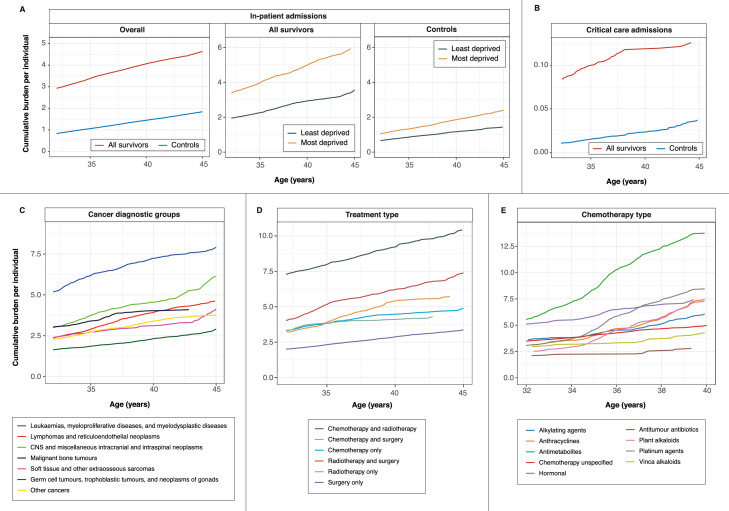
Cumulative burden of hospitalisation. (A) Cumulative burden of in-patient hospital admissions in cancer survivors and community controls. Cumulative burden is shown for the overall population and for least deprived and most deprived individuals. (B) Cumulative burden of critical care admissions. (C) Cumulative burden of in-patient admissions among cancer survivors stratified by primary cancer diagnosis. (D) Cumulative burden of in-patient admissions among cancer survivors stratified by treatment type. (E) Cumulative burden of in-patient admissions among cancer survivors receiving chemotherapy stratified by chemotherapy type. All data and 95% confidence intervals are provided in the supplementary tables. Other cancers include adrenocortical carcinomas, carcinomas of bladder, carcinomas of breast, carcinomas of cervix uteri, carcinomas of colon, carcinomas of salivary glands, malignant melanomas, nasopharyngeal carcinomas, skin carcinomas and thyroid carcinomas.

Survivors had a higher burden of in-patient admissions compared with community controls. Cumulative burden of in-patient admissions among survivors at ages 35, 40 and 45 were as follow: age 35 (3.38 [3.18–3.59]), age 40 (4.06 [3.97–4.21)) and age 45 (4.64 [4.60–4.82]). Cumulative burden of in-patient admissions among controls at ages 35, 40 and 45 were as follow: age 35 (1.06 [1.04–1.07]), age 40 (1.45 [1.40–1.47]) and age 45 (1.84 [1.82–1.88]). (Fig. 3A; Table S9). Most deprived individuals had a higher burden of in-patient admissions in both survivors and controls. In survivors, at age 45, most deprived individuals had a cumulative burden of 5.92 (5.69–6.66) compared with least deprived individuals (3.58 [3.03–4.28]). Critical care admissions remained low overall; however, survivors had a higher burden of critical care events compared with controls at all ages (Fig. 3B; Table S9). At age 45, the cumulative burden of critical admissions in survivors was 0.126 (0.112–0.138) compared with 0.037 (0.031–0.042) in controls.

### Survivors of leukaemia and cns malignancies had some of the highest burden of in-patient admissions

Cumulative burden of in-patient admissions was the highest in survivors of leukaemia: age 35 (6.02 [5.60–6.33]), age 40 (7.24 [6.61–7.36]) and age 45 (7.91 [7.00–8.05]). This was followed by survivors of CNS malignancies: age 35 (3.74 [3.49–3.87]), age 40 (4.54 [4.09–4.67]) and age 45 (6.13 [4.52–6.80]). Other cancer types had a lower burden of in-patient admissions. At age 45, the cumulative burden of in-patient admissions was as follow: lymphomas (4.62 [3.96–4.74]), soft tissue sarcomas (4.15 [4.46–7.84]), bone tumours (4.09 [3.01–4.61]), other cancers (3.77 [3.28–4.55]) and germ cell tumours (2.91 [2.46–3.44]) (Fig. 3C; Table S10).

### Survivors who received chemotherapy and radiotherapy treatment had the highest burden of in-patient admissions

Cumulative burden for in-patient admissions among survivors who received chemotherapy and radiotherapy treatment was the highest across all ages: age 35 (7.99 [5.80–9.18]), age 40 (9.22 [7.26–10.83]) and age 45 (10.43 [8.27–11.95]). This was followed by survivors who received radiotherapy and surgery: age 35 (5.16 [4.61–6.79]), age 40 (6.22 [5.81–8.08]) and age 45 (7.38 [6.68–9.13]). By contrast, cumulative burden for in-patient admissions was the lowest in survivors who only received surgery: age 35 (2.32 [2.17–2.45]), age 40 (2.87 [2.66–2.91]) and age 45 (3.37 [3.16–3.42]). (Fig. 3D; Table S11).

### Variations in cumulative burden of in-patient admissions among survivors who received chemotherapy

Survivors who received antimetabolite chemotherapeutic drugs had the highest cumulative burden for in-patient admissions at age 40 (13.76 [8.51–18.41]). For other chemotherapeutics, cumulative burden for in-patient admissions were as followed: platinum agents (8.46 [5.20–10.53]), anthracyclines (7.48 [6.60–8.12]), hormonal agents (7.41 [5.84–7.71]), plant alkaloids (excluding vinca alkaloids) (7.31 [5.34–8.14]), alkylating agents (6.08 [5.11–7.16]), vinca alkaloids (4.29 [3.48–4.64]) and non-anthracycline antitumour antibiotics (2.79 [2.09–3.28]) (Fig. 3E; Table S12).

### Multivariable regression analyses for the association between treatment exposures and diagnosis of health conditions

**Figure 4:**
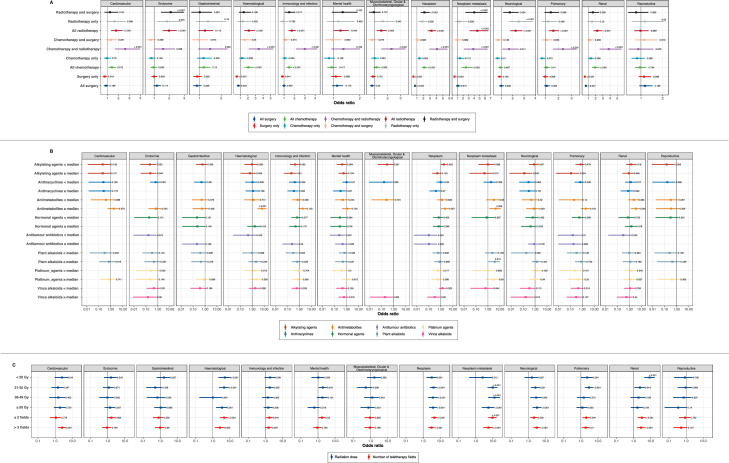
Multivariable logistic regression analysis of health condition outcomes among cancer survivors by different exposures. (A) Treatment type. (B) Chemotherapy dose. Patients were stratified by median values of cumulative dose of different chemotherapeutic agents. (C) Radiotherapy dose and field. Teletherapy fields are denoted as the actual number of fields used to deliver a fraction. Radiation dose is denoted in Grays. Forest plots indicate adjusted odds ratios and error bars represent 95% confidence intervals for the odds ratio. P values are indicated on the plot. Strata with low number of outcome events (*n*<5) were not analysed.

After adjusting for age at diagnosis, cancer subtype, sex and deprivation status, survivors who received surgery only had lower odds of developing cardiovascular (adjusted odds ratio 0.73 [0.56–0.94]), haematological (0.51 [0.37–0.70]), immunology and infection (0.84 [0.71–0.99]) and renal (0.51 [0.39–0.66]) late effects (Fig. 4A; Table S13). By contrast, all survivors who received radiotherapy had a higher odds of developing cardiovascular (1.78 [1.33–2.36]), endocrine (1.99 [1.57–2.50]), haematological (2.13 [1.53–2.92]), immunology and infection (1.75 [1.42–2.14]), mental health (1.26 [1.01–1.58]), musculoskeletal, ocular and otorhinolaryngological (1.51 [1.16–1.94]), neurological (2.27 [1.79–2.86]), pulmonary (1.61 [1.26–2.04]) and renal (2.23 [1.70–2.91]) late effects. Similarly, survivors who received chemotherapy and radiotherapy also had higher odds of developing cardiovascular (2.62 [1.67–3.97]), endocrine (1.59 [1.04–2.38]), haematological (3.43 [2.12–5.34]), immunology and infection (3.00 [2.14–4.19]), musculoskeletal, ocular and otorhinolaryngological (2.30 [1.52–3.38]), neurological (1.69 [1.09–2.53]), pulmonary (2.34 [1.58–3.39]) and renal (3.83 [2.57–5.60]) conditions (Fig. 4A; Table S13).

### Multivariable regression analyses for the association between chemotherapy cumulative dose and radiotherapy dose and field and diagnosis of health conditions

Survivors who received higher (above median) dose of antimetabolites experienced higher risks of developing renal late effects (adjusted odds ratio 3.48 [1.36–7.86]), cancer (2.32 [1.06–4.84]) and metastatic cancer (4.44 [1.29–11.66]) (Fig. 4B, Table S14). In contrast, survivors who received higher dose of alkylating agents had lower risks of developing the following late effects: immunology and infection (0.21 [0.05–0.58]), pulmonary (0.12 [0.01–0.53]), neurological (0.13 [0.01–0.58]) and endocrine (0.23 [0.04–0.76]).

Increasing dose of radiation was associated with increased risks of second neoplasm, metastatic cancer and neurological late effects (Fig. 4C, Table S15). Patients who received radiation dose of 50 Gy or higher experienced higher risks of developing metastatic cancer (5.51 [2.21–11.86]), cancer (3.77 [2.22–6.34]), haematological (3.43 [1.54–6.83]) and neurological (3.24 [1.78–5.66]) conditions. Survivors who received more than three teletherapy fields experienced increased risks of developing metastatic cancer (5.53 [2.76–10.27]), cancer (3.06 [2.01–4.60]), neurological (3.10 [1.95–4.82]), renal (2.79 [1.59–4.65]), haematological (2.58 [1.31–4.68]), cardiovascular (2.58 [1.45–4.32]) and pulmonary (1.90 [1.14–3.04]) late effects.

### Cumulative burden of condition-specific outcomes for 183 conditions

We estimated the cumulative burden of 183 conditions separately at age 45 years. Conditions were ranked according to cumulative burdens in controls. Mental health, bacterial infections and hypertension were ranked highly in survivors and controls (Figure S2, Table S16). Recurrent or secondary neoplasms, cardiovascular conditions were ranked highly among survivors. Survivors of leukaemia had high cumulative burden of hypertension (146.40 per 100 individuals [90.05–150.77]), hypertrophic cardiomyopathy (156.75 [14.14–235.05]) and heart failure (33.10 [19.18–42.92]). Metastatic cancer was common among survivors of bone cancer: metastasis of the lungs (244.36 per 100 individuals [75.12–283.29]), bowel (231.76 [162.19–319.06]), liver (231.21 [162.19–317.93]) and brain (55.07 [24.38–79.42]).

Cumulative burdens of health conditions were markedly higher in survivors and controls with high socioeconomic deprivation (Figure S2, Table S17). The cumulative burdens for asthma in controls and survivors from the least deprived areas were 12.42 per 100 individuals (11.31–12.68) and 21.78 (19.63–23.94), respectively. For those living in the most deprived areas, the cumulative burdens were 16.73 (15.37–16.86) in controls and 30.89 (26.26–34.06) in survivors. Similar trends were observed across a range of conditions such as obesity, diabetes, infections, cancer, myocardial infarction, liver disease and more.

When comparing across cancer treatment modalities, cumulative burdens of second neoplasms were consistently high (Figure S2, Table S18). Other non-cancer conditions that were ranked highly include hyperparathyroidism, diabetic ophthalmic and neurological complications, hypo or hyperthyroidism, hepatic failure, end stage renal disease and heart failure. A similar observation was found when comparing across chemotherapeutic agents (Figure S2, Table S19).

### Cumulative burden of 25 infections and immunological conditions

Earlier analyses by organ system groups revealed high cumulative burden of infections and immunological conditions among survivors (Fig. 2). We performed additional stratified analyses on 25 conditions separately to ascertain whether the burden of these conditions was associated with cancer recurrence or subsequent cancer. Survivors who developed subsequent cancers had very high disease burden, followed by survivors who faced cancer recurrence (Figure S3, Table S20). At age 45 in survivors who had subsequent cancer, the cumulative burdens were as follow: bacterial infections (2.71 per individual [2.31–2.41]), infections of other or unspecified organs (1.48 [1.04–2.05]), infections of the digestive system (1.29 [1.15–2.50), lower respiratory tract infections (1.04 [0.84–1.19]), infection of the skin (0.82 [0.61–0.99]), urinary tract infections (0.72 [0.45–0.81]) and septicaemia (0.70 [0.49–0.88]).

### Survivors who developed late effects experienced premature mortality

**Figure 5:**
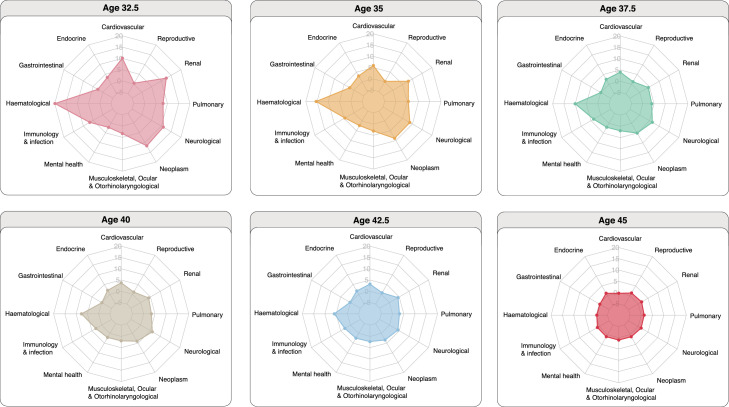
Excess years of life lost (YLL) attributable to health conditions (grouped by organ systems) among cancer survivors. Radar plots depict the difference in years of life lost between two groups: survivors who developed a health condition compared with survivors who did not develop a health condition. Excess YLL was estimated based on the specific age of onset of the health condition. All data and 95% confidence intervals are provided in the supplementary tables.

We estimated excess years of life lost (YLL) which is calculated as the average number of years that survivors with late effects lose in excess of that found in survivors without late effects of the same age. Excess YLLs were estimated based on the age of onset of the health condition. Excess YLLs were displayed as radar plots to allow comparison across conditions grouped by organ systems (Fig. 5). When evaluating the surface areas covered in each radar plot, younger age of disease onset was associated with higher excess YLL (larger surface areas). As the age of disease onset increased, excess YLL decreased. Survivors who developed haematological conditions experienced the highest excess YLL compared with other late effects. At age 32.5 years, excess YLLs ranked from highest to lowest were as follow: haematological (19.93 years [15.33–27.34]), renal (12.50 [8.46–16.33]), neoplasm (11.67 years [9.29–15.27]), neurological (10.98 [7.27–13.34]), cardiovascular (10.13 [7.13–14.30]), pulmonary (8.07 [6.07–11.65]), immunology and infection (6.72 [5.14–10.90]), endocrine (3.36 [1.25–8.47]), musculoskeletal, ocular and otorhinolaryngological (3.33 [1.61–4.01]), gastrointestinal (2.57 [0.64–5.20]), mental health (2.22 [0.28–3.72]) and reproductive (0.42 [−1.83–2.29]) (Fig. 5; Table S21).

## Discussion

Harnessing linked electronic health records from primary care, secondary care, the cancer registry, death registry and deprivation records from the Office for National Statistics, we believe that our study represents the most comprehensive, population-based assessment of long-term late effects in children, teenagers and young adults who survived cancer. We demonstrate that cancer survivors are a heterogeneous group where the extent of late effects differ across cancer subtypes, deprivation status, treatment exposures and chemotherapy drug classes. Compared with community controls, survivors notably had a higher risk of morbidity regardless of their primary cancer diagnosis and deprivation status. Furthermore, with detailed treatment data, we were able to ascertain the degree of heterogeneity in cumulative burden of late effects and hospitalisation. Late effects may arise as a long-term result of cancer treatment or from the cancer itself (progression or relapse). By estimating disease-specific burden involving a wide range of conditions, we provide an extensive resource that may help in the design and implementation of future interventional trials focusing on maximising patient safety while ensuring antineoplastic efficacy.

Although this study has reinforced the longstanding view that late effects are common among cancer survivors, we believe that the novelty of our study lies in the following areas. First, there has been no large-scale analysis on late effects for 183 diseases contemporaneously using a single real-world linked dataset from general practices and hospitals within a universal healthcare system. Most studies have focused on a limited number of conditions, which does not yield a comprehensive blueprint of childhood cancer survivorship that reflects the disease burden and healthcare utilisation of England, which are likely representative of other countries with similar population structures and economies. Second, our study is the first to provide detailed cumulative burden estimates for each of the 183 diseases separately as well as estimates by organ system categories. Our results demonstrate the varying dominance of different conditions across cancer types and treatment modalities. By providing estimates for each health condition across survivorship, we believe that this study will empower patients and their families, physicians, researchers and policymakers to develop better strategies to identify and treat individuals who are most at risk. Third, to the best of our knowledge, no other studies on late effects have employed linked real-world datasets from multiple sources (primary care, secondary care, cancer registry and death registry). This is because these digital resources employ different coding schemes and the construction of case definitions and codelists across these resources is a limiting factor. Building on initial phenotyping work,^20^ this study utilised open access EHR codelists to return cumulative burden estimates for 183 conditions, laying the groundwork for future studies on multimorbidity, which becomes increasingly more common as cancer survivors age. Fourth, among cancer survivors, we noticed high cumulative burden for infections and immunological conditions. Detailed analyses on 25 infections and immunologic conditions revealed that disease burden was the highest among survivors who developed subsequent cancer and the lowest in survivors who did not have cancer recurrence or subsequent cancers. The Centers for Disease Control and Prevention (CDC) launched the *Preventing Infections in Cancer Patients* campaign, and our work may facilitate conversations between physicians and patients on best practices to prevent, identify and treat potentially life-threatening infections. Fifth, we reported the risks of developing specific late effects by cancer treatment type, chemotherapy cumulative dose and radiotherapy dose and field. Such information may be reviewed at the initial treatment consent phase to provide patients with details on what they could potentially face before deciding on a specific treatment plan. Sixth, we are not aware of any studies reporting excess YLL by age of late effects onset. Information on prognosis may help physicians prioritise and treat conditions that pose the greatest risks to long-term survival.

Combining radiotherapy with systemic chemotherapy may often lead to improved therapeutic outcomes because the systemic effect of chemotherapy helps sensitise cancer cells to radiation, leading to better disease-free rates and overall survival compared with patients receiving chemotherapy or radiation alone.^30^ However, we have shown that survivors who received both therapies had a significantly higher burden of morbidity and in-person hospitalisation events later in life, suggesting that although chemo-radiotherapy is effective in improving overall survival rates, it is associated with long-term toxicity and lower quality of life. Furthermore, survivors who received chemo-radiotherapy had significantly higher risks of developing second neoplasms (localised and metastasised) and the risk of second neoplasm increased with increasing radiation dose and teletherapy fields.

As our study investigates changes in cumulative burden over time, we could distinguish early-onset morbidities from late-onset morbidities. For example, in survivors of leukaemia and lymphoma, cardiovascular morbidities increased more rapidly over time as individuals age compared to other diseases. On the other hand, cumulative burden for neurological and gastrointestinal conditions among survivors of CNS malignancies have remained relatively stable over time, suggesting that they might be early-onset morbidities directly arising from the toxic effects of cancer treatment. Survivors treated with antimetabolites experienced a dramatic increase in late-onset morbidities as they age, particularly for cardiovascular, renal and immunological conditions or infections. However, cumulative burden of gastrointestinal, neurological and pulmonary conditions remained stable over time, which highlights differing healthcare requirements in this population to ensure that stable conditions are appropriately managed while individuals are proactively screened for late-onset morbidities.

We observed that endocrinopathies (e.g., diabetes and obesity) were common in survivors of leukaemia, which could be a result of prolonged treatment with steroids.^31^^,^^32^ Survivors who received radiotherapy also had a high burden of endocrine disorders; hypothyroidism is reported to be a common late effect of radiation exposure.^33^ We found that survivors treated with anthracyclines were susceptible to late-onset cardiac morbidities, and another study demonstrated that cardiomyopathies could present as late as two decades after treatment.^34^ Chemotherapy often results in late hepatic and gastrointestinal sequelae.^35^^,^^36^ We found that survivors treated with vinca alkaloids and antimetabolites had a high burden of gastrointestinal conditions. Because hepatic dysfunction can go undiagnosed due to delayed manifestation, frequent monitoring of liver function enzymes and screening for viral hepatitis is useful to identify indolent liver disease.

Cancer is a common late effect in adult survivors of childhood cancer. Our analyses on 27 site-specific cancers and 10 metastatic cancers demonstrated that survivors experienced significant burden and risk of cancer with substantial variability by primary childhood cancer type, previous cancer treatment type and chemotherapy type. Our findings that subsequent cancer risk in childhood cancer survivors remained elevated in the long-term survivorship phase were consistent with studies performed in Australia, US, Europe and North America.^37^, ^38^, ^39^, ^40^, ^41^, ^42^ We observed that survivors who developed subsequent cancers or had cancer recurrence had a high burden of infections and immunological conditions. We found that bacterial infections were the most common, which may be a result of immunosuppression or neutropenia caused by subsequent cancer or its therapy, graft versus host disease after bone marrow transplant or the breakdown in skin barriers during catheterisation. Gram-positive bacteria account for >50% of infections in patients with cancer^43^ and infection with resistant microorganisms are common.[44] Bacterial infection could lead to poorer survival outcomes^45^ and efforts aimed at mitigating the impact of infections through targeted screening or decolonisation strategies while maintaining judicious use of antimicrobial agents to minimise resistance may be appropriate.^46^^,^^47^

### Strengths and limitations

First, our study employs a clinically important method of estimating the scale of disease burden over time. Most analyses routinely quantify cumulative incidence, which only considers the first event and therefore underestimating the total burden of disease. The cumulative burden approach overcomes this limitation as it considers recurrent events in the presence of competing risks, allowing the quantification of the total burden of events within populations.^24^ Second, earlier studies have relied on a small number of community controls (e.g., two previous reports relied on only 272 controls).^21^^,^^22^ while our cohort consisted of 13,517 matched controls. Given that controls were selected from a wide range of primary care practices, we were not only able to achieve a higher precision when estimating disease burden but also ensure that controls are representative of the general population. Third, other studies have used data collected from a limited setting; for example, data from a single research hospital.^21^^,^^22^ By contrast, we have used a population-based cohort that not only includes conditions that are managed in a general practice setting, but also conditions that require specialist input in hospitals, hence allowing the generalisability of our findings across clinical settings. Furthermore, in another study, control participants were censored one day after completing their clinical assessment visit.^21^ Since our study is based on data originating from routine clinical practice, we were able to analyse a diverse range of health conditions in controls and survivors over time, overcoming limitations in long-term survivorship research. Fourth, many cohort studies are limited to self-reported late effects that have not been clinically validated.^48^, ^49^, ^50^ Our study explored a diverse set of medically validated conditions covering major organ systems in both survivors and controls, overcoming the biases of self-reporting that rely on an individual's awareness of a condition. Fifth, our study utilised health records from general practices and hospitals, that are linked to the national cancer registry (NCRAS), which contains complete information about cancer and its treatment. Detailed information on neoplasm site, behaviour and morphology are available, allowing accurate categorisation into appropriate diagnostic groups. This is important because, unlike adult cancers, classification of childhood cancers has a greater emphasis on tumour morphology rather than primary site. NCRAS collects data from a wide range of health services (including hospices, screening services, histopathology and haematology services) to ensure complete cancer case ascertainment. Sixth, our cumulative burden and regression analyses incorporate socioeconomic deprivation indicators. This allows the identification of high-risk and underserved communities for targeted monitoring.

We acknowledge several limitations. We have not considered ethnic differences in cumulative burden of late effects due to insufficient data. Tumour stage was not considered due to high degree of missing data. Tumour stage may affect the type of treatment being prescribed and the extent of cancer progression, both of which could influence morbidity burden. Another limitation is that we have considered death from any cause as a competing risk event. We have not explored cause-specific mortality in this study and have not considered death from a specific disorder as an event of interest. We acknowledge that there could be surveillance bias between cancer survivors and community controls as survivors are more likely to have contact with healthcare services and therefore more likely to be diagnosed with a health condition. Nonetheless, our work includes primary care records which serve to mitigate surveillance bias to some degree as these records may serve as a more complete source for case ascertainment given that most individuals in England are registered with a GP. We recognised that there may be residual unmeasured confounding as with all observational studies. Future access to specialist disease registries such as the Myocardial Ischaemia National Audit Project may help improve case ascertainment for acute myocardial infarction.^51^ We note the large estimations of excess years of life lost in survivors who developed certain conditions such as haematological disorders. Although the estimates remain plausible, we felt that it was useful to highlight this observation as a limitation and include a note of caution in the interpretation of the results. There has been very limited research in this area, thus future work should provide additional information to help with results interpretation.

### Implications for parents, young adults, physicians and policymakers

Cardiovascular and immunological conditions or infections are common late effects among cancer survivors. Individuals from the most deprived regions had the highest disease burden and in-patient admissions, as do patients who received both chemotherapy and radiotherapy. Increased chemotherapy cumulative dose was associated with increased risks of subsequent cancer and renal late effects. Similarly, radiation dose of ≥50 Gy was associated with higher risk of subsequent metastatic cancer, haematological and neurological conditions. There has been limited research on how cancer therapies can be designed to minimise late effects, which warrants a separate investigation in the near future. Cumulative burden and risk estimates could promote awareness of long-term health risks in survivors and facilitate care as children transition to an adult care setting. Results may contribute to the development of follow-up guidelines for screening of asymptomatic survivors based on cancer therapeutic exposures to enable earlier identification and intervention of late effects. Unlike in the USA where access to health services is dependent on insurance, the universal healthcare model in the UK allows the development of a shared care plan involving primary care physicians and specialists. Since most childhood cancer patients survive well into adulthood, our results can help inform discussions with parents regarding therapy choice at the time of cancer diagnosis to weigh the benefits of a particular therapy with risks of possible late effects. Our findings demonstrate that the combination of chemotherapy and radiotherapy appreciably increased the burden of late effects – this trade-off between antitumour efficacy and late effects must be considered when designing front-line therapy. The National Comprehensive Cancer Network guidelines recommend that teenagers and young adults should be involved in decision-making with their parents and be provided with age-appropriate information.^52^ This is important because we show that mental health conditions are common late effects. Patient empowerment and psychological support at early stages are crucial for improving survivorship. Additionally, there are psychosocial effects associated with ongoing monitoring among survivors,^53^ thus, long-term individualised plans considering the holistic needs of each patient may be required to help them achieve the best possible quality of life.

## Data availability

The data used in this study are available on successful ethics application to the Clinical Practice Research Datalink (CPRD). All summarised data and results are made available as supplementary materials.

## Contributors

Research question: WHC and AGL

Funding: AGL

Study design and analysis plan: WHC and AGL

Preparation of data: WHC, SM and AGL

Statistical analysis: WHC and AGL

Statistical input: MK

Preparation of electronic health record code lists: YYT

Clinical interpretation: KG

Drafting initial and final versions of manuscript: WHC and AGL

Critical review of early and final versions of manuscript: All authors

All authors have directly accessed and verified the underlying data reported in the manuscript.

## Funding

AGL is supported by funding from the Wellcome Trust (204841/Z/16/Z), National Institute for Health Research (NIHR) University College London Hospitals Biomedical Research Centre (BRC714/HI/RW/101440), NIHR Great Ormond Street Hospital Biomedical Research Centre (19RX02), the Health Data Research UK Better Care Catalyst Award (CFC0125) and the Academy of Medical Sciences (SBF006\1084). The funders have no role in the writing of the manuscript or the decision to submit it for publication.

## Declaration of interests

None declared.

## Appendix Supplementary materials

Image, application 1

## Footnotes

Supplementary material associated with this article can be found in the online version at doi:10.1016/j.lanepe.2021.100248.
