# Supplementary material for: Late effects of cancer in children, teenagers and young adults: Population-based study on the burden of 183 conditions, in-patient and critical care admissions and years of life lost
Source: Lancet Reg Health Eur. 2021 Nov 14;12:100248. doi: 10.1016/j.lanepe.2021.100248 (PMC8672041; doi:10.1016/j.lanepe.2021.100248)
Supplement: Supplementary file 1 [file mmc1.pdf]

Supplementary file for

Late effects of cancer in children, teenagers and young adults: burden of 183 conditions, in-patient and critical care admissions and years of life lost

Wai Hoong Chang, Michail Katsoulis, Yen Yi Tan, Stefanie H. Mueller, Katherine Green, Alvina G. Lai

Supplementary information

Data analysis

Cumulative burden was estimated using the mean cumulative count (MCC) method[1,2]. Analysis scripts (R codes) for cumulative burden can be retrieved from this website (https://sites.ualberta.ca/~yyasui/software.html). Multivariable logistic regression analyses were performed according to the R codes from this website (https://www.r-bloggers.com/2021/04/logistic-regression-r-tutorial/). Excess years of life lost analyses were performed following the step-by-step instructions in a paper by Plana-Ripoll et al., 2020[3]. The following R packages were used: tidyverse, tableone, lillies, reshape, splines, survival, etm, mstate and cmprsk and user manuals can be downloaded from CRAN.

Open access electronic health record phenotypes for 183 conditions[4,5]

Step 1:

URLs for each condition can be retrieved from Table S2. For example, for abdominal aortic aneurysm, the URL is https://portal.caliberresearch.org/phenotypes/kuan-aaa-nj2gf6zttxjaymck5kshxf.

Step 2:
GP medical codes used to define each condition can be retrieved by clicking on the “Primary care” tab. Hospital codes can be retrieved from the “Secondary care” tab.

Step 3:
The “Implementation” tab contains the phenotyping algorithm used for each condition.

Step 4:
To download machine-readable csv files, click on the “Metadata” tab to retrieve code lists for primary and secondary care in Read vs and ICD-10, respectively.

References:

1 Dong H, Robison LL, Leisenring WM, Martin LJ, Armstrong GT, Yasui Y. Estimating the burden of recurrent events in the presence of competing risks: The method of mean cumulative count. *Am J Epidemiol* 2015; **181**: 532–40.

2 Bhakta N, Liu Q, Ness KK, *et al.* The cumulative burden of surviving childhood cancer: an initial report from the St Jude Lifetime Cohort Study (SJLIFE). *Lancet* 2017; **390**: 2569–82.

3 Plana-Ripoll O, Canudas-Romo V, Weye N, Laursen TM, McGrath JJ, Andersen PK. lillies: An R package for the estimation of excess Life Years Lost among patients with a given disease or condition. *PLoS One* 2020; **15**: e0228073.

4 Kuan V, Denaxas S, Gonzalez-Izquierdo A, *et al.* A chronological map of 308 physical and mental health conditions from 4 million individuals in the English National Health Service. *Lancet Digit Heal* 2019; **1**: e63–77.

5 Denaxas S, Gonzalez-Izquierdo A, Direk K, *et al.* UK phenomics platform for developing and validating electronic health record phenotypes: CALIBER. *J Am Med Informatics Assoc* 2019.

Figure S1. A map of each result to their corresponding dataset(s). CPRD: Clinical Practice Research Datalink; NCRAS (National Cancer Registration and Analysis Service); HES (Hospital Episode Statistics); APC (Admitted Patient Care); CC (Adult Critical Care); IMD (Index of Multiple Deprivation).

Figure 2 (Cumulative burden of health conditions)
Tables S3, S4, S5, S6, S7 and S8

- NCRAS (cancer diagnostic group, treatment type, chemotherapy type)
- CPRD GOLD (health conditions)
- CPRD Aurum (health conditions)
- HES APC (health conditions)
- IMD (deprivation status)

Figures 3A, 3C, 3D and 3E
Tables S9, S10, S11 and S12

- NCRAS (cancer diagnostic group, treatment type, chemotherapy type)
- HES APC (in-patient admissions)
- IMD (deprivation status)

Figure 3B
Table S9

- HES CC (critical care admissions)

Figure S2 (cumulative burden of 183 conditions)
Tables S16, S17, S18 and S19

- NCRAS (cancer diagnostic group, treatment type, chemotherapy type)
- CPRD GOLD (health conditions)
- CPRD Aurum (health conditions)
- HES APC (health conditions)
- IMD (deprivation status)

Figure 4 (multivariable regression of health outcomes)
Tables S13, S14 and S15

- NCRAS (treatment type, chemotherapy cumulative dose, radiation dose, teletherapy field)
- CPRD GOLD (health conditions)
- CPRD Aurum (health conditions)
- HES APC (health conditions)

Figure S3 (cumulative burden of infections and immunological conditions)
Table S20

- NCRAS (original cancer diagnosis codes)
- CPRD GOLD (health conditions and recurrent or subsequent cancers)
- CPRD Aurum (health conditions and recurrent or subsequent cancers)
- HES APC (health conditions and recurrent or subsequent cancers)

Figure 5 (excess years of life lost)
Table S21

- ONS (date of death)
- CPRD GOLD (health conditions)
- CPRD Aurum (health conditions)
- HES APC (health conditions)

Figure S2. Cumulative burden of 183 condition-specific outcomes for childhood cancer survivors and controls at age 45 years. Each tile in the heatmap corresponds to cumulative burden count per 100 persons for each condition-specific outcome and cohort strata. For example, a cumulative burden of 4.2 correlates to 4.2 events per 100 individuals. Condition-specific outcomes were rank ordered according to the cumulative burden in controls (first column on the left). Mean cumulative counts were separated into 10-quantiles (10 groups) resulting in quantile colour representation of the heatmaps. All data and 95% confidence intervals are provided in the supplementary tables.

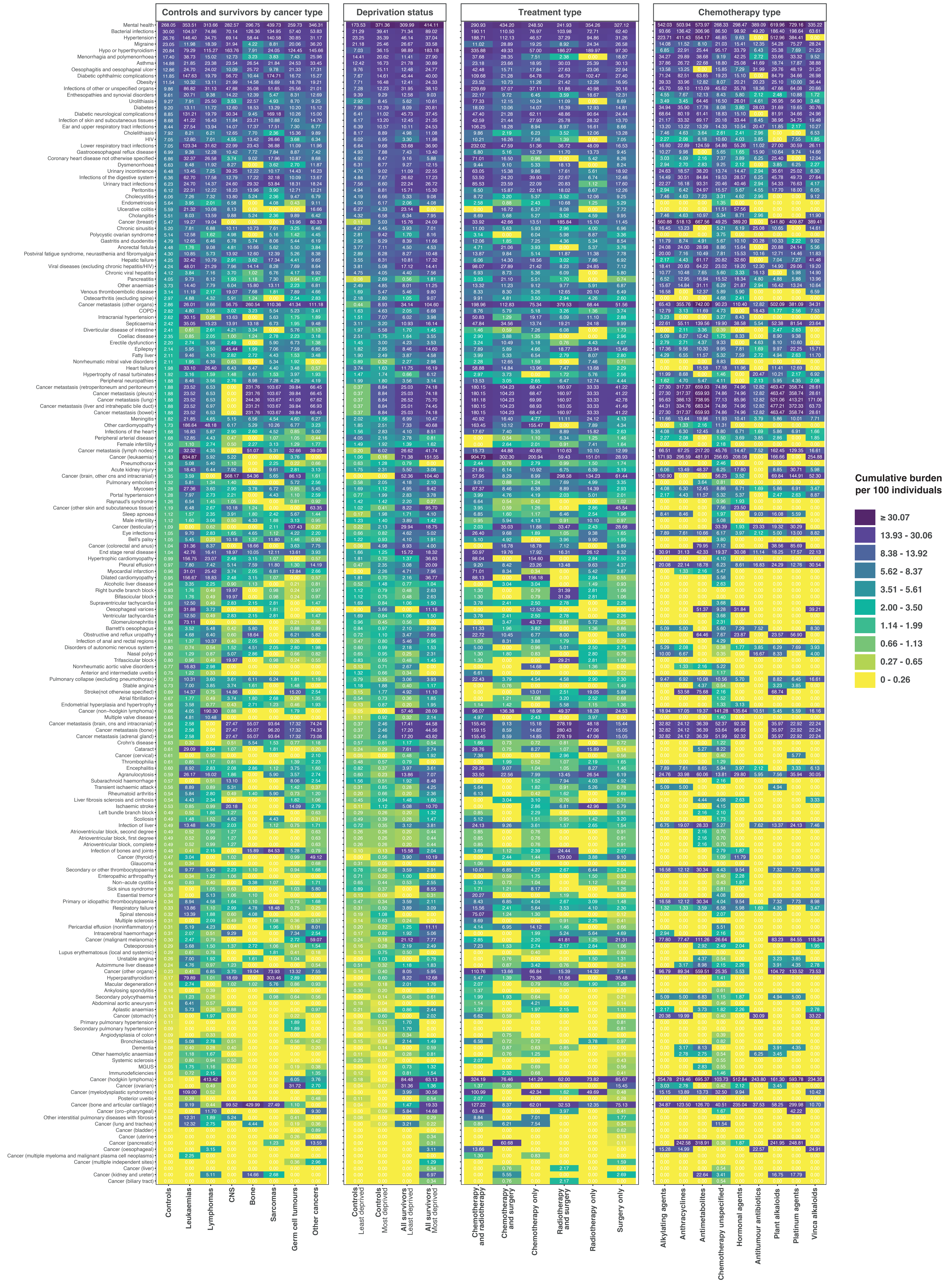

Figure S3. Cumulative burden of 25 infections and immunological conditions in cancer survivors. Patients were stratified into three groups: (i) patients who did not have any cancer events during the survivorship phase, (ii) patients with cancer recurrences and (iii) patients with subsequent (new) cancers.

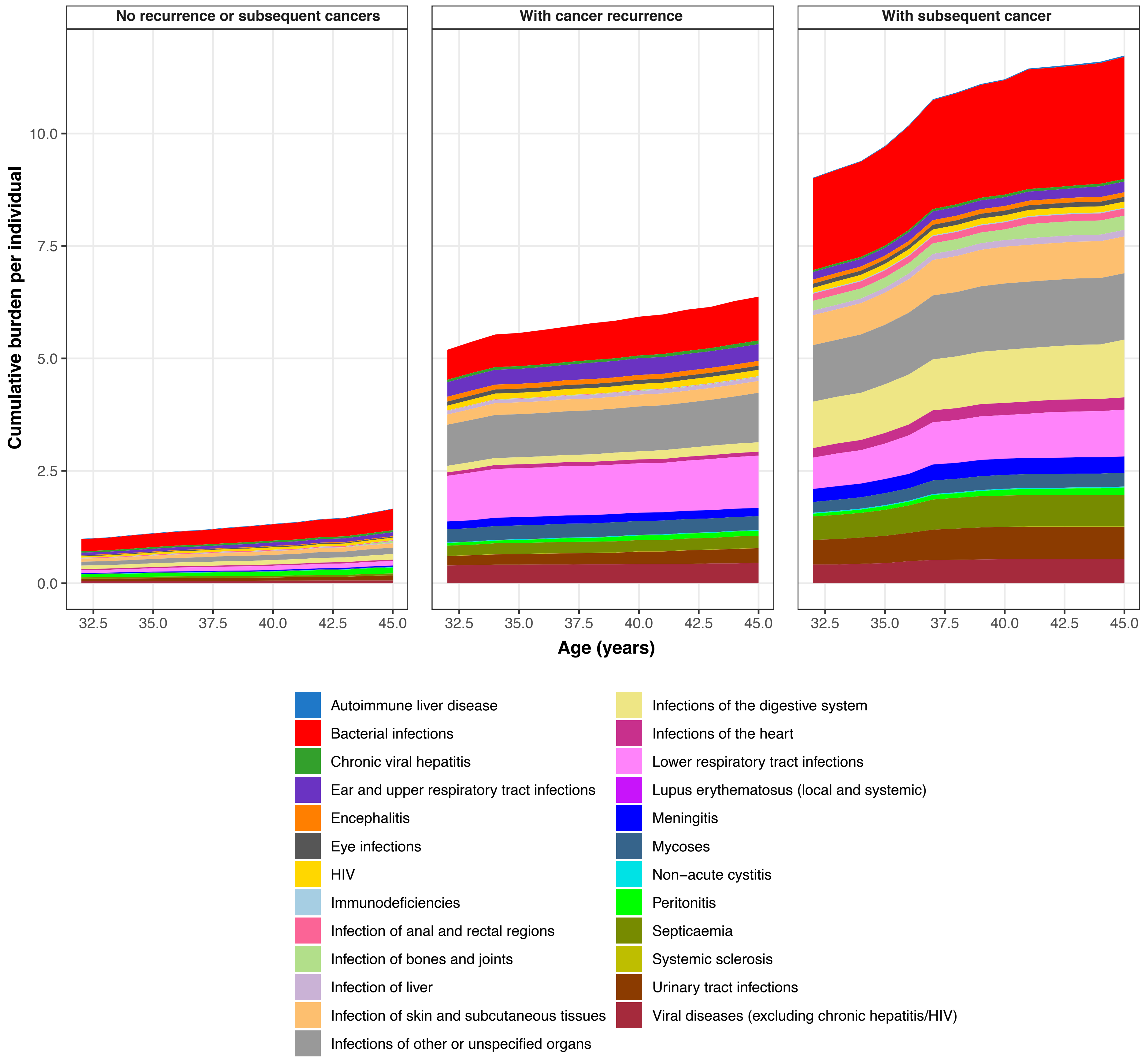

Table S1. Characteristics of the children, teenagers and young adults with cancer and community controls

	Total cancer population	Survivors	Controls
**n**	4063	3466	13517
**Male (%)**	2121 (52.2)	1782 (51.4)	7143 (52.8)
**Age cancer at diagnosis (%)**			
0-4 years	165 (4.1)	154 (4.4)	
5-9 years	264 (6.5)	203 (5.9)	
10-14 years	458 (11.3)	358 (10.3)	
15-19 years	921 (22.7)	750 (21.6)	
20-25 years	2255 (55.5)	2001 (57.7)	
**Indices of multiple deprivation (%)**			
Least deprived	855 (21.1)	732 (21.1)	2759 (20.4)
Most deprived	807 (19.9)	682 (19.7)	2854 (21.1)
**Primary cancer diagnosis (%)**			
Leukaemias, myeloproliferative diseases, and myelodysplastic diseases	542 (13.3)	403 (11.6)	
Lymphomas and reticuloendothelial neoplasms	688 (16.9)	615 (17.7)	
CNS and miscellaneous intracranial and intraspinal neoplasms	507 (12.5)	390 (11.3)	
Neuroblastoma and other peripheral nervous cell tumours	48 (1.2)	37 (1.1)	
Retinoblastoma	14 (0.3)	14 (0.4)	
Renal tumours	45 (1.1)	39 (1.1)	
Hepatic tumours	22 (0.5)	9 (0.3)	
Malignant bone tumours	221 (5.4)	144 (4.2)	
Soft tissue and other extraosseous sarcomas	207 (5.1)	160 (4.6)	
Germ cell tumours, trophoblastic tumours, and neoplasms of gonads	686 (16.9)	644 (18.6)	
Adrenocortical carcinomas	4 (0.1)	3 (0.1)	
Carcinomas of bladder	7 (0.2)	7 (0.2)	
Carcinomas of breast	58 (1.4)	51 (1.5)	
Carcinomas of cervix uteri	149 (3.7)	132 (3.8)	
Carcinomas of colon	92 (2.3)	77 (2.2)	
Carcinomas of salivary glands	28 (0.7)	28 (0.8)	
Malignant melanomas	420 (10.3)	395 (11.4)	
Nasopharyngeal carcinomas	14 (0.3)	10 (0.3)	
Skin carcinomas	179 (4.4)	178 (5.1)	
Thyroid carcinomas	132 (3.2)	130 (3.8)	
**Treatment type (%)**			
All chemotherapy	1444 (35.5)	1087 (31.4)	
All radiotherapy	774 (19.0)	542 (15.6)	
All surgery	2219 (54.6)	1998 (57.6)	
Chemotherapy only	697 (17.2)	559 (16.1)	
Radiotherapy only	182 (4.5)	132 (3.8)	
Surgery only	1505 (37.0)	1436 (41.4)	
Chemotherapy and radiotherapy	247 (6.1)	147 (4.2)	
Chemotherapy and surgery	369 (9.1)	299 (8.6)	
Radiotherapy and surgery	214 (5.3)	181 (5.2)	
**Chemotherapy type (%)**			
Alkylating agents	206 (5.1)	160 (4.6)	
Anthracyclines	194 (4.8)	156 (4.5)	
Antimetabolites	143 (3.5)	98 (2.8)	
Chemotherapy unspecified	577 (14.2)	427 (12.3)	
Hormonal agents (including corticosteroid hormones and sex hormones)	174 (4.3)	142 (4.1)	
Non-anthracycline antitumour antibiotics	138 (3.4)	120 (3.5)	
Plant alkaloids and natural products (excluding vinca alkaloids)	164 (4.0)	118 (3.4)	
Platinum agents	127 (3.1)	94 (2.7)	
Vinca alkaloids	173 (4.3)	141 (4.1)	

Table S2. Individual health conditions grouped by organ systems.

Health conditions	Organ system	URL of disease phenotype code lists	Event type
Abdominal aortic aneurysm	Cardiovascular	https://portal.caliberresearch.org/phenotypes/kuan-aaa-nj2gf6zttxjaymck5kshxf	Chronic/recurrent
Atrial fibrillation	Cardiovascular	https://portal.caliberresearch.org/phenotypes/kuan-af-7ld6qmwnjm3trhykwmpq6h	Chronic/recurrent
Atrioventricular block, complete	Cardiovascular	https://portal.caliberresearch.org/phenotypes/kuan-av-block-3-zrulcuexj8udmst9szs23v	Chronic/recurrent
Atrioventricular block, first degree	Cardiovascular	https://portal.caliberresearch.org/phenotypes/kuan-av-block-1-t9fgkkdrjmexkxnu9wjwpf	Chronic/recurrent
Atrioventricular block, second degree	Cardiovascular	https://portal.caliberresearch.org/phenotypes/kuan-av-block-2-ixpd4fbsvdwsrvwjravj59	Chronic/recurrent
Bifascicular block	Cardiovascular	https://portal.caliberresearch.org/phenotypes/kuan-bifasc-block-fezgkadcbmmujgysojnhkt	Chronic/recurrent
Coronary heart disease not otherwise specified	Cardiovascular	https://portal.caliberresearch.org/phenotypes/kuan-chd-nos-u66rc5otddejcbdzi2hj8k	Chronic/recurrent
Dilated cardiomyopathy	Cardiovascular	https://portal.caliberresearch.org/phenotypes/kuan-dcm-7qjvdgfapzcrl9pkjrzwjd	Chronic/recurrent
Heart failure	Cardiovascular	https://portal.caliberresearch.org/phenotypes/kuan-hf-yxinh8trwet5pog7g48clc	Chronic/recurrent
Hypertension	Cardiovascular	https://portal.caliberresearch.org/phenotypes/kuan-hypertension-inukpplkh7qtfxfaq8qjaa	Chronic/recurrent
Hypertrophic cardiomyopathy	Cardiovascular	https://portal.caliberresearch.org/phenotypes/kuan-hocm-hcttcgqz2cuoah5kgmdyg9	Chronic/recurrent
Intracerebral haemorrhage	Cardiovascular	https://portal.caliberresearch.org/phenotypes/kuan-intracereb-haem-msackfarzdcb5hqxtflz37	Single/recurrent
Ischaemic stroke	Cardiovascular	https://portal.caliberresearch.org/phenotypes/kuan-isch-stroke-nfrpsebdjbyxgwgzhqtq2a	Single/recurrent
Left bundle branch block	Cardiovascular	https://portal.caliberresearch.org/phenotypes/kuan-lbbb-nzhfqjlxsw3a8ovebtdpez	Chronic/recurrent
Multiple valve disease	Cardiovascular	https://portal.caliberresearch.org/phenotypes/kuan-mult-valve-53qjzqfvdltrpr8pao8ctz	Chronic/recurrent
Myocardial infarction	Cardiovascular	https://portal.caliberresearch.org/phenotypes/kuan-myocardial-infarction-lg5fydnaomhyfennkpx92j	Chronic/recurrent
Nonrheumatic aortic valve disorders	Cardiovascular	https://portal.caliberresearch.org/phenotypes/kuan-nonrh-aortic-6kcbwlsve8okuujcsogvmy	Chronic/recurrent
Nonrheumatic mitral valve disorders	Cardiovascular	https://portal.caliberresearch.org/phenotypes/kuan-nonrh-mitral-8pula8uoebjtzc4vfipejr	Chronic/recurrent
Other cardiomyopathy	Cardiovascular	https://portal.caliberresearch.org/phenotypes/kuan-cardiomyo-oth-l9vd69shmntpevacrtjdby	Chronic/recurrent
Pericardial effusion (noninflammatory)	Cardiovascular	https://portal.caliberresearch.org/phenotypes/kuan-pericardial-effusion-ddfgfs5bup29rz2ysjup9t	Chronic/recurrent
Peripheral arterial disease	Cardiovascular	https://portal.caliberresearch.org/phenotypes/kuan-peripheral-arterial-disease-44jczffvdpmniwqnrtmijx	Chronic/recurrent
Primary pulmonary hypertension	Cardiovascular	https://portal.caliberresearch.org/phenotypes/kuan-prim-pulm-htn-lxkwe9srvzuqspvnwo84e6	Chronic/not recurrent
Raynaud's syndrome	Cardiovascular	https://portal.caliberresearch.org/phenotypes/kuan-raynauds-kgdpso98yvdgbukhwltqom	Chronic/not recurrent
Right bundle branch block	Cardiovascular	https://portal.caliberresearch.org/phenotypes/kuan-rbbb-8ccg64eh9xay4azpdgss79	Chronic/recurrent
Secondary pulmonary hypertension	Cardiovascular	https://portal.caliberresearch.org/phenotypes/kuan-sec-pulm-htn-vcsg3gccqtnbmmd8kzghqh	Chronic/not recurrent
Sick sinus syndrome	Cardiovascular	https://portal.caliberresearch.org/phenotypes/kuan-sick-sinus-fkjrjhuhcfnpcysptjwdic	Chronic/recurrent
Stable angina	Cardiovascular	https://portal.caliberresearch.org/phenotypes/kuan-stable-angina-7amhmwhkjhbnc2w32de4rt	Chronic/recurrent
Stroke(not otherwise specified)	Cardiovascular	https://portal.caliberresearch.org/phenotypes/kuan-stroke-nos-xvns5oshwa9hrbkevj2yyl	Single/recurrent
Subarachnoid haemorrhage	Cardiovascular	https://portal.caliberresearch.org/phenotypes/kuan-subarach-vdhb96h287ty7fynxbtbeu	Single/recurrent
Supraventricular tachycardia	Cardiovascular	https://portal.caliberresearch.org/phenotypes/kuan-svt-ykhzwdurkdfezdvnjm9vge	Chronic/recurrent
Transient ischaemic attack	Cardiovascular	https://portal.caliberresearch.org/phenotypes/kuan-tia-xxlutcnthxos59d3ftn9fy	Single/recurrent
Trifascicular block	Cardiovascular	https://portal.caliberresearch.org/phenotypes/kuan-trifasc-block-9fo6p7phiao5lgmdgaw2ak	Chronic/recurrent
Unstable angina	Cardiovascular	https://portal.caliberresearch.org/phenotypes/kuan-unstable-angina-ccquofjx2zzprqrx8ubgdq	Chronic/recurrent
Venous thromboembolic disease	Cardiovascular	https://portal.caliberresearch.org/phenotypes/kuan-vte-ex-pe-jrttcdr2u5bo88gjap7hd6	Chronic/recurrent
Ventricular tachycardia	Cardiovascular	https://portal.caliberresearch.org/phenotypes/kuan-vt-mx78fjmjpjg5sxuzp9wyak	Chronic/recurrent
Diabetes	Endocrine	https://portal.caliberresearch.org/phenotypes/kuan-diabetes-bymfsbeu7tvb6ykrq8abvk	Chronic/not recurrent
Diabetic neurological complications	Endocrine	https://portal.caliberresearch.org/phenotypes/kuan-dm-neuro-tbatltjsibhrbremjqskuj	Chronic/recurrent
Diabetic ophthalmic complications	Endocrine	https://portal.caliberresearch.org/phenotypes/kuan-diab-eye-gkfgm8qsdkpabsxux6ted3	Chronic/recurrent
Hyperparathyroidism	Endocrine	https://portal.caliberresearch.org/phenotypes/kuan-pth-agwpxprudwmbcsvmqsjm6j	Chronic/recurrent
Hypo or hyperthyroidism	Endocrine	https://portal.caliberresearch.org/phenotypes/kuan-thyroid-fgvjoowzcgt2v9untydu5s	Chronic/recurrent
Obesity	Endocrine	https://portal.caliberresearch.org/phenotypes/kuan-obesity-gsbmfscz8omukiykmkkzcy	Chronic/not recurrent
Alcoholic liver disease	Gastrointestinal	https://portal.caliberresearch.org/phenotypes/kuan-liver-alc-dx2rrinjmddgrfjzejyuh7	Chronic/not recurrent
Angiodysplasia of colon	Gastrointestinal	https://portal.caliberresearch.org/phenotypes/kuan-angiodysplasia-colon-rsxyxn6u8dbdfnt8ctsbmp	Chronic/not recurrent
Anorectal fistula	Gastrointestinal	https://portal.caliberresearch.org/phenotypes/kuan-anorectal-fistula-jjfso6okvljadwf5szrl5a	Single/recurrent
Barrett's oesophagus	Gastrointestinal	https://portal.caliberresearch.org/phenotypes/kuan-barretts-9f7dztmqkkshuwqf4gttuu	Chronic/not recurrent
Cholangitis	Gastrointestinal	https://portal.caliberresearch.org/phenotypes/kuan-cholangitis-mnzdobbnj9ssehnw6ts2m7	Single/recurrent
Cholecystitis	Gastrointestinal	https://portal.caliberresearch.org/phenotypes/kuan-cholecystitis-zrmfqhcwmbbomircgsz6ph	Single/recurrent
Cholelithiasis	Gastrointestinal	https://portal.caliberresearch.org/phenotypes/kuan-cholelithiasis-3wylrcs4k4nqfmsfxfi5em	Single/recurrent
Coeliac disease	Gastrointestinal	https://portal.caliberresearch.org/phenotypes/kuan-coeliac-cqd3ig9yekavwvpyubnnhf	Chronic/not recurrent
Crohn's disease	Gastrointestinal	https://portal.caliberresearch.org/phenotypes/kuan-crohns-tdixvuef5scfmgkyalecad	Chronic/not recurrent
Diverticular disease of intestine	Gastrointestinal	https://portal.caliberresearch.org/phenotypes/kuan-diverticuli-omjowojmhqqbfmzfdhcufm	Chronic/recurrent
Fatty liver	Gastrointestinal	https://portal.caliberresearch.org/phenotypes/kuan-fatty-liver-maahaluemormfcis7gorgs	Chronic/not recurrent
Gastritis and duodenitis	Gastrointestinal	https://portal.caliberresearch.org/phenotypes/kuan-gastritis-duodenitis-a9rp5ymoeb7gbeyimopaj3	Chronic/not recurrent
Gastrooesophageal reflux disease	Gastrointestinal	https://portal.caliberresearch.org/phenotypes/kuan-gord-ugchhbqwypvdsdwpn2fupv	Chronic/not recurrent
Hepatic failure	Gastrointestinal	https://portal.caliberresearch.org/phenotypes/kuan-liver-fail-5f44cpa3onkpv3axcvvdcp	Chronic/recurrent
Liver fibrosis sclerosis and cirrhosis	Gastrointestinal	https://portal.caliberresearch.org/phenotypes/kuan-cirrhosis-2lw7p3ulbveqv3vynlryu2	Chronic/not recurrent
Oesophageal varices	Gastrointestinal	https://portal.caliberresearch.org/phenotypes/kuan-varices-ceaqpkkvsipa7enjez2zzy	Chronic/recurrent
Oesophagitis and oesophageal ulcer	Gastrointestinal	https://portal.caliberresearch.org/phenotypes/kuan-oesoph-ulc-d8zyipwzzber6k9wffwnvt	Chronic/recurrent
Pancreatitis	Gastrointestinal	https://portal.caliberresearch.org/phenotypes/kuan-pancreatitis-byv8xnqbj2sfhyyvsoudtt	Single/recurrent
Portal hypertension	Gastrointestinal	https://portal.caliberresearch.org/phenotypes/kuan-portal-htn-g8iqfgfjzbkrbwcyjgthst	Chronic/not recurrent
Ulcerative colitis	Gastrointestinal	https://portal.caliberresearch.org/phenotypes/kuan-ulc-colitis-kmm3csnoeie3mapccxjiqr	Chronic/recurrent
Agranulocytosis	Haematological	https://portal.caliberresearch.org/phenotypes/kuan-agranulocytosis-n6bbn4xqnikrvdaqpzeciv	Chronic/recurrent
Aplastic anaemias	Haematological	https://portal.caliberresearch.org/phenotypes/kuan-aplastic-gaqef827vem86l9hqmehbd	Chronic/not recurrent
MGUS	Haematological	https://portal.caliberresearch.org/phenotypes/kuan-mgus-dsdmauquzcq6bsrkduwrkj	Chronic/not recurrent
Other anaemias	Haematological	https://portal.caliberresearch.org/phenotypes/kuan-oth-anaemia-tkopqyysqmzwvkiwfahqnr	Chronic/not recurrent
Other haemolytic anaemias	Haematological	https://portal.caliberresearch.org/phenotypes/kuan-oth-haem-anaemia-6iakhravppnafrkvtvhsnz	Chronic/not recurrent
Primary or idiopathic thrombocytopaenia	Haematological	https://portal.caliberresearch.org/phenotypes/kuan-pri-thrombocytopaenia-cusrauexqqgbaehkvipawn	Chronic/not recurrent
Secondary or other thrombocytopaenia	Haematological	https://portal.caliberresearch.org/phenotypes/kuan-sec-oth-thrombocytopaenia-f5fumcav9ufcejkbz5jtg4	Chronic/not recurrent
Secondary polycythaemia	Haematological	https://portal.caliberresearch.org/phenotypes/kuan-2ry-polycythaemia-ipndxjmpau865m4dygyw3e	Chronic/not recurrent
Thrombophilia	Haematological	https://portal.caliberresearch.org/phenotypes/kuan-thrombophilia-vbqprrqmjzfgvhbrumkexz	Chronic/not recurrent
Autoimmune liver disease	Immunology and infection	https://portal.caliberresearch.org/phenotypes/kuan-autoimm-liver-ehcfqgupwewexfdz8vquby	Chronic/not recurrent
Bacterial infections	Immunology and infection	https://portal.caliberresearch.org/phenotypes/kuan-bacterial-ltt9ydm9ngbzkwqqy7xyif	Single/recurrent
Chronic viral hepatitis	Immunology and infection	https://portal.caliberresearch.org/phenotypes/kuan-chr-hep-ue8m4voqchggtrx2sqwfes	Chronic/not recurrent
Ear and upper respiratory tract infections	Immunology and infection	https://portal.caliberresearch.org/phenotypes/kuan-ear-urti-gtxr2v6qtbyiemfrchdvzr	Single/recurrent
Encephalitis	Immunology and infection	https://portal.caliberresearch.org/phenotypes/kuan-enceph-7zr2dcjp5bz6fgvwdqyriy	Single/recurrent
Eye infections	Immunology and infection	https://portal.caliberresearch.org/phenotypes/kuan-eye-mpmjwrtnbkfuhg57uf9ka8	Single/recurrent
HIV	Immunology and infection	https://portal.caliberresearch.org/phenotypes/kuan-hiv-5rrcq6il8uvpneahtffw9s	Chronic/not recurrent
Immunodeficiencies	Immunology and infection	https://portal.caliberresearch.org/phenotypes/kuan-immunodef-f6u5wxywxj4f6gftp34tpq	Chronic/not recurrent
Infection of anal and rectal regions	Immunology and infection	https://portal.caliberresearch.org/phenotypes/kuan-anorectal-dxgs3a67dafepmerczjjky	Single/recurrent
Infection of bones and joints	Immunology and infection	https://portal.caliberresearch.org/phenotypes/kuan-bone-nrmxhxhhmyizftm6huuaqv	Single/recurrent
Infection of liver	Immunology and infection	https://portal.caliberresearch.org/phenotypes/kuan-liver-9xcfyaevmf8xwwhlj3vj6q	Single/recurrent
Infection of skin and subcutaneous tissues	Immunology and infection	https://portal.caliberresearch.org/phenotypes/kuan-skin-6dje5dxscsy43vuhfopwkf	Single/recurrent
Infections of other or unspecified organs	Immunology and infection	https://portal.caliberresearch.org/phenotypes/kuan-oth-organs-7yqx9fgz3m2d6uckxu7b4q	Single/recurrent
Infections of the digestive system	Immunology and infection	https://portal.caliberresearch.org/phenotypes/kuan-digestive-vgfsivgzeidgyqexptxne6	Single/recurrent
Infections of the heart	Immunology and infection	https://portal.caliberresearch.org/phenotypes/kuan-heart-nfrqmcj5kt44ffgrbmk3ec	Single/recurrent
Lower respiratory tract infections	Immunology and infection	https://portal.caliberresearch.org/phenotypes/kuan-lrti-rddq59rszjqvlptmk5vecd	Single/recurrent
Lupus erythematosus (local and systemic)	Immunology and infection	https://portal.caliberresearch.org/phenotypes/kuan-sle-2zrkhclcifb2pt7za2mvng	Chronic/not recurrent
Meningitis	Immunology and infection	https://portal.caliberresearch.org/phenotypes/kuan-meningitis-n9xtfjtns9jjsvp4twuthj	Single/recurrent
Mycoses	Immunology and infection	https://portal.caliberresearch.org/phenotypes/kuan-mycoses-nssgbvtmfbbqjnve7ioave	Single/recurrent
Non-acute cystitis	Immunology and infection	https://portal.caliberresearch.org/phenotypes/kuan-chr-cystitis-6d7xsekk9pn2co6sqamdwp	Chronic/recurrent
Peritonitis	Immunology and infection	https://portal.caliberresearch.org/phenotypes/kuan-peritonitis-7y4ue6sqxc3unfzpspk6ra	Single/recurrent
Septicaemia	Immunology and infection	https://portal.caliberresearch.org/phenotypes/kuan-sepsis-9kmibxtwkrhhrpxgvygopt	Single/recurrent
Systemic sclerosis	Immunology and infection	https://portal.caliberresearch.org/phenotypes/kuan-sys-sclerosis-lcpudgram6fmc3hzba8ukb	Chronic/not recurrent
Urinary tract infections	Immunology and infection	https://portal.caliberresearch.org/phenotypes/kuan-uti-hsazeuuryufgkk8dhavlks	Single/recurrent
Viral diseases (excluding chronic hepatitis/HIV)	Immunology and infection	https://portal.caliberresearch.org/phenotypes/kuan-viral-yatjlaqkxs2amocxf3yarb	Single/recurrent
Mental health	Mental health	https://portal.caliberresearch.org/phenotypes/carr-anxiety-5bqxgmaqvzfbhteyb5mhzj<br>https://portal.caliberresearch.org/phenotypes/kuan-depression-ceacxgefhpdvwcugwulatv	Chronic/recurrent
Ankylosing spondylitis	Musculoskeletal	https://portal.caliberresearch.org/phenotypes/kuan-ank-spond-m52wcz3izzztqd7pswheb5	Chronic/not recurrent
Enteropathic arthropathy	Musculoskeletal	https://portal.caliberresearch.org/phenotypes/kuan-entero-arthro-pmpjhfpnawsr8x96ns3vfb	Chronic/not recurrent
Enthesopathies and synovial disorders	Musculoskeletal	https://portal.caliberresearch.org/phenotypes/kuan-enthesopathy-95zhqe7diurqwsqrn8urmt	Chronic/not recurrent
Osteoarthritis (excluding spine)	Musculoskeletal	https://portal.caliberresearch.org/phenotypes/kuan-oa-mymazgrsrw7cghwd4di64e	Chronic/not recurrent
Osteoporosis	Musculoskeletal	https://portal.caliberresearch.org/phenotypes/kuan-osteoporosis-wp8ckuqqpyvjznrhp27hgg	Chronic/not recurrent
Rheumatoid arthritis	Musculoskeletal	https://portal.caliberresearch.org/phenotypes/kuan-rha-ewvrla7dnmcigs2xvc2zkt	Chronic/not recurrent
Scoliosis	Musculoskeletal	https://portal.caliberresearch.org/phenotypes/kuan-scoliosis-gwkkqmfddtierfqt3gdbgu	Chronic/not recurrent
Spinal stenosis	Musculoskeletal	https://portal.caliberresearch.org/phenotypes/kuan-spinal-stenosis-yhvfzs8btigrv4mn3fnvcd	Chronic/recurrent
Cancer (biliary tract)	Neoplasm	https://portal.caliberresearch.org/phenotypes/kuan-pri-biliary-kjbfd3byfff5f6dn4cz7zy	Single/recurrent
Cancer (bladder)	Neoplasm	https://portal.caliberresearch.org/phenotypes/kuan-pri-bladder-4eykyg9kzvvaej8ef3itgd	Single/recurrent
Cancer (bone and articular cartilage)	Neoplasm	https://portal.caliberresearch.org/phenotypes/kuan-pri-bone-qljnykgxentawop8gl7lhd	Single/recurrent
Cancer (brain, other cns and intracranial)	Neoplasm	https://portal.caliberresearch.org/phenotypes/kuan-pri-brain-mgshvmncfqkgdg6e4giqyx	Single/recurrent
Cancer (breast)	Neoplasm	https://portal.caliberresearch.org/phenotypes/kuan-pri-breast-fksmkhedz2v5aefuoxzzs2	Single/recurrent
Cancer (cervical)	Neoplasm	https://portal.caliberresearch.org/phenotypes/kuan-pri-cervical-ff27tqu3myusrfx4lglvnb	Single/recurrent
Cancer (colorectal and anus)	Neoplasm	https://portal.caliberresearch.org/phenotypes/kuan-pri-bowel-5fw4nu5bbuffnmwjadcet3	Single/recurrent
Cancer (hodgkin lymphoma)	Neoplasm	https://portal.caliberresearch.org/phenotypes/kuan-hodgkins-aax3frk37rsvpwiunu27on	Single/recurrent
Cancer (kidney and ureter)	Neoplasm	https://portal.caliberresearch.org/phenotypes/kuan-pri-kidney-w5cc5g9hzuekususnyphf4	Single/recurrent
Cancer (leukaemia)	Neoplasm	https://portal.caliberresearch.org/phenotypes/kuan-leukaemia-m4hfbxczafvwbbmj6h3wv8	Single/recurrent
Cancer (liver)	Neoplasm	https://portal.caliberresearch.org/phenotypes/kuan-pri-liver-aypekghxacccjejhmhgacf	Single/recurrent
Cancer (lung and trachea)	Neoplasm	https://portal.caliberresearch.org/phenotypes/kuan-pri-lung-amupxu3c33bfegnj3ljian	Single/recurrent
Cancer (malignant melanoma)	Neoplasm	https://portal.caliberresearch.org/phenotypes/kuan-pri-melanoma-wxanzjrixf3bntaug4snzc	Single/recurrent
Cancer (multiple independent sites)	Neoplasm	https://portal.caliberresearch.org/phenotypes/kuan-pri-multindep-en6gobmprwkohdigcp3bpz	Single/recurrent
Cancer (multiple myeloma and malignant plasma cell neoplasms)	Neoplasm	https://portal.caliberresearch.org/phenotypes/kuan-plasmacell-arsqvavguw2yqnz7bvcff4	Single/recurrent
Cancer (myelodysplastic syndromes)	Neoplasm	https://portal.caliberresearch.org/phenotypes/kuan-mds-aydke8fpddcymgoyarhuux	Single/recurrent
Cancer (non-hodgkin lymphoma)	Neoplasm	https://portal.caliberresearch.org/phenotypes/kuan-nhl-ekurllmzvbdtbfhqs3rnlr	Single/recurrent
Cancer (oesophageal)	Neoplasm	https://portal.caliberresearch.org/phenotypes/kuan-pri-oesoph-lks5brkv7vwh2j8yrdqsgn	Single/recurrent
Cancer (oro-pharyngeal)	Neoplasm	https://portal.caliberresearch.org/phenotypes/kuan-pri-oroph-yf7wejabxcmh9ahrkge8oa	Single/recurrent
Cancer (other organs)	Neoplasm	https://portal.caliberresearch.org/phenotypes/kuan-pri-other-5oehmm6bfgj5twvqwpso7s	Single/recurrent
Cancer (other skin and subcutaneous tissue)	Neoplasm	https://portal.caliberresearch.org/phenotypes/kuan-pri-skin-27goizyiiu2g8ef4qutuch	Single/recurrent
Cancer (ovarian)	Neoplasm	https://portal.caliberresearch.org/phenotypes/kuan-pri-ovarian-cyy4hsptczquaslxgdynqy	Single/recurrent
Cancer (pancreatic)	Neoplasm	https://portal.caliberresearch.org/phenotypes/kuan-pri-pancr-3gl6q35vvkcwycz2f4ymgz	Single/recurrent
Cancer (stomach)	Neoplasm	https://portal.caliberresearch.org/phenotypes/kuan-pri-stomach-nwxtekww9ppu4grcernrsp	Single/recurrent
Cancer (testicular)	Neoplasm	https://portal.caliberresearch.org/phenotypes/kuan-pri-testis-tvfnwmvxw5fpf6nasm7kq3	Single/recurrent
Cancer (thyroid)	Neoplasm	https://portal.caliberresearch.org/phenotypes/kuan-pri-thyroid-zt3pukfh9kc4i8hjwjcgjt	Single/recurrent
Cancer (uterine	Neoplasm	https://portal.caliberresearch.org/phenotypes/kuan-pri-uterine-pa3kyo4onvf8fxgmehtocm	Single/recurrent
Cancer metastasis (adrenal gland)	Neoplasm (metastasis)	https://portal.caliberresearch.org/phenotypes/kuan-sec-adrenal-mwchqysc5loatrfwxmwbb9	Single/recurrent
Cancer metastasis (bone)	Neoplasm (metastasis)	https://portal.caliberresearch.org/phenotypes/kuan-sec-bone-fhthhcsart7rxbax6a5t2k	Single/recurrent
Cancer metastasis (bowel)	Neoplasm (metastasis)	https://portal.caliberresearch.org/phenotypes/kuan-sec-bowel-hdwrfrcdeivxstswbi8kgp	Single/recurrent
Cancer metastasis (brain, cns and intracranial)	Neoplasm (metastasis)	https://portal.caliberresearch.org/phenotypes/kuan-sec-brain-7e8wg49zqwegfbun6bmhxq	Single/recurrent
Cancer metastasis (liver and intrahepatic bile duct)	Neoplasm (metastasis)	https://portal.caliberresearch.org/phenotypes/kuan-sec-liver-mlbtbvxelaknfsyiafybaa	Single/recurrent
Cancer metastasis (lung)	Neoplasm (metastasis)	https://portal.caliberresearch.org/phenotypes/kuan-sec-lung-m7bkxta8pkcftedabweoqr	Single/recurrent
Cancer metastasis (lymph nodes)	Neoplasm (metastasis)	https://portal.caliberresearch.org/phenotypes/kuan-sec-ln-kxepdmhgmskrrxzvwt3gcy	Single/recurrent
Cancer metastasis (other organs)	Neoplasm (metastasis)	https://portal.caliberresearch.org/phenotypes/kuan-sec-other-dcdeyhfps3kct6qfrgvnsx	Single/recurrent
Cancer metastasis (pleura)	Neoplasm (metastasis)	https://portal.caliberresearch.org/phenotypes/kuan-sec-pleura-dzjsetbylqpel4sjvuykh7	Single/recurrent
Cancer metastasis (retroperitoneum and peritoneum	Neoplasm (metastasis)	https://portal.caliberresearch.org/phenotypes/kuan-sec-peritoneum-3eh9str5jceazqysbdkvss	Single/recurrent
Bell's palsy	Neurological	https://portal.caliberresearch.org/phenotypes/kuan-bells-zgv8ujfkg87ayavhuvvdwz	Single/recurrent
Dementia	Neurological	https://portal.caliberresearch.org/phenotypes/kuan-dementia-jxyxcgdmoqd58igunbzatr	Chronic/not recurrent
Disorders of autonomic nervous system	Neurological	https://portal.caliberresearch.org/phenotypes/kuan-autonomic-neuro-lfgljazsu9dqcewvusohxm	Single/recurrent
Epilepsy	Neurological	https://portal.caliberresearch.org/phenotypes/kuan-epilepsy-wg2um5mf2unhq57ya44gzl	Chronic/not recurrent
Essential tremor	Neurological	https://portal.caliberresearch.org/phenotypes/kuan-essential-tremor-6kbzkdiuqgqkuujynuyrba	Chronic/recurrent
Intracranial hypertension	Neurological	https://portal.caliberresearch.org/phenotypes/kuan-intracranial-htn-giqnx539aerzlwmixxd2sk	Chronic/recurrent
Migraine	Neurological	https://portal.caliberresearch.org/phenotypes/kuan-migraine-burbye64pvgcarg8ik4aqk	Chronic/recurrent
Multiple sclerosis	Neurological	https://portal.caliberresearch.org/phenotypes/kuan-ms-o3phknpsjohi4ewuwrqknh	Chronic/not recurrent
Peripheral neuropathies	Neurological	https://portal.caliberresearch.org/phenotypes/kuan-periph-neuro-ezrssl9ryypafk67puln56	Chronic/not recurrent
Postviral fatigue syndrome, neurasthenia and fibromyalgia	Neurological	https://portal.caliberresearch.org/phenotypes/kuan-chronic-fatigue-4vuyvqjem58jemuvwovnwx	Chronic/not recurrent
Anterior and intermediate uveitis	Ocular	https://portal.caliberresearch.org/phenotypes/kuan-ant-uveitis-kzw9feefguyyymihmhgeew	Single/recurrent
Cataract	Ocular	https://portal.caliberresearch.org/phenotypes/kuan-cataract-yfnhfx9tgynqzhbcryxvvq	Chronic/recurrent
Glaucoma	Ocular	https://portal.caliberresearch.org/phenotypes/kuan-glaucoma-9vfceuu9tbzji8yhf4u7hh	Chronic/recurrent
Macular degeneration	Ocular	https://portal.caliberresearch.org/phenotypes/kuan-macula-degen-2v58jy9jvmvvvokaa3e5sb	Chronic/not recurrent
Posterior uveitis	Ocular	https://portal.caliberresearch.org/phenotypes/kuan-post-uveitis-7st585s2mhmvcxr3qxn4vh	Single/recurrent
Chronic sinusitis	Otorhinolaryngological	https://portal.caliberresearch.org/phenotypes/kuan-sinusitis-yxtns5supzvfxavs3rdksm	Chronic/not recurrent
Hypertrophy of nasal turbinates	Otorhinolaryngological	https://portal.caliberresearch.org/phenotypes/kuan-hyper-nasal-turbs-w83wmb5a6k6mfqkujjzt6e	Chronic/not recurrent
Nasal polyp	Otorhinolaryngological	https://portal.caliberresearch.org/phenotypes/kuan-nasal-polyp-2jqmo7mxn89urpqarnrvau	Chronic/not recurrent
Sleep apnoea	Otorhinolaryngological	https://portal.caliberresearch.org/phenotypes/kuan-sleep-apnoea-afzq2jdrkumsy8qtbserxq	Chronic/not recurrent
Asthma	Pulmonary	https://portal.caliberresearch.org/phenotypes/kuan-asthma-efthigq2rjygabhhuco4pw	Chronic/recurrent
Bronchiectasis	Pulmonary	https://portal.caliberresearch.org/phenotypes/kuan-bronchiectasis-swuahahstc967nlcfhy6rc	Chronic/not recurrent
COPD	Pulmonary	https://portal.caliberresearch.org/phenotypes/kuan-copd-kgwojwpdpg8npp8huc37zd	Chronic/recurrent
Other interstitial pulmonary diseases with fibrosis	Pulmonary	https://portal.caliberresearch.org/phenotypes/kuan-pulm-fibrosis-hro7ob4dxzxydjqocqyp2b	Chronic/recurrent
Pleural effusion	Pulmonary	https://portal.caliberresearch.org/phenotypes/kuan-pleural-effusion-bqbxwrnday7n7fvalyjxbd	Single/recurrent
Pneumothorax	Pulmonary	https://portal.caliberresearch.org/phenotypes/kuan-pneumothorax-a5nnubaklzydsqiw9ujpbb	Single/recurrent
Pulmonary collapse (excluding pneumothorax)	Pulmonary	https://portal.caliberresearch.org/phenotypes/kuan-pulm-collapse-rsyq6aacuxgqo5s3xhfwtn	Single/recurrent
Pulmonary embolism	Pulmonary	https://portal.caliberresearch.org/phenotypes/kuan-pe-b82p672dxcbwfyeqp9fjjh	Single/recurrent
Respiratory failure	Pulmonary	https://portal.caliberresearch.org/phenotypes/kuan-resp-failure-fucduez9x5u3qklysmrxux	Single/recurrent
Acute kidney injury	Renal	https://portal.caliberresearch.org/phenotypes/kuan-aki-nrvedg2xdppjt2odqcadfg	Single/recurrent
End stage renal disease	Renal	https://portal.caliberresearch.org/phenotypes/kuan-esrd-656rd4trga4b7ghmajzazt	Chronic/not recurrent
Glomerulonephritis	Renal	https://portal.caliberresearch.org/phenotypes/kuan-gn-2wtwqbk6ypwjowgxfj3x3p	Single/recurrent
Obstructive and reflux uropathy	Renal	https://portal.caliberresearch.org/phenotypes/kuan-obstr-reflux-lpqmvkgmacace2w5zsxotg	Chronic/recurrent
Urinary incontinence	Renal	https://portal.caliberresearch.org/phenotypes/kuan-urine-incont-yvvpldfgeuawaqghcgedrt	Chronic/recurrent
Urolithiasis	Renal	https://portal.caliberresearch.org/phenotypes/kuan-urolithiasis-zsx6gftukjrfjvraurjeeu	Single/recurrent
Dysmenorrhoea	Reproductive	https://portal.caliberresearch.org/phenotypes/kuan-dysmenorrhoea-ypx3hmzkey6ye8h7fura54	Chronic/recurrent
Endometrial hyperplasia and hypertrophy	Reproductive	https://portal.caliberresearch.org/phenotypes/kuan-endometrial-hyper-x877b8y2f3gjmtxhye5q4j	Chronic/recurrent
Endometriosis	Reproductive	https://portal.caliberresearch.org/phenotypes/kuan-endometriosis-2egsy3aq93sdxxakc4jlwf	Chronic/recurrent
Erectile dysfunction	Reproductive	https://portal.caliberresearch.org/phenotypes/kuan-ed-k5jqbm97nneehgdbanj35x	Chronic/not recurrent
Female infertility	Reproductive	https://portal.caliberresearch.org/phenotypes/kuan-female-infertility-5uvfsyinhtbyqle8wqmfue	Chronic/not recurrent
Male infertility	Reproductive	https://portal.caliberresearch.org/phenotypes/kuan-male-infertility-atmsyqkqnjabzawpds5chv	Chronic/not recurrent
Menorrhagia and polymenorrhoea	Reproductive	https://portal.caliberresearch.org/phenotypes/kuan-menorrhagia-5gg3vvf9jqgze7kxzwe577	Chronic/recurrent
Polycystic ovarian syndrome	Reproductive	https://portal.caliberresearch.org/phenotypes/kuan-pcos-gkcvzqrcqjhkwtvynemynz	Chronic/recurrent

Table S3. Overall cumulative burden for health conditions for all survivors and controls at 35, 40 and 45 years attained age. Cumulative burden per individual is shown.

	35 years			40 years			45 years		
**Cancer diagnostic groups**	**Cumulative Burden**	**Lower CI**	**Upper CI**	**Cumulative Burden**	**Lower CI**	**Upper CI**	**Cumulative Burden**	**Lower CI**	**Upper CI**
Leukaemias, myeloproliferative diseases, and myelodysplastic diseases	23.52	19.85	29.33	26.78	22.31	34.18	29.79	24.66	35.95
Lymphomas and reticuloendothelial neoplasms	7.78	7.11	9.04	10.40	8.91	11.77	13.38	11.62	15.56
CNS and miscellaneous intracranial and intraspinal neoplasms	9.68	8.81	10.96	11.69	11.23	12.39	13.80	13.24	14.71
Malignant bone tumours	6.61	5.68	6.92	9.85	8.39	10.77	10.30	8.94	11.30
Soft tissue and other extraosseous sarcomas	9.57	6.44	10.74	11.16	7.35	12.58	20.54	7.90	26.91
Germ cell tumours, trophoblastic tumours, and neoplasms of gonads	6.04	5.32	6.91	7.39	6.32	8.45	9.07	7.96	9.91
Other cancers	7.29	6.31	8.30	9.43	8.17	10.67	11.13	9.92	12.64
	**Cumulative Burden**	**Lower CI**	**Upper CI**	**Cumulative Burden**	**Lower CI**	**Upper CI**	**Cumulative Burden**	**Lower CI**	**Upper CI**
**Controls**	3.99	3.93	4.08	5.36	5.32	5.46	7.19	7.10	7.36

Table S4. Cumulative burden for conditions grouped by organ systems for all survivors and controls at 35, 40 and 45 years attained age. Cumulative burden per individual is shown.

	35 years			40 years			45 years			
**Health conditions by organ system**	**Cumulative Burden**	**Lower CI**	**Upper CI**	**Cumulative Burden**	**Lower CI**	**Upper CI**	**Cumulative Burden**	**Lower CI**	**Upper CI**	**Group**
Cardiovascular	2.25	1.11	2.41	2.59	1.46	2.76	3.08	1.98	3.29	All survivors
Endocrine	1.91	1.42	2.17	2.18	1.67	2.35	2.59	2.06	2.85	All survivors
Gastrointestinal	0.71	0.67	0.89	0.92	0.87	1.10	1.14	1.06	1.29	All survivors
Haematological	0.25	0.24	0.27	0.28	0.27	0.31	0.30	0.28	0.33	All survivors
Immunology and infection	2.52	2.14	2.98	2.90	2.59	3.34	3.27	3.01	3.58	All survivors
Mental health	2.02	1.79	2.12	2.68	2.45	2.83	3.19	2.95	3.40	All survivors
Musculoskeletal, Ocular & Otorhinolaryngological	0.31	0.26	0.36	0.37	0.32	0.42	0.45	0.39	0.50	All survivors
Neurological	0.55	0.47	0.63	0.63	0.54	0.72	0.69	0.61	0.77	All survivors
Pulmonary	0.44	0.43	0.52	0.49	0.47	0.58	0.54	0.52	0.62	All survivors
Renal	0.49	0.35	0.59	0.57	0.42	0.66	0.64	0.50	0.72	All survivors
Reproductive	0.28	0.27	0.30	0.37	0.34	0.40	0.44	0.41	0.51	All survivors
**Health conditions by organ system**	**Cumulative Burden**	**Lower CI**	**Upper CI**	**Cumulative Burden**	**Lower CI**	**Upper CI**	**Cumulative Burden**	**Lower CI**	**Upper CI**	**Group**
Cardiovascular	0.19	0.17	0.22	0.31	0.28	0.34	0.61	0.54	0.67	Controls
Endocrine	0.29	0.28	0.31	0.43	0.41	0.45	0.62	0.59	0.67	Controls
Gastrointestinal	0.35	0.34	0.35	0.51	0.49	0.54	0.70	0.64	0.76	Controls
Haematological	0.04	0.04	0.04	0.05	0.05	0.05	0.06	0.06	0.07	Controls
Immunology and infection	0.68	0.67	0.71	0.88	0.87	0.91	1.12	1.06	1.17	Controls
Mental health	1.56	1.51	1.72	2.04	1.92	2.20	2.68	2.46	2.87	Controls
Musculoskeletal, Ocular & Otorhinolaryngological	0.15	0.15	0.15	0.20	0.19	0.21	0.26	0.24	0.27	Controls
Neurological	0.23	0.21	0.25	0.30	0.27	0.32	0.37	0.35	0.38	Controls
Pulmonary	0.18	0.17	0.18	0.20	0.19	0.21	0.23	0.21	0.23	Controls
Renal	0.08	0.08	0.09	0.15	0.12	0.20	0.20	0.16	0.25	Controls
Reproductive	0.25	0.24	0.26	0.31	0.30	0.33	0.40	0.37	0.41	Controls

Table S5. Cumulative burden for conditions grouped by organ systems and indices of multiple deprivation (IMD) for all survivors and controls at 35, 40 and 45 years attained age. Cumulative burden per individual is shown.

	35 years			40 years			45 years				
**Health conditions by organ system**	**Cumulative Burden**	**Lower CI**	**Upper CI**	**Cumulative Burden**	**Lower CI**	**Upper CI**	**Cumulative Burden**	**Lower CI**	**Upper CI**	**IMD strata**	**Group**
Cardiovascular	0.47	0.35	0.59	0.68	0.47	0.87	1.16	0.99	1.53	IMD 1	All survivors
Endocrine	1.25	1.07	1.48	1.33	1.16	1.58	2.14	1.87	3.22	IMD 1	All survivors
Gastrointestinal	0.37	0.29	0.37	0.68	0.54	0.73	0.98	0.80	1.01	IMD 1	All survivors
Haematological	0.25	0.17	0.36	0.29	0.21	0.40	0.32	0.24	0.42	IMD 1	All survivors
Immunology and infection	1.73	1.42	1.87	2.22	1.92	2.22	2.97	2.21	3.83	IMD 1	All survivors
Mental health	1.83	1.38	1.92	2.56	1.85	2.72	3.10	2.31	3.34	IMD 1	All survivors
Musculoskeletal, Ocular & Otorhinolaryngological	0.22	0.17	0.26	0.25	0.20	0.28	0.33	0.30	0.36	IMD 1	All survivors
Neurological	0.45	0.37	0.50	0.50	0.41	0.53	0.60	0.50	0.61	IMD 1	All survivors
Pulmonary	0.31	0.23	0.36	0.35	0.28	0.39	0.40	0.37	0.44	IMD 1	All survivors
Renal	0.25	0.21	0.31	0.35	0.29	0.50	0.41	0.33	0.54	IMD 1	All survivors
Reproductive	0.23	0.18	0.26	0.30	0.24	0.32	0.33	0.28	0.35	IMD 1	All survivors
Cardiovascular	1.63	1.06	2.11	1.92	1.19	2.39	2.55	1.77	4.01	IMD 5	All survivors
Endocrine	2.03	1.43	2.57	2.41	1.70	2.87	3.04	2.03	3.89	IMD 5	All survivors
Gastrointestinal	0.87	0.50	1.22	0.97	0.60	1.34	1.24	0.83	1.48	IMD 5	All survivors
Haematological	0.26	0.21	0.34	0.28	0.24	0.36	0.28	0.24	0.36	IMD 5	All survivors
Immunology and infection	2.67	2.13	3.28	3.19	2.64	3.61	3.47	2.97	3.97	IMD 5	All survivors
Mental health	2.27	1.94	2.48	3.30	2.88	3.60	4.14	3.62	4.34	IMD 5	All survivors
Musculoskeletal, Ocular & Otorhinolaryngological	0.26	0.23	0.28	0.34	0.28	0.36	0.46	0.38	0.52	IMD 5	All survivors
Neurological	0.61	0.51	0.67	0.72	0.57	0.77	0.81	0.65	0.89	IMD 5	All survivors
Pulmonary	0.57	0.41	0.61	0.62	0.44	0.66	0.67	0.49	0.72	IMD 5	All survivors
Renal	0.50	0.33	0.53	0.57	0.42	0.56	0.61	0.46	0.60	IMD 5	All survivors
Reproductive	0.42	0.30	0.50	0.49	0.34	0.60	0.59	0.45	0.67	IMD 5	All survivors
**Health conditions by organ system**	**Cumulative Burden**	**Lower CI**	**Upper CI**	**Cumulative Burden**	**Lower CI**	**Upper CI**	**Cumulative Burden**	**Lower CI**	**Upper CI**	**IMD strata**	**Group**
Cardiovascular	0.16	0.12	0.16	0.23	0.18	0.26	0.57	0.47	0.65	IMD 1	Controls
Endocrine	0.16	0.12	0.17	0.25	0.21	0.28	0.37	0.30	0.41	IMD 1	Controls
Gastrointestinal	0.27	0.23	0.27	0.42	0.36	0.47	0.57	0.50	0.64	IMD 1	Controls
Haematological	0.03	0.02	0.03	0.05	0.04	0.05	0.05	0.04	0.06	IMD 1	Controls
Immunology and infection	0.55	0.50	0.59	0.73	0.66	0.77	0.91	0.79	1.00	IMD 1	Controls
Mental health	1.07	0.90	1.12	1.40	1.18	1.54	1.74	1.40	2.02	IMD 1	Controls
Musculoskeletal, Ocular & Otorhinolaryngological	0.13	0.12	0.13	0.19	0.17	0.18	0.22	0.20	0.22	IMD 1	Controls
Neurological	0.22	0.19	0.24	0.26	0.21	0.28	0.33	0.27	0.34	IMD 1	Controls
Pulmonary	0.14	0.12	0.14	0.16	0.14	0.17	0.17	0.16	0.18	IMD 1	Controls
Renal	0.07	0.06	0.09	0.10	0.08	0.12	0.13	0.09	0.16	IMD 1	Controls
Reproductive	0.21	0.19	0.24	0.27	0.24	0.29	0.32	0.29	0.35	IMD 1	Controls
Cardiovascular	0.24	0.20	0.28	0.42	0.32	0.48	0.76	0.55	0.85	IMD 5	Controls
Endocrine	0.44	0.32	0.54	0.63	0.50	0.77	0.91	0.64	1.18	IMD 5	Controls
Gastrointestinal	0.42	0.37	0.43	0.63	0.57	0.70	0.90	0.75	0.95	IMD 5	Controls
Haematological	0.05	0.04	0.05	0.05	0.05	0.06	0.07	0.05	0.07	IMD 5	Controls
Immunology and infection	0.81	0.71	0.87	1.08	0.95	1.17	1.36	1.21	1.51	IMD 5	Controls
Mental health	2.10	1.83	2.18	2.75	2.37	2.97	3.71	3.20	4.12	IMD 5	Controls
Musculoskeletal, Ocular & Otorhinolaryngological	0.16	0.15	0.16	0.20	0.18	0.20	0.25	0.23	0.27	IMD 5	Controls
Neurological	0.25	0.23	0.28	0.35	0.33	0.39	0.47	0.41	0.51	IMD 5	Controls
Pulmonary	0.20	0.18	0.20	0.24	0.23	0.27	0.28	0.26	0.30	IMD 5	Controls
Renal	0.11	0.10	0.12	0.14	0.13	0.18	0.22	0.18	0.26	IMD 5	Controls
Reproductive	0.30	0.25	0.32	0.39	0.33	0.41	0.52	0.45	0.53	IMD 5	Controls

Table S6. Cumulative burden for conditions grouped by organ systems and cancer diagnostic groups among survivors at 35, 40 and 45 years attained age. Cumulative burden per individual is shown.

	35 years			40 years			45 years			
**Health conditions by organ system**	**Cumulative Burden**	**Lower CI**	**Upper CI**	**Cumulative Burden**	**Lower CI**	**Upper CI**	**Cumulative Burden**	**Lower CI**	**Upper CI**	**Cancer diagnostic groups**
Cardiovascular	6.42	2.80	9.40	7.40	3.57	10.04	8.64	4.63	10.89	Leukaemias, myeloproliferative diseases, and myelodysplastic diseases
Endocrine	3.93	2.65	4.50	4.31	3.03	4.95	4.51	3.10	5.25	Leukaemias, myeloproliferative diseases, and myelodysplastic diseases
Gastrointestinal	1.46	1.25	1.87	1.67	1.52	2.22	1.85	1.65	2.39	Leukaemias, myeloproliferative diseases, and myelodysplastic diseases
Haematological	0.61	0.49	0.64	0.63	0.51	0.65	0.69	0.57	0.69	Leukaemias, myeloproliferative diseases, and myelodysplastic diseases
Immunology and infection	5.37	3.73	6.00	6.07	4.56	6.80	6.71	5.13	7.77	Leukaemias, myeloproliferative diseases, and myelodysplastic diseases
Mental health	2.51	1.37	2.79	3.16	1.65	3.71	3.54	2.22	4.43	Leukaemias, myeloproliferative diseases, and myelodysplastic diseases
Musculoskeletal, Ocular & Otorhinolaryngological	0.67	0.46	0.72	0.75	0.55	0.79	0.98	0.68	1.06	Leukaemias, myeloproliferative diseases, and myelodysplastic diseases
Neurological	0.56	0.33	0.67	0.68	0.44	0.91	0.73	0.49	0.99	Leukaemias, myeloproliferative diseases, and myelodysplastic diseases
Pulmonary	0.77	0.62	0.79	0.83	0.68	0.84	0.85	0.68	0.86	Leukaemias, myeloproliferative diseases, and myelodysplastic diseases
Renal	1.41	0.60	1.48	1.53	0.74	1.60	1.58	0.78	1.65	Leukaemias, myeloproliferative diseases, and myelodysplastic diseases
Reproductive	0.30	0.25	0.36	0.50	0.40	0.84	0.72	0.65	1.33	Leukaemias, myeloproliferative diseases, and myelodysplastic diseases
Cardiovascular	0.83	0.35	1.52	1.22	0.65	2.04	2.49	1.39	3.31	Lymphomas and reticuloendothelial neoplasms
Endocrine	1.06	0.88	1.20	1.40	1.16	1.79	1.76	1.20	2.32	Lymphomas and reticuloendothelial neoplasms
Gastrointestinal	0.59	0.35	0.75	0.91	0.60	1.14	1.22	0.94	1.40	Lymphomas and reticuloendothelial neoplasms
Haematological	0.29	0.16	0.36	0.34	0.20	0.43	0.38	0.25	0.47	Lymphomas and reticuloendothelial neoplasms
Immunology and infection	2.01	1.70	2.44	2.58	2.14	2.90	2.97	2.56	3.41	Lymphomas and reticuloendothelial neoplasms
Mental health	2.06	1.56	2.32	2.77	2.15	3.23	3.14	2.53	3.76	Lymphomas and reticuloendothelial neoplasms
Musculoskeletal, Ocular & Otorhinolaryngological	0.23	0.19	0.27	0.29	0.27	0.36	0.36	0.29	0.42	Lymphomas and reticuloendothelial neoplasms
Neurological	0.56	0.33	0.67	0.68	0.44	0.91	0.73	0.49	0.99	Lymphomas and reticuloendothelial neoplasms
Pulmonary	0.36	0.30	0.48	0.41	0.33	0.51	0.49	0.44	0.55	Lymphomas and reticuloendothelial neoplasms
Renal	0.31	0.21	0.39	0.48	0.32	0.58	0.63	0.32	0.73	Lymphomas and reticuloendothelial neoplasms
Reproductive	0.36	0.24	0.43	0.40	0.29	0.47	0.42	0.31	0.50	Lymphomas and reticuloendothelial neoplasms
Cardiovascular	1.61	1.12	1.94	1.77	1.27	2.14	2.28	1.93	2.55	CNS and miscellaneous intracranial and intraspinal neoplasms
Endocrine	2.33	1.86	2.70	2.53	2.03	3.06	3.11	2.54	3.68	CNS and miscellaneous intracranial and intraspinal neoplasms
Gastrointestinal	0.61	0.47	0.73	0.83	0.76	1.05	0.88	0.77	1.05	CNS and miscellaneous intracranial and intraspinal neoplasms
Haematological	0.13	0.10	0.18	0.13	0.10	0.19	0.13	0.10	0.19	CNS and miscellaneous intracranial and intraspinal neoplasms
Immunology and infection	1.76	1.58	1.98	2.12	1.85	2.37	2.58	2.19	2.82	CNS and miscellaneous intracranial and intraspinal neoplasms
Mental health	1.68	1.31	2.04	2.68	1.75	3.58	2.83	1.81	3.86	CNS and miscellaneous intracranial and intraspinal neoplasms
Musculoskeletal, Ocular & Otorhinolaryngological	0.28	0.24	0.29	0.32	0.27	0.31	0.48	0.33	0.55	CNS and miscellaneous intracranial and intraspinal neoplasms
Neurological	0.56	0.33	0.67	0.68	0.44	0.91	0.73	0.49	0.99	CNS and miscellaneous intracranial and intraspinal neoplasms
Pulmonary	0.40	0.31	0.42	0.40	0.32	0.43	0.45	0.36	0.49	CNS and miscellaneous intracranial and intraspinal neoplasms
Renal	0.30	0.24	0.38	0.40	0.36	0.48	0.67	0.53	1.03	CNS and miscellaneous intracranial and intraspinal neoplasms
Reproductive	0.28	0.23	0.31	0.29	0.23	0.32	0.36	0.27	0.38	CNS and miscellaneous intracranial and intraspinal neoplasms
Cardiovascular	0.32	0.18	0.40	0.97	0.65	1.31	1.07	0.71	1.42	Malignant bone tumours
Endocrine	0.38	0.21	0.49	0.45	0.30	0.54	0.59	0.44	0.89	Malignant bone tumours
Gastrointestinal	0.61	0.52	0.85	0.76	0.56	1.08	0.86	0.66	1.14	Malignant bone tumours
Haematological	0.14	0.11	0.20	0.17	0.11	0.25	0.17	0.11	0.25	Malignant bone tumours
Immunology and infection	2.33	1.75	2.40	3.26	2.13	3.46	3.32	2.13	3.46	Malignant bone tumours
Mental health	1.95	1.34	2.39	2.97	2.11	3.87	2.97	2.11	3.87	Malignant bone tumours
Musculoskeletal, Ocular & Otorhinolaryngological	0.29	0.16	0.33	0.37	0.23	0.47	0.42	0.23	0.52	Malignant bone tumours
Neurological	0.22	0.17	0.24	0.33	0.27	0.38	0.33	0.27	0.38	Malignant bone tumours
Pulmonary	0.37	0.28	0.53	0.48	0.37	0.68	0.48	0.37	0.68	Malignant bone tumours
Renal	0.32	0.13	0.45	0.54	0.13	0.78	0.62	0.22	0.91	Malignant bone tumours
Reproductive	0.15	0.08	0.19	0.15	0.08	0.19	0.15	0.08	0.19	Malignant bone tumours
Cardiovascular	0.41	0.22	0.60	0.53	0.34	0.73	2.17	0.86	4.77	Soft tissue and other extraosseous sarcomas
Endocrine	3.34	1.50	7.00	3.75	1.92	8.06	6.85	3.46	18.71	Soft tissue and other extraosseous sarcomas
Gastrointestinal	0.32	0.26	0.47	0.43	0.38	0.59	0.69	0.59	1.03	Soft tissue and other extraosseous sarcomas
Haematological	0.09	0.06	0.11	0.09	0.06	0.11	0.20	0.10	0.40	Soft tissue and other extraosseous sarcomas
Immunology and infection	1.79	1.05	2.84	2.01	1.21	3.12	4.69	3.23	8.52	Soft tissue and other extraosseous sarcomas
Mental health	2.67	2.70	4.00	3.21	3.06	4.93	4.40	4.40	7.38	Soft tissue and other extraosseous sarcomas
Musculoskeletal, Ocular & Otorhinolaryngological	0.21	0.20	0.28	0.25	0.22	0.34	0.34	0.35	0.48	Soft tissue and other extraosseous sarcomas
Neurological	0.36	0.35	0.56	0.43	0.40	0.60	0.50	0.53	0.66	Soft tissue and other extraosseous sarcomas
Pulmonary	0.53	0.50	0.67	0.56	0.54	0.69	0.61	0.61	0.77	Soft tissue and other extraosseous sarcomas
Renal	0.22	0.15	0.37	0.24	0.17	0.37	0.35	0.31	0.68	Soft tissue and other extraosseous sarcomas
Reproductive	0.15	0.14	0.25	0.21	0.20	0.34	0.26	0.26	0.36	Soft tissue and other extraosseous sarcomas
Cardiovascular	0.39	0.27	0.56	0.84	0.52	1.07	1.15	0.74	1.39	Germ cell tumours, trophoblastic tumours, and neoplasms of gonads
Endocrine	1.47	0.48	2.14	1.60	0.57	2.21	1.78	0.73	2.35	Germ cell tumours, trophoblastic tumours, and neoplasms of gonads
Gastrointestinal	0.56	0.41	0.86	0.64	0.47	0.97	0.91	0.74	1.13	Germ cell tumours, trophoblastic tumours, and neoplasms of gonads
Haematological	0.06	0.02	0.13	0.09	0.05	0.15	0.10	0.05	0.18	Germ cell tumours, trophoblastic tumours, and neoplasms of gonads
Immunology and infection	1.26	1.00	1.81	1.52	1.21	2.07	1.79	1.43	2.30	Germ cell tumours, trophoblastic tumours, and neoplasms of gonads
Mental health	1.61	1.40	2.21	1.97	1.79	2.49	2.60	2.14	3.36	Germ cell tumours, trophoblastic tumours, and neoplasms of gonads
Musculoskeletal, Ocular & Otorhinolaryngological	0.13	0.11	0.18	0.19	0.16	0.26	0.25	0.22	0.29	Germ cell tumours, trophoblastic tumours, and neoplasms of gonads
Neurological	0.36	0.35	0.56	0.43	0.40	0.60	0.50	0.53	0.66	Germ cell tumours, trophoblastic tumours, and neoplasms of gonads
Pulmonary	0.30	0.25	0.42	0.34	0.27	0.44	0.39	0.34	0.52	Germ cell tumours, trophoblastic tumours, and neoplasms of gonads
Renal	0.37	0.27	0.48	0.41	0.32	0.50	0.45	0.36	0.55	Germ cell tumours, trophoblastic tumours, and neoplasms of gonads
Reproductive	0.15	0.14	0.21	0.21	0.18	0.26	0.23	0.21	0.31	Germ cell tumours, trophoblastic tumours, and neoplasms of gonads
Cardiovascular	0.56	0.40	0.80	0.75	0.63	0.99	0.88	0.76	1.10	Other cancers
Endocrine	1.53	1.03	1.87	1.88	1.52	2.18	2.25	1.81	2.56	Other cancers
Gastrointestinal	0.47	0.43	0.62	0.72	0.66	0.85	0.96	0.90	1.18	Other cancers
Haematological	0.13	0.10	0.16	0.16	0.14	0.20	0.17	0.14	0.20	Other cancers
Immunology and infection	1.52	1.58	1.92	1.83	1.88	2.33	2.03	2.01	2.53	Other cancers
Mental health	2.15	1.61	2.43	2.91	2.20	3.25	3.46	2.65	3.94	Other cancers
Musculoskeletal, Ocular & Otorhinolaryngological	0.17	0.14	0.17	0.24	0.20	0.25	0.31	0.27	0.35	Other cancers
Neurological	0.48	0.40	0.60	0.56	0.48	0.67	0.63	0.54	0.72	Other cancers
Pulmonary	0.50	0.41	0.62	0.55	0.45	0.67	0.59	0.53	0.70	Other cancers
Renal	0.46	0.24	0.69	0.51	0.28	0.76	0.54	0.29	0.78	Other cancers
Reproductive	0.29	0.23	0.35	0.46	0.39	0.54	0.56	0.50	0.63	Other cancers

Table S7. Cumulative burden for conditions grouped by organ systems and treatment types among survivors at 35, 40 and 45 years attained age. Cumulative burden per individual is shown.

	35 years			40 years			45 years			
Health conditions by organ system	Cumulative Burden	Lower CI	Upper CI	Cumulative Burden	Lower CI	Upper CI	Cumulative Burden	Lower CI	Upper CI	Treatment type
Cardiovascular	3.20	1.89	4.31	3.54	2.01	4.76	7.52	4.12	12.40	Chemotherapy and radiotherapy
Endocrine	3.18	2.04	4.23	3.68	2.57	4.71	5.18	3.43	6.36	Chemotherapy and radiotherapy
Gastrointestinal	0.71	0.46	0.92	0.91	0.61	1.17	1.19	0.66	1.59	Chemotherapy and radiotherapy
Haematological	0.66	0.52	0.80	0.68	0.52	0.82	0.68	0.52	0.82	Chemotherapy and radiotherapy
Immunology and infection	10.88	5.72	15.76	11.55	6.64	16.66	12.06	6.90	16.86	Chemotherapy and radiotherapy
Mental health	2.61	1.49	3.61	2.88	1.58	4.20	2.91	1.62	4.20	Chemotherapy and radiotherapy
Musculoskeletal, Ocular & Otorhinolaryngological	1.52	0.79	2.74	1.63	0.94	2.87	1.77	1.10	2.99	Chemotherapy and radiotherapy
Neurological	1.00	0.41	1.53	1.16	0.55	1.63	1.27	0.75	1.68	Chemotherapy and radiotherapy
Pulmonary	0.88	0.66	1.06	0.95	0.70	1.14	0.98	0.73	1.14	Chemotherapy and radiotherapy
Renal	1.68	1.03	2.43	1.94	1.64	2.79	2.35	1.96	3.21	Chemotherapy and radiotherapy
Reproductive	0.19	0.12	0.22	0.36	0.15	0.56	0.58	0.15	1.02	Chemotherapy and radiotherapy
Cardiovascular	0.57	0.57	0.75	1.97	2.19	2.81	1.97	2.19	2.81	Chemotherapy and surgery
Endocrine	0.83	0.51	1.13	1.10	0.66	1.37	1.12	0.67	1.37	Chemotherapy and surgery
Gastrointestinal	0.58	0.46	0.69	0.87	0.65	1.11	1.09	0.84	1.35	Chemotherapy and surgery
Haematological	0.32	0.20	0.41	0.46	0.22	0.64	0.51	0.36	0.70	Chemotherapy and surgery
Immunology and infection	3.27	2.68	4.10	4.45	3.76	6.33	4.76	4.04	6.41	Chemotherapy and surgery
Mental health	2.50	1.68	2.81	3.76	2.55	4.12	4.34	3.06	5.03	Chemotherapy and surgery
Musculoskeletal, Ocular & Otorhinolaryngological	0.22	0.16	0.30	0.28	0.21	0.36	0.33	0.25	0.52	Chemotherapy and surgery
Neurological	0.31	0.19	0.36	0.48	0.40	0.53	0.53	0.40	0.59	Chemotherapy and surgery
Pulmonary	0.34	0.30	0.42	0.40	0.37	0.47	0.45	0.42	0.50	Chemotherapy and surgery
Renal	0.44	0.35	0.59	0.61	0.53	0.97	0.66	0.63	1.02	Chemotherapy and surgery
Reproductive	0.29	0.24	0.38	0.41	0.31	0.47	0.58	0.37	0.61	Chemotherapy and surgery
Cardiovascular	5.80	1.79	11.27	6.13	2.37	11.60	6.32	2.64	11.73	Chemotherapy only
Endocrine	3.37	1.48	5.61	3.41	1.57	5.65	3.51	1.75	5.76	Chemotherapy only
Gastrointestinal	0.87	0.69	1.07	1.00	0.80	1.27	1.25	0.99	1.53	Chemotherapy only
Haematological	0.27	0.20	0.36	0.30	0.25	0.40	0.30	0.25	0.40	Chemotherapy only
Immunology and infection	2.72	2.43	3.53	3.23	2.93	3.81	3.69	3.53	4.13	Chemotherapy only
Mental health	1.30	1.19	1.43	1.99	1.49	2.61	2.48	1.99	3.30	Chemotherapy only
Musculoskeletal, Ocular & Otorhinolaryngological	0.28	0.25	0.33	0.31	0.27	0.37	0.35	0.31	0.40	Chemotherapy only
Neurological	0.46	0.27	0.69	0.50	0.31	0.72	0.56	0.31	0.78	Chemotherapy only
Pulmonary	0.59	0.39	0.91	0.63	0.42	0.94	0.70	0.56	0.95	Chemotherapy only
Renal	1.19	0.32	2.21	1.36	0.44	2.33	1.37	0.47	2.35	Chemotherapy only
Reproductive	0.26	0.23	0.33	0.30	0.28	0.40	0.33	0.32	0.42	Chemotherapy only
Cardiovascular	1.40	0.40	2.57	1.49	0.45	2.57	1.74	0.58	2.62	Radiotherapy and surgery
Endocrine	2.57	1.73	3.51	2.94	2.23	3.94	3.43	2.79	4.21	Radiotherapy and surgery
Gastrointestinal	0.48	0.34	0.59	0.68	0.51	0.76	0.77	0.64	0.83	Radiotherapy and surgery
Haematological	0.26	0.18	0.32	0.31	0.26	0.36	0.31	0.26	0.36	Radiotherapy and surgery
Immunology and infection	2.54	1.61	3.37	2.92	1.97	3.73	3.55	2.56	4.87	Radiotherapy and surgery
Mental health	1.71	1.17	1.90	2.18	1.67	2.58	2.42	1.88	3.07	Radiotherapy and surgery
Musculoskeletal, Ocular & Otorhinolaryngological	0.17	0.11	0.21	0.22	0.15	0.26	0.24	0.19	0.27	Radiotherapy and surgery
Neurological	0.50	0.35	0.55	0.56	0.39	0.61	0.72	0.58	0.86	Radiotherapy and surgery
Pulmonary	0.47	0.26	0.54	0.57	0.40	0.60	0.60	0.43	0.67	Radiotherapy and surgery
Renal	0.44	0.32	0.52	0.54	0.47	0.65	0.58	0.51	0.78	Radiotherapy and surgery
Reproductive	0.20	0.15	0.27	0.39	0.24	0.51	0.45	0.27	0.60	Radiotherapy and surgery
Cardiovascular	1.86	1.17	2.81	2.31	1.17	3.21	2.43	1.17	3.35	Radiotherapy only
Endocrine	3.19	2.72	5.40	3.71	3.18	5.75	3.88	3.39	5.83	Radiotherapy only
Gastrointestinal	0.81	0.54	1.16	0.95	0.63	1.35	0.95	0.63	1.35	Radiotherapy only
Haematological	0.40	0.24	0.42	0.44	0.29	0.47	0.44	0.29	0.47	Radiotherapy only
Immunology and infection	3.13	1.81	3.94	3.35	2.07	4.08	3.35	2.07	4.08	Radiotherapy only
Mental health	2.03	1.06	3.02	2.29	1.08	3.56	3.54	2.59	7.23	Radiotherapy only
Musculoskeletal, Ocular & Otorhinolaryngological	0.39	0.32	0.47	0.54	0.45	0.68	0.59	0.45	0.73	Radiotherapy only
Neurological	0.82	0.57	0.98	0.95	0.76	1.08	1.00	0.76	1.12	Radiotherapy only
Pulmonary	0.49	0.33	0.73	0.55	0.42	0.78	0.55	0.42	0.78	Radiotherapy only
Renal	0.36	0.27	0.47	0.38	0.27	0.50	0.43	0.34	0.57	Radiotherapy only
Reproductive	0.28	0.23	0.39	0.32	0.28	0.51	0.32	0.28	0.51	Radiotherapy only
Cardiovascular	0.55	0.44	0.67	0.72	0.54	0.92	0.93	0.77	1.15	Surgery only
Endocrine	1.51	1.05	1.63	1.78	1.34	1.98	2.11	1.67	2.34	Surgery only
Gastrointestinal	0.58	0.51	0.72	0.78	0.72	0.90	1.01	0.93	1.17	Surgery only
Haematological	0.17	0.08	0.23	0.19	0.11	0.25	0.20	0.13	0.27	Surgery only
Immunology and infection	1.66	1.40	1.75	1.92	1.70	2.02	2.12	1.88	2.30	Surgery only
Mental health	1.98	1.74	2.45	2.71	2.27	3.20	3.27	2.76	3.79	Surgery only
Musculoskeletal, Ocular & Otorhinolaryngological	0.20	0.18	0.22	0.27	0.24	0.29	0.35	0.32	0.37	Surgery only
Neurological	0.54	0.50	0.70	0.61	0.57	0.77	0.67	0.61	0.80	Surgery only
Pulmonary	0.41	0.36	0.45	0.46	0.43	0.51	0.51	0.47	0.56	Surgery only
Renal	0.33	0.29	0.41	0.38	0.34	0.47	0.43	0.40	0.53	Surgery only
Reproductive	0.27	0.23	0.32	0.37	0.32	0.40	0.43	0.36	0.49	Surgery only

Table S8. Cumulative burden for conditions grouped by organ systems and chemotherapy types among survivors at 35, 40 and 45 years attained age. Cumulative burden per individual is shown.

	35 years			40 years			
Health conditions by organ system	Cumulative Burden	Lower CI	Upper CI	Cumulative Burden	Lower CI	Upper CI	Chemotherapy type
Cardiovascular	0.42	0.24	0.49	1.87	1.27	2.70	Alkylating agents
Endocrine	0.62	0.52	0.79	1.30	0.64	2.07	Alkylating agents
Gastrointestinal	0.54	0.34	0.86	0.95	0.79	1.24	Alkylating agents
Haematological	0.61	0.25	0.85	0.81	0.41	1.16	Alkylating agents
Immunology and infection	2.44	1.99	2.63	3.09	2.47	3.82	Alkylating agents
Mental health	3.18	2.22	4.67	5.06	3.09	8.00	Alkylating agents
Musculoskeletal, Ocular & Otorhinolaryngological	0.51	0.27	0.75	0.55	0.31	0.80	Alkylating agents
Neurological	0.53	0.36	0.60	0.61	0.39	0.69	Alkylating agents
Pulmonary	0.65	0.39	0.72	0.77	0.51	0.87	Alkylating agents
Renal	0.47	0.29	0.44	0.64	0.46	0.64	Alkylating agents
Reproductive	0.33	0.17	0.48	0.40	0.17	0.69	Alkylating agents
Cardiovascular	0.99	0.47	1.16	4.07	2.48	5.71	Anthracyclines
Endocrine	0.85	0.64	1.20	1.63	1.23	2.40	Anthracyclines
Gastrointestinal	0.55	0.44	0.88	1.00	0.72	1.32	Anthracyclines
Haematological	0.55	0.33	0.65	0.79	0.69	0.89	Anthracyclines
Immunology and infection	3.12	2.91	3.99	4.43	4.02	5.24	Anthracyclines
Mental health	2.82	2.30	3.03	4.69	3.34	6.58	Anthracyclines
Musculoskeletal, Ocular & Otorhinolaryngological	0.34	0.13	0.44	0.43	0.24	0.55	Anthracyclines
Neurological	0.28	0.21	0.34	0.38	0.26	0.58	Anthracyclines
Pulmonary	0.42	0.29	0.55	0.58	0.44	0.73	Anthracyclines
Renal	0.43	0.34	0.50	0.65	0.44	0.77	Anthracyclines
Reproductive	0.28	0.19	0.34	0.35	0.24	0.45	Anthracyclines
Cardiovascular	2.27	1.72	3.62	6.23	3.23	8.32	Antimetabolites
Endocrine	0.85	0.61	1.73	1.77	0.72	2.11	Antimetabolites
Gastrointestinal	1.89	0.50	2.07	2.15	0.73	2.40	Antimetabolites
Haematological	1.28	0.90	1.67	1.64	0.98	1.89	Antimetabolites
Immunology and infection	6.08	5.51	8.74	10.27	7.06	14.92	Antimetabolites
Mental health	3.04	1.41	4.39	5.45	2.74	7.11	Antimetabolites
Musculoskeletal, Ocular & Otorhinolaryngological	0.19	0.09	0.19	0.25	0.10	0.42	Antimetabolites
Neurological	0.23	0.16	0.30	0.41	0.24	0.53	Antimetabolites
Pulmonary	0.49	0.47	0.83	0.67	0.56	1.07	Antimetabolites
Renal	0.84	0.50	1.38	2.51	0.70	4.19	Antimetabolites
Reproductive	0.35	0.18	0.52	0.46	0.27	0.68	Antimetabolites
Cardiovascular	0.65	0.58	0.83	1.44	1.07	1.67	Chemotherapy unspecified
Endocrine	1.25	0.75	1.61	1.48	1.17	1.90	Chemotherapy unspecified
Gastrointestinal	0.72	0.42	0.77	0.90	0.57	1.07	Chemotherapy unspecified
Haematological	0.24	0.18	0.23	0.30	0.25	0.29	Chemotherapy unspecified
Immunology and infection	2.72	2.33	2.92	3.60	3.11	4.23	Chemotherapy unspecified
Mental health	1.51	1.07	1.75	2.25	1.69	2.67	Chemotherapy unspecified
Musculoskeletal, Ocular & Otorhinolaryngological	0.29	0.22	0.35	0.36	0.28	0.43	Chemotherapy unspecified
Neurological	0.43	0.33	0.58	0.57	0.47	0.75	Chemotherapy unspecified
Pulmonary	0.38	0.30	0.40	0.45	0.36	0.47	Chemotherapy unspecified
Renal	0.40	0.32	0.50	0.62	0.47	0.83	Chemotherapy unspecified
Reproductive	0.30	0.23	0.32	0.35	0.27	0.39	Chemotherapy unspecified
Cardiovascular	0.32	0.20	0.45	0.37	0.23	0.56	Hormonal agents (including corticosteroid hormones and sex hormones)
Endocrine	0.74	0.50	0.98	0.91	0.63	1.30	Hormonal agents (including corticosteroid hormones and sex hormones)
Gastrointestinal	1.32	0.82	2.09	1.84	1.32	2.61	Hormonal agents (including corticosteroid hormones and sex hormones)
Haematological	0.72	0.62	0.95	0.75	0.62	0.95	Hormonal agents (including corticosteroid hormones and sex hormones)
Immunology and infection	3.26	2.51	3.92	4.03	3.13	4.82	Hormonal agents (including corticosteroid hormones and sex hormones)
Mental health	1.70	1.06	2.57	2.76	1.41	4.28	Hormonal agents (including corticosteroid hormones and sex hormones)
Musculoskeletal, Ocular & Otorhinolaryngological	0.19	0.08	0.25	0.19	0.08	0.25	Hormonal agents (including corticosteroid hormones and sex hormones)
Neurological	0.45	0.27	0.55	0.48	0.30	0.57	Hormonal agents (including corticosteroid hormones and sex hormones)
Pulmonary	0.43	0.27	0.51	0.48	0.31	0.54	Hormonal agents (including corticosteroid hormones and sex hormones)
Renal	0.54	0.30	0.48	1.11	0.51	1.55	Hormonal agents (including corticosteroid hormones and sex hormones)
Reproductive	0.29	0.12	0.47	0.41	0.22	0.77	Hormonal agents (including corticosteroid hormones and sex hormones)
Cardiovascular	0.10	0.04	0.15	0.10	0.04	0.15	Non-anthracycline antitumour antibiotics
Endocrine	0.30	0.26	0.43	0.39	0.36	0.64	Non-anthracycline antitumour antibiotics
Gastrointestinal	0.53	0.39	0.88	0.84	0.39	0.93	Non-anthracycline antitumour antibiotics
Haematological	0.15	0.08	0.24	0.15	0.08	0.24	Non-anthracycline antitumour antibiotics
Immunology and infection	1.64	1.65	2.35	1.69	1.65	2.35	Non-anthracycline antitumour antibiotics
Mental health	1.96	1.89	3.92	3.70	2.73	6.27	Non-anthracycline antitumour antibiotics
Musculoskeletal, Ocular & Otorhinolaryngological	0.58	0.10	1.21	0.72	0.36	1.21	Non-anthracycline antitumour antibiotics
Neurological	0.29	0.21	0.45	0.29	0.21	0.45	Non-anthracycline antitumour antibiotics
Pulmonary	0.75	0.54	1.19	0.75	0.54	1.19	Non-anthracycline antitumour antibiotics
Renal	0.18	0.11	0.29	0.18	0.11	0.29	Non-anthracycline antitumour antibiotics
Reproductive	0.07	0.02	0.09	0.07	0.02	0.09	Non-anthracycline antitumour antibiotics
Cardiovascular	1.76	0.95	3.09	5.47	3.55	9.82	Plant alkaloids and natural products (excluding vinca alkaloids)
Endocrine	0.68	0.25	1.05	1.34	0.59	2.28	Plant alkaloids and natural products (excluding vinca alkaloids)
Gastrointestinal	0.69	0.33	0.86	0.94	0.56	1.09	Plant alkaloids and natural products (excluding vinca alkaloids)
Haematological	0.20	0.10	0.26	0.46	0.26	0.82	Plant alkaloids and natural products (excluding vinca alkaloids)
Immunology and infection	2.61	1.57	3.49	5.08	2.67	6.36	Plant alkaloids and natural products (excluding vinca alkaloids)
Mental health	3.60	1.77	4.57	5.96	2.85	7.64	Plant alkaloids and natural products (excluding vinca alkaloids)
Musculoskeletal, Ocular & Otorhinolaryngological	0.33	0.06	0.52	0.38	0.15	0.57	Plant alkaloids and natural products (excluding vinca alkaloids)
Neurological	0.77	0.49	1.12	0.88	0.51	1.14	Plant alkaloids and natural products (excluding vinca alkaloids)
Pulmonary	0.43	0.40	0.59	0.57	0.43	0.72	Plant alkaloids and natural products (excluding vinca alkaloids)
Renal	0.42	0.11	0.52	1.13	0.25	1.26	Plant alkaloids and natural products (excluding vinca alkaloids)
Reproductive	0.37	0.10	0.47	0.45	0.10	0.62	Plant alkaloids and natural products (excluding vinca alkaloids)
Cardiovascular	1.21	0.85	2.05	4.24	1.00	6.03	Platinum agents
Endocrine	0.48	0.03	0.75	1.06	0.05	1.73	Platinum agents
Gastrointestinal	0.89	0.48	1.31	1.08	0.87	1.69	Platinum agents
Haematological	0.38	0.20	0.43	0.69	0.24	0.83	Platinum agents
Immunology and infection	3.12	2.62	3.82	6.27	3.59	10.06	Platinum agents
Mental health	4.83	2.49	8.94	7.04	4.91	13.02	Platinum agents
Musculoskeletal, Ocular & Otorhinolaryngological	0.13	0.05	0.14	0.18	0.06	0.25	Platinum agents
Neurological	1.10	0.46	1.91	1.19	0.61	1.99	Platinum agents
Pulmonary	0.26	0.11	0.43	0.39	0.19	0.48	Platinum agents
Renal	0.61	0.26	1.03	1.86	0.57	3.08	Platinum agents
Reproductive	0.41	0.24	0.63	0.49	0.24	0.95	Platinum agents
Cardiovascular	0.25	0.09	0.28	0.25	0.09	0.28	Vinca alkaloids
Endocrine	0.96	0.49	1.32	1.25	0.49	1.72	Vinca alkaloids
Gastrointestinal	1.73	0.45	1.73	2.05	0.87	2.25	Vinca alkaloids
Haematological	0.62	0.30	0.77	0.62	0.30	0.77	Vinca alkaloids
Immunology and infection	2.60	2.04	3.10	2.77	2.04	3.44	Vinca alkaloids
Mental health	1.79	1.34	2.37	3.22	1.99	4.58	Vinca alkaloids
Musculoskeletal, Ocular & Otorhinolaryngological	0.26	0.04	0.30	0.33	0.06	0.40	Vinca alkaloids
Neurological	0.50	0.33	0.61	0.61	0.44	0.94	Vinca alkaloids
Pulmonary	0.56	0.45	0.72	0.90	0.72	1.20	Vinca alkaloids
Renal	0.38	0.25	0.39	0.38	0.25	0.39	Vinca alkaloids
Reproductive	0.12	0.05	0.18	0.12	0.05	0.18	Vinca alkaloids

Table S9. Cumulative burden for hospital in-patient admissions and critical care admissions for all survivors and controls at 35, 40 and 45 years attained age. Cumulative burden per individual is shown.

	35 years			40 years			45 years				
**Hospital event type**	**Cumulative Burden**	**Lower CI**	**Upper CI**	**Cumulative Burden**	**Lower CI**	**Upper CI**	**Cumulative Burden**	**Lower CI**	**Upper CI**	**Groups**	**IMD strata**
Hospital in-patient admissions	3.384	3.184	3.589	4.064	3.972	4.214	4.638	4.605	4.819	All survivors	Overall
Hospital in-patient admissions	2.258	2.050	2.539	2.932	2.598	3.430	3.580	3.033	4.282	All survivors	IMD 1
Hospital in-patient admissions	3.961	3.768	4.358	4.989	4.668	5.775	5.923	5.687	6.663	All survivors	IMD 5
Hospital critical care admissions	0.100	0.084	0.118	0.118	0.104	0.132	0.126	0.112	0.138	All survivors	Overall
Hospital in-patient admissions	1.060	1.044	1.072	1.447	1.403	1.468	1.844	1.825	1.878	Controls	Overall
Hospital in-patient admissions	0.860	0.823	0.922	1.170	1.165	1.233	1.439	1.388	1.553	Controls	IMD 1
Hospital in-patient admissions	1.332	1.239	1.400	1.862	1.723	1.917	2.412	2.213	2.535	Controls	IMD 5
Hospital critical care admissions	0.015	0.013	0.017	0.023	0.020	0.027	0.037	0.031	0.042	Controls	Overall

Table S10. Cumulative burden for hospital in-patient admissions by cancer diagnostic groups among survivors at 35, 40 and 45 years attained age. Cumulative burden per individual is shown.

	35 years			40 years			45 years		
**Cancer diagnostic groups**	**Cumulative Burden**	**Lower CI**	**Upper CI**	**Cumulative Burden**	**Lower CI**	**Upper CI**	**Cumulative Burden**	**Lower CI**	**Upper CI**
Leukaemias, myeloproliferative diseases, and myelodysplastic diseases	6.20	5.60	6.33	7.24	6.61	7.36	7.91	7.00	8.05
Lymphomas and reticuloendothelial neoplasms	2.91	2.57	3.23	3.93	3.42	4.36	4.62	3.96	4.74
CNS and miscellaneous intracranial and intraspinal neoplasms	3.74	3.49	3.87	4.54	4.09	4.67	6.13	4.52	6.80
Malignant bone tumours	3.38	2.27	3.87	4.02	2.92	4.61	4.09	3.01	4.61
Soft tissue and other extraosseous sarcomas	2.73	2.25	4.45	3.12	2.66	5.03	4.15	4.46	7.84
Germ cell tumours, trophoblastic tumours, and neoplasms of gonads	1.86	1.65	2.34	2.32	2.13	2.69	2.91	2.46	3.44
Other cancers	2.72	2.33	3.23	3.39	2.97	4.11	3.77	3.28	4.55

Table S11. Cumulative burden for hospital in-patient admissions by treatment type among survivors at 35, 40 and 45 years attained age. Cumulative burden per individual is shown.

	35 years			40 years			45 years		
**Treatment type**	**Cumulative Burden**	**Lower CI**	**Upper CI**	**Cumulative Burden**	**Lower CI**	**Upper CI**	**Cumulative Burden**	**Lower CI**	**Upper CI**
Chemotherapy and radiotherapy	7.99	5.80	9.18	9.22	7.26	10.83	10.43	8.27	11.95
Chemotherapy and surgery	3.91	3.25	5.22	5.41	4.55	7.51	5.73	4.98	7.63
Chemotherapy only	3.84	3.42	5.18	4.48	4.02	5.55	4.89	4.60	5.74
Radiotherapy and surgery	5.16	4.61	6.79	6.22	5.81	8.08	7.38	6.68	9.13
Radiotherapy only	3.84	3.45	4.69	4.11	3.62	5.02	4.29	3.87	5.64
Surgery only	2.32	2.17	2.45	2.87	2.66	2.91	3.37	3.16	3.42

Table S12. Cumulative burden for hospital in-patient admissions by chemotherapy type among survivors at 35, 40 and 45 years attained age. Cumulative burden per individual is shown.

	35 years				40 years		
**Chemotherapy type**	**Cumulative Burden**	**Lower CI**	**Upper CI**		**Cumulative Burden**	**Lower CI**	**Upper CI**
Alkylating agents	4.15	3.38	4.60		6.08	5.11	7.16
Anthracyclines	4.00	3.67	4.53		7.48	6.60	8.12
Antimetabolites	8.39	5.97	11.23		13.76	8.51	18.41
Chemotherapy unspecified	4.08	3.69	4.36		4.97	4.53	5.32
Hormonal agents (including corticosteroid hormones and sex hormones)	5.87	4.28	6.03		7.41	5.84	7.71
Non-anthracycline antitumour antibiotics	2.25	1.96	2.66		2.79	2.09	3.28
Plant alkaloids and natural products (excluding vinca alkaloids)	3.54	2.70	4.16		7.31	5.34	8.14
Platinum agents	4.55	3.26	5.05		8.46	5.20	10.53
Vinca alkaloids	3.23	2.62	2.99		4.29	3.48	4.64

Table S13. Multivariable logistic regression analysis for the association between treatment exposures and diagnoses of health conditions among cancer survivors.

Health conditions by organ system	Adjusted odds ratio	Lower CI	Upper CI	P value	Treatment types
Cardiovascular	0.85	0.66	1.09	0.189	All surgery
Cardiovascular	1.38	1.07	1.78	0.012	All chemotherapy
Cardiovascular	1.78	1.33	2.36	< 0.001	All radiotherapy
Cardiovascular	0.94	0.66	1.31	0.720	Chemotherapy only
Cardiovascular	0.73	0.56	0.94	0.016	Surgery only
Cardiovascular	1.63	0.93	2.68	0.069	Radiotherapy only
Cardiovascular	2.62	1.67	3.97	< 0.001	Chemotherapy and radiotherapy
Cardiovascular	1.29	0.85	1.90	0.207	Chemotherapy and surgery
Cardiovascular	1.23	0.72	1.98	0.420	Radiotherapy and surgery
Endocrine	1.18	0.96	1.44	0.114	All surgery
Endocrine	0.96	0.77	1.18	0.692	All chemotherapy
Endocrine	1.99	1.57	2.50	< 0.001	All radiotherapy
Endocrine	0.82	0.61	1.07	0.155	Chemotherapy only
Endocrine	0.99	0.81	1.21	0.929	Surgery only
Endocrine	1.86	1.19	2.81	0.004	Radiotherapy only
Endocrine	1.59	1.04	2.38	0.028	Chemotherapy and radiotherapy
Endocrine	0.78	0.52	1.13	0.208	Chemotherapy and surgery
Endocrine	2.10	1.45	2.97	< 0.001	Radiotherapy and surgery
Gastrointestinal	0.87	0.70	1.08	0.208	All surgery
Gastrointestinal	1.19	0.95	1.49	0.130	All chemotherapy
Gastrointestinal	1.25	0.94	1.63	0.112	All radiotherapy
Gastrointestinal	1.18	0.88	1.56	0.255	Chemotherapy only
Gastrointestinal	0.88	0.71	1.10	0.265	Surgery only
Gastrointestinal	1.39	0.83	2.21	0.190	Radiotherapy only
Gastrointestinal	1.49	0.93	2.29	0.084	Chemotherapy and radiotherapy
Gastrointestinal	0.97	0.65	1.40	0.874	Chemotherapy and surgery
Gastrointestinal	1.01	0.61	1.57	0.981	Radiotherapy and surgery
Haematological	0.73	0.55	0.98	0.034	All surgery
Haematological	2.06	1.54	2.75	< 0.001	All chemotherapy
Haematological	2.13	1.53	2.92	< 0.001	All radiotherapy
Haematological	1.25	0.86	1.79	0.228	Chemotherapy only
Haematological	0.51	0.37	0.70	< 0.001	Surgery only
Haematological	1.31	0.63	2.43	0.423	Radiotherapy only
Haematological	3.43	2.12	5.34	< 0.001	Chemotherapy and radiotherapy
Haematological	1.56	0.98	2.40	0.050	Chemotherapy and surgery
Haematological	1.45	0.80	2.44	0.188	Radiotherapy and surgery
Immunology and infection	1.00	0.84	1.18	0.967	All surgery
Immunology and infection	1.48	1.24	1.75	< 0.001	All chemotherapy
Immunology and infection	1.75	1.42	2.14	< 0.001	All radiotherapy
Immunology and infection	1.07	0.86	1.33	0.532	Chemotherapy only
Immunology and infection	0.84	0.71	0.99	0.041	Surgery only
Immunology and infection	0.94	0.60	1.42	0.783	Radiotherapy only
Immunology and infection	3.00	2.14	4.19	< 0.001	Chemotherapy and radiotherapy
Immunology and infection	1.17	0.88	1.55	0.274	Chemotherapy and surgery
Immunology and infection	1.50	1.07	2.08	0.017	Radiotherapy and surgery
Mental health	1.13	0.95	1.36	0.175	All surgery
Mental health	0.92	0.76	1.12	0.417	All chemotherapy
Mental health	1.26	1.01	1.58	0.042	All radiotherapy
Mental health	0.82	0.64	1.05	0.126	Chemotherapy only
Mental health	1.09	0.91	1.30	0.366	Surgery only
Mental health	1.18	0.75	1.79	0.453	Radiotherapy only
Mental health	1.37	0.92	2.01	0.109	Chemotherapy and radiotherapy
Mental health	0.97	0.70	1.32	0.852	Chemotherapy and surgery
Mental health	1.35	0.93	1.91	0.103	Radiotherapy and surgery
Musculoskeletal, Ocular & Otorhinolaryngological	0.79	0.64	0.98	0.030	All surgery
Musculoskeletal, Ocular & Otorhinolaryngological	1.14	0.91	1.42	0.265	All chemotherapy
Musculoskeletal, Ocular & Otorhinolaryngological	1.51	1.16	1.94	0.002	All radiotherapy
Musculoskeletal, Ocular & Otorhinolaryngological	1.00	0.74	1.33	0.991	Chemotherapy only
Musculoskeletal, Ocular & Otorhinolaryngological	0.86	0.69	1.07	0.191	Surgery only
Musculoskeletal, Ocular & Otorhinolaryngological	1.62	1.00	2.53	0.041	Radiotherapy only
Musculoskeletal, Ocular & Otorhinolaryngological	2.30	1.52	3.38	< 0.001	Chemotherapy and radiotherapy
Musculoskeletal, Ocular & Otorhinolaryngological	0.80	0.52	1.17	0.273	Chemotherapy and surgery
Musculoskeletal, Ocular & Otorhinolaryngological	0.93	0.56	1.46	0.757	Radiotherapy and surgery
Neoplasm	0.72	0.61	0.85	< 0.001	All surgery
Neoplasm	2.14	1.81	2.54	< 0.001	All chemotherapy
Neoplasm	3.24	2.67	3.94	< 0.001	All radiotherapy
Neoplasm	1.40	1.12	1.72	0.002	Chemotherapy only
Neoplasm	0.46	0.39	0.55	< 0.001	Surgery only
Neoplasm	1.79	1.22	2.59	0.002	Radiotherapy only
Neoplasm	4.09	2.94	5.70	< 0.001	Chemotherapy and radiotherapy
Neoplasm	1.07	0.80	1.42	0.635	Chemotherapy and surgery
Neoplasm	2.17	1.57	2.96	< 0.001	Radiotherapy and surgery
Neoplasm metastasis	1.02	0.71	1.48	0.913	All surgery
Neoplasm metastasis	2.50	1.73	3.59	< 0.001	All chemotherapy
Neoplasm metastasis	4.59	3.18	6.60	< 0.001	All radiotherapy
Neoplasm metastasis	1.45	0.90	2.26	0.112	Chemotherapy only
Neoplasm metastasis	0.43	0.28	0.65	< 0.001	Surgery only
Neoplasm metastasis	1.31	0.51	2.80	0.528	Radiotherapy only
Neoplasm metastasis	3.85	2.17	6.46	< 0.001	Chemotherapy and radiotherapy
Neoplasm metastasis	0.95	0.46	1.75	0.889	Chemotherapy and surgery
Neoplasm metastasis	4.03	2.42	6.44	< 0.001	Radiotherapy and surgery
Neurological	0.99	0.81	1.22	0.939	All surgery
Neurological	0.92	0.73	1.15	0.467	All chemotherapy
Neurological	2.27	1.79	2.86	< 0.001	All radiotherapy
Neurological	0.66	0.48	0.90	0.010	Chemotherapy only
Neurological	0.84	0.68	1.04	0.104	Surgery only
Neurological	2.53	1.67	3.76	< 0.001	Radiotherapy only
Neurological	1.69	1.09	2.53	0.014	Chemotherapy and radiotherapy
Neurological	0.73	0.48	1.08	0.128	Chemotherapy and surgery
Neurological	1.74	1.18	2.52	0.004	Radiotherapy and surgery
Pulmonary	1.10	0.90	1.35	0.353	All surgery
Pulmonary	1.09	0.88	1.35	0.410	All chemotherapy
Pulmonary	1.61	1.26	2.04	< 0.001	All radiotherapy
Pulmonary	0.76	0.57	1.02	0.072	Chemotherapy only
Pulmonary	0.99	0.81	1.21	0.939	Surgery only
Pulmonary	1.21	0.73	1.92	0.429	Radiotherapy only
Pulmonary	2.34	1.58	3.39	< 0.001	Chemotherapy and radiotherapy
Pulmonary	1.09	0.76	1.52	0.635	Chemotherapy and surgery
Pulmonary	1.41	0.94	2.06	0.086	Radiotherapy and surgery
Renal	0.65	0.51	0.82	< 0.001	All surgery
Renal	1.91	1.50	2.43	< 0.001	All chemotherapy
Renal	2.23	1.70	2.91	< 0.001	All radiotherapy
Renal	1.33	0.99	1.79	0.056	Chemotherapy only
Renal	0.51	0.39	0.66	< 0.001	Surgery only
Renal	1.50	0.86	2.47	0.130	Radiotherapy only
Renal	3.83	2.57	5.60	< 0.001	Chemotherapy and radiotherapy
Renal	1.24	0.82	1.81	0.283	Chemotherapy and surgery
Renal	1.62	1.01	2.48	0.036	Radiotherapy and surgery
Reproductive	1.18	0.92	1.53	0.195	All surgery
Reproductive	0.96	0.73	1.25	0.744	All chemotherapy
Reproductive	0.98	0.69	1.35	0.899	All radiotherapy
Reproductive	0.82	0.56	1.17	0.288	Chemotherapy only
Reproductive	1.14	0.89	1.46	0.298	Surgery only
Reproductive	1.17	0.60	2.08	0.620	Radiotherapy only
Reproductive	0.87	0.44	1.58	0.676	Chemotherapy and radiotherapy
Reproductive	1.16	0.73	1.76	0.513	Chemotherapy and surgery
Reproductive	0.78	0.41	1.34	0.395	Radiotherapy and surgery

Table S14. Multivariable logistic regression analysis for the association between chemotherapy cumulative dose and diagnoses of health conditions among cancer survivors. Patients were stratified by median values of cumulative dose of different chemotherapeutic agents. Strata with low number of outcome events (n<5) were not analysed.

Health conditions by organ system	Adjusted odds ratio	Lower CI	Upper CI	P value	Chemotherapy dose
Cardiovascular	0.214	0.012	0.985	0.128	Alkylating agents < median
Cardiovascular	0.213	0.012	0.978	0.127	Alkylating agents ≥ median
Cardiovascular	0.244	0.014	1.128	0.165	Anthracyclines < median
Cardiovascular	0.255	0.014	1.177	0.178	Anthracyclines ≥ median
Cardiovascular	0.397	0.022	1.878	0.366	Antimetabolites < median
Cardiovascular	1.798	0.527	4.669	0.279	Antimetabolites ≥ median
Cardiovascular	0.313	0.018	1.458	0.253	Plant alkaloids and natural products excluding vinca alkaloids < median
Cardiovascular	0.693	0.112	2.302	0.616	Plant alkaloids and natural products excluding vinca alkaloids ≥ median
Cardiovascular	0.784	0.126	2.630	0.741	Platinum agents ≥ median
Endocrine	0.212	0.034	0.690	0.032	Alkylating agents < median
Endocrine	0.233	0.038	0.757	0.044	Alkylating agents ≥ median
Endocrine	0.645	0.220	1.510	0.361	Anthracyclines < median
Endocrine	0.687	0.162	1.984	0.543	Antimetabolites ≥ median
Endocrine	0.188	0.011	0.884	0.101	Hormonal agents including corticosteroid hormones and sex hormones < median
Endocrine	0.163	0.009	0.761	0.075	Non-anthracycline antitumour antibiotics < median
Endocrine	0.324	0.052	1.078	0.124	Plant alkaloids and natural products excluding vinca alkaloids < median
Endocrine	0.465	0.075	1.552	0.297	Plant alkaloids and natural products excluding vinca alkaloids ≥ median
Endocrine	0.272	0.015	1.288	0.202	Platinum agents < median
Endocrine	0.229	0.013	1.083	0.148	Platinum agents ≥ median
Endocrine	0.461	0.111	1.290	0.201	Vinca alkaloid < median
Endocrine	0.149	0.008	0.691	0.061	Vinca alkaloid ≥ median
Gastrointestinal	0.605	0.182	1.502	0.339	Alkylating agents < median
Gastrointestinal	0.508	0.122	1.407	0.26	Anthracyclines < median
Gastrointestinal	0.523	0.084	1.760	0.379	Antimetabolites < median
Gastrointestinal	0.557	0.090	1.866	0.426	Antimetabolites ≥ median
Gastrointestinal	0.215	0.012	1.005	0.131	Hormonal agents including corticosteroid hormones and sex hormones < median
Gastrointestinal	0.228	0.013	1.061	0.146	Hormonal agents including corticosteroid hormones and sex hormones ≥ median
Gastrointestinal	0.214	0.012	0.996	0.129	Non-anthracycline antitumour antibiotics ≥ median
Gastrointestinal	0.415	0.067	1.373	0.229	Plant alkaloids and natural products excluding vinca alkaloids < median
Gastrointestinal	0.451	0.073	1.496	0.276	Plant alkaloids and natural products excluding vinca alkaloids ≥ median
Gastrointestinal	0.788	0.187	2.244	0.696	Platinum agents ≥ median
Gastrointestinal	0.381	0.062	1.250	0.185	Vinca alkaloid < median
Haematological	0.951	0.228	2.647	0.933	Alkylating agents < median
Haematological	0.654	0.106	2.141	0.559	Alkylating agents ≥ median
Haematological	1.075	0.257	3.015	0.905	Anthracyclines < median
Haematological	1.198	0.287	3.360	0.766	Anthracyclines ≥ median
Haematological	1.278	0.204	4.361	0.741	Antimetabolites < median
Haematological	6.793	2.768	15.148	< 0.001	Antimetabolites ≥ median
Haematological	1.648	0.391	4.709	0.415	Hormonal agents including corticosteroid hormones and sex hormones ≥ median
Haematological	0.458	0.026	2.147	0.443	Non-anthracycline antitumour antibiotics < median
Haematological	0.945	0.152	3.167	0.938	Plant alkaloids and natural products excluding vinca alkaloids < median
Haematological	2.043	0.484	5.865	0.245	Plant alkaloids and natural products excluding vinca alkaloids ≥ median
Haematological	1.615	0.257	5.538	0.519	Platinum agents < median
Haematological	2.187	0.515	6.352	0.206	Platinum agents ≥ median
Haematological	1.757	0.519	4.473	0.292	Vinca alkaloid < median
Immunology and infection	0.442	0.168	0.965	0.062	Alkylating agents < median
Immunology and infection	0.214	0.052	0.584	0.01	Alkylating agents ≥ median
Immunology and infection	0.410	0.141	0.953	0.062	Anthracyclines < median
Immunology and infection	0.348	0.104	0.869	0.045	Anthracyclines ≥ median
Immunology and infection	0.736	0.246	1.797	0.538	Antimetabolites < median
Immunology and infection	1.902	0.844	4.023	0.103	Antimetabolites ≥ median
Immunology and infection	0.650	0.220	1.552	0.377	Hormonal agents including corticosteroid hormones and sex hormones < median
Immunology and infection	0.482	0.143	1.231	0.173	Hormonal agents including corticosteroid hormones and sex hormones ≥ median
Immunology and infection	0.239	0.039	0.787	0.05	Non-anthracycline antitumour antibiotics ≥ median
Immunology and infection	0.308	0.074	0.866	0.052	Plant alkaloids and natural products excluding vinca alkaloids < median
Immunology and infection	0.681	0.231	1.617	0.429	Plant alkaloids and natural products excluding vinca alkaloids ≥ median
Immunology and infection	0.856	0.287	2.078	0.754	Platinum agents < median
Immunology and infection	0.770	0.259	1.858	0.595	Platinum agents ≥ median
Immunology and infection	0.601	0.226	1.336	0.253	Vinca alkaloid < median
Mental health	0.441	0.152	1.017	0.084	Alkylating agents < median
Mental health	0.566	0.215	1.235	0.194	Alkylating agents ≥ median
Mental health	1.015	0.455	2.037	0.97	Anthracyclines < median
Mental health	0.319	0.077	0.882	0.057	Anthracyclines ≥ median
Mental health	0.489	0.116	1.405	0.244	Antimetabolites < median
Mental health	1.158	0.424	2.693	0.752	Antimetabolites ≥ median
Mental health	0.282	0.045	0.937	0.084	Hormonal agents including corticosteroid hormones and sex hormones < median
Mental health	0.275	0.044	0.913	0.078	Hormonal agents including corticosteroid hormones and sex hormones ≥ median
Mental health	0.250	0.040	0.823	0.057	Non-anthracycline antitumour antibiotics < median
Mental health	0.478	0.115	1.341	0.223	Non-anthracycline antitumour antibiotics ≥ median
Mental health	0.679	0.230	1.615	0.426	Plant alkaloids and natural products excluding vinca alkaloids < median
Mental health	0.491	0.117	1.383	0.241	Plant alkaloids and natural products excluding vinca alkaloids ≥ median
Mental health	0.389	0.062	1.308	0.2	Platinum agents < median
Mental health	1.215	0.447	2.811	0.672	Platinum agents ≥ median
Mental health	0.471	0.140	1.183	0.155	Vinca alkaloid < median
Mental health	0.617	0.211	1.448	0.316	Vinca alkaloid ≥ median
Musculoskeletal, Ocular & Otorhinolaryngological	0.282	0.046	0.916	0.081	Alkylating agents < median
Musculoskeletal, Ocular & Otorhinolaryngological	0.158	0.009	0.729	0.069	Anthracyclines < median
Musculoskeletal, Ocular & Otorhinolaryngological	0.256	0.014	1.206	0.181	Antimetabolites < median
Musculoskeletal, Ocular & Otorhinolaryngological	0.182	0.010	0.842	0.093	Vinca alkaloid ≥ median
Neoplasm	1.805	0.968	3.220	0.052	Alkylating agents < median
Neoplasm	0.510	0.195	1.108	0.123	Alkylating agents ≥ median
Neoplasm	0.977	0.439	1.951	0.95	Anthracyclines < median
Neoplasm	0.386	0.116	0.960	0.07	Anthracyclines ≥ median
Neoplasm	1.233	0.485	2.770	0.632	Antimetabolites < median
Neoplasm	2.323	1.063	4.836	0.027	Antimetabolites ≥ median
Neoplasm	0.667	0.226	1.587	0.405	Hormonal agents including corticosteroid hormones and sex hormones < median
Neoplasm	0.664	0.225	1.576	0.399	Hormonal agents including corticosteroid hormones and sex hormones ≥ median
Neoplasm	0.102	0.006	0.471	0.024	Non-anthracycline antitumour antibiotics < median
Neoplasm	0.104	0.006	0.482	0.026	Non-anthracycline antitumour antibiotics ≥ median
Neoplasm	0.609	0.207	1.440	0.306	Plant alkaloids and natural products excluding vinca alkaloids < median
Neoplasm	0.773	0.289	1.740	0.568	Plant alkaloids and natural products excluding vinca alkaloids ≥ median
Neoplasm	0.583	0.171	1.504	0.317	Platinum agents < median
Neoplasm	0.563	0.166	1.446	0.286	Platinum agents ≥ median
Neoplasm	1.421	0.679	2.762	0.322	Vinca alkaloid < median
Neoplasm	0.309	0.075	0.857	0.051	Vinca alkaloid ≥ median
Neoplasm metastasis	1.001	0.162	3.308	0.998	Alkylating agents < median
Neoplasm metastasis	0.517	0.029	2.392	0.515	Alkylating agents ≥ median
Neoplasm metastasis	1.790	0.427	5.050	0.339	Anthracyclines < median
Neoplasm metastasis	3.131	0.735	9.148	0.066	Antimetabolites < median
Neoplasm metastasis	4.435	1.290	11.656	0.006	Antimetabolites ≥ median
Neoplasm metastasis	0.779	0.044	3.692	0.807	Hormonal agents including corticosteroid hormones and sex hormones < median
Neoplasm metastasis	2.372	0.563	6.780	0.159	Plant alkaloids and natural products excluding vinca alkaloids < median
Neoplasm metastasis	3.751	1.093	9.809	0.015	Plant alkaloids and natural products excluding vinca alkaloids ≥ median
Neoplasm metastasis	0.978	0.054	4.690	0.983	Platinum agents < median
Neoplasm metastasis	2.856	0.673	8.295	0.09	Platinum agents ≥ median
Neoplasm metastasis	0.625	0.035	2.918	0.644	Vinca alkaloid < median
Neurological	0.823	0.313	1.800	0.657	Alkylating agents < median
Neurological	0.128	0.007	0.584	0.042	Alkylating agents ≥ median
Neurological	0.285	0.046	0.931	0.084	Anthracyclines < median
Neurological	0.304	0.049	0.991	0.101	Anthracyclines ≥ median
Neurological	0.481	0.077	1.619	0.32	Antimetabolites < median
Neurological	0.764	0.182	2.181	0.661	Antimetabolites ≥ median
Neurological	0.640	0.153	1.807	0.463	Hormonal agents including corticosteroid hormones and sex hormones < median
Neurological	0.418	0.068	1.385	0.233	Hormonal agents including corticosteroid hormones and sex hormones ≥ median
Neurological	0.862	0.256	2.186	0.781	Non-anthracycline antitumour antibiotics ≥ median
Neurological	0.183	0.010	0.849	0.093	Plant alkaloids and natural products excluding vinca alkaloids < median
Neurological	1.462	0.545	3.310	0.401	Plant alkaloids and natural products excluding vinca alkaloids ≥ median
Neurological	1.474	0.494	3.578	0.432	Platinum agents < median
Neurological	1.041	0.306	2.686	0.94	Platinum agents ≥ median
Neurological	0.334	0.054	1.093	0.131	Vinca alkaloid < median
Neurological	0.164	0.009	0.756	0.074	Vinca alkaloid ≥ median
Pulmonary	0.732	0.278	1.603	0.478	Alkylating agents < median
Pulmonary	0.117	0.007	0.533	0.034	Alkylating agents ≥ median
Pulmonary	0.690	0.236	1.608	0.438	Anthracyclines < median
Pulmonary	0.205	0.011	0.966	0.12	Antimetabolites < median
Pulmonary	1.959	0.770	4.391	0.124	Antimetabolites ≥ median
Pulmonary	0.570	0.136	1.611	0.356	Hormonal agents including corticosteroid hormones and sex hormones < median
Pulmonary	0.159	0.009	0.738	0.07	Non-anthracycline antitumour antibiotics < median
Pulmonary	0.186	0.010	0.864	0.098	Non-anthracycline antitumour antibiotics ≥ median
Pulmonary	0.330	0.053	1.093	0.129	Plant alkaloids and natural products excluding vinca alkaloids < median
Pulmonary	0.845	0.250	2.155	0.753	Plant alkaloids and natural products excluding vinca alkaloids ≥ median
Pulmonary	0.231	0.013	1.089	0.151	Platinum agents < median
Pulmonary	0.430	0.069	1.439	0.25	Platinum agents ≥ median
Pulmonary	0.473	0.114	1.315	0.214	Vinca alkaloid < median
Pulmonary	0.309	0.050	1.014	0.107	Vinca alkaloid ≥ median
Renal	1.430	0.541	3.148	0.418	Alkylating agents < median
Renal	0.905	0.271	2.247	0.849	Alkylating agents ≥ median
Renal	1.308	0.447	3.063	0.577	Anthracyclines < median
Renal	0.788	0.189	2.191	0.692	Anthracyclines ≥ median
Renal	2.374	0.790	5.830	0.083	Antimetabolites < median
Renal	3.484	1.362	7.865	0.005	Antimetabolites ≥ median
Renal	0.756	0.122	2.523	0.703	Hormonal agents including corticosteroid hormones and sex hormones < median
Renal	1.464	0.431	3.763	0.479	Hormonal agents including corticosteroid hormones and sex hormones ≥ median
Renal	0.305	0.017	1.419	0.243	Non-anthracycline antitumour antibiotics < median
Renal	0.973	0.232	2.750	0.964	Plant alkaloids and natural products excluding vinca alkaloids < median
Renal	1.184	0.282	3.351	0.782	Plant alkaloids and natural products excluding vinca alkaloids ≥ median
Renal	1.452	0.343	4.192	0.545	Platinum agents < median
Renal	1.347	0.320	3.858	0.627	Platinum agents ≥ median
Renal	1.172	0.348	2.958	0.765	Vinca alkaloid < median
Renal	0.570	0.092	1.875	0.44	Vinca alkaloid ≥ median
Reproductive	0.151	0.008	0.697	0.062	Alkylating agents < median
Reproductive	0.171	0.010	0.796	0.082	Anthracyclines < median
Reproductive	0.331	0.018	1.590	0.281	Antimetabolites < median
Reproductive	0.352	0.020	1.687	0.309	Antimetabolites ≥ median
Reproductive	0.317	0.018	1.507	0.261	Hormonal agents including corticosteroid hormones and sex hormones < median
Reproductive	0.257	0.014	1.211	0.183	Plant alkaloids and natural products excluding vinca alkaloids < median
Reproductive	0.443	0.025	2.114	0.426	Plant alkaloids and natural products excluding vinca alkaloids ≥ median
Reproductive	0.418	0.023	2.012	0.395	Platinum agents ≥ median

Table S15. Multivariable logistic regression analysis for the association between radiotherapy dose or number of teletherapy fields and diagnoses of health conditions among cancer survivors. Teletherapy fields are denoted as the actual number of fields used to deliver a fraction.

Health conditions by organ system	Adjusted odds ratio	Lower CI	Upper CI	P value	Radiotherapy dose or number of fields
Cardiovascular	2.552	0.946	5.820	0.04	Radiation dose < 20Gy
Cardiovascular	1.466	0.603	3.039	0.347	Radiation dose 21 - 35Gy
Cardiovascular	1.534	0.453	3.911	0.425	Radiation dose 36 - 49Gy
Cardiovascular	1.973	0.856	3.984	0.079	Radiation dose ≥ 50Gy
Cardiovascular	1.113	0.491	2.188	0.776	≤ 3 fields
Cardiovascular	2.576	1.453	4.318	0.001	> 3 fields
Endocrine	1.540	0.609	3.409	0.318	Radiation dose < 20Gy
Endocrine	1.168	0.533	2.277	0.671	Radiation dose 21 - 35Gy
Endocrine	0.960	0.325	2.289	0.933	Radiation dose 36 - 49Gy
Endocrine	1.387	0.653	2.666	0.357	Radiation dose ≥ 50Gy
Endocrine	1.637	0.949	2.692	0.062	≤ 3 fields
Endocrine	0.917	0.470	1.638	0.784	> 3 fields
Gastrointestinal	1.656	0.615	3.767	0.267	Radiation dose < 20Gy
Gastrointestinal	0.505	0.153	1.235	0.189	Radiation dose 21 - 35Gy
Gastrointestinal	0.683	0.164	1.916	0.529	Radiation dose 36 - 49Gy
Gastrointestinal	1.071	0.440	2.228	0.866	Radiation dose ≥ 50Gy
Gastrointestinal	0.798	0.370	1.515	0.525	≤ 3 fields
Gastrointestinal	0.984	0.492	1.783	0.96	> 3 fields
Haematological	5.039	2.086	10.938	< 0.001	Radiation dose < 20Gy
Haematological	4.607	2.290	8.597	< 0.001	Radiation dose 21 - 35Gy
Haematological	1.008	0.162	3.378	0.991	Radiation dose 36 - 49Gy
Haematological	3.431	1.544	6.830	0.001	Radiation dose ≥ 50Gy
Haematological	4.609	2.635	7.714	< 0.001	≤ 3 fields
Haematological	2.584	1.310	4.682	0.003	> 3 fields
Immunology and infection	1.884	0.896	3.770	0.081	Radiation dose < 20Gy
Immunology and infection	1.774	1.020	2.976	0.035	Radiation dose 21 - 35Gy
Immunology and infection	1.387	0.634	2.798	0.383	Radiation dose 36 - 49Gy
Immunology and infection	1.455	0.789	2.555	0.208	Radiation dose ≥ 50Gy
Immunology and infection	1.722	1.094	2.649	0.016	≤ 3 fields
Immunology and infection	1.520	0.956	2.353	0.067	> 3 fields
Mental health	1.036	0.412	2.275	0.934	Radiation dose < 20Gy
Mental health	1.919	1.090	3.243	0.018	Radiation dose 21 - 35Gy
Mental health	1.817	0.853	3.619	0.101	Radiation dose 36 - 49Gy
Mental health	0.606	0.249	1.258	0.218	Radiation dose ≥ 50Gy
Mental health	1.716	1.071	2.672	0.02	≤ 3 fields
Mental health	0.921	0.523	1.530	0.763	> 3 fields
Musculoskeletal, Ocular & Otorhinolaryngological	1.664	0.619	3.780	0.262	Radiation dose < 20Gy
Musculoskeletal, Ocular & Otorhinolaryngological	0.520	0.157	1.272	0.208	Radiation dose 21 - 35Gy
Musculoskeletal, Ocular & Otorhinolaryngological	1.252	0.425	2.968	0.643	Radiation dose 36 - 49Gy
Musculoskeletal, Ocular & Otorhinolaryngological	0.755	0.261	1.726	0.551	Radiation dose ≥ 50Gy
Musculoskeletal, Ocular & Otorhinolaryngological	0.920	0.444	1.703	0.805	≤ 3 fields
Musculoskeletal, Ocular & Otorhinolaryngological	0.905	0.438	1.676	0.769	> 3 fields
Neoplasm	3.172	1.566	6.235	0.001	Radiation dose < 20Gy
Neoplasm	3.984	2.441	6.451	< 0.001	Radiation dose 21 - 35Gy
Neoplasm	3.709	1.897	7.098	< 0.001	Radiation dose 36 - 49Gy
Neoplasm	3.774	2.223	6.337	< 0.001	Radiation dose ≥ 50Gy
Neoplasm	4.505	3.002	6.748	< 0.001	≤ 3 fields
Neoplasm	3.061	2.013	4.602	< 0.001	> 3 fields
Neoplasm metastasis	2.516	0.402	8.542	0.212	Radiation dose < 20Gy
Neoplasm metastasis	9.767	4.887	18.358	< 0.001	Radiation dose 21 - 35Gy
Neoplasm metastasis	12.421	5.320	26.602	< 0.001	Radiation dose 36 - 49Gy
Neoplasm metastasis	5.509	2.213	11.856	< 0.001	Radiation dose ≥ 50Gy
Neoplasm metastasis	9.603	5.379	16.505	< 0.001	≤ 3 fields
Neoplasm metastasis	5.533	2.759	10.268	< 0.001	> 3 fields
Neurological	1.605	0.597	3.647	0.297	Radiation dose < 20Gy
Neurological	2.562	1.400	4.444	0.001	Radiation dose 21 - 35Gy
Neurological	2.848	1.300	5.757	0.005	Radiation dose 36 - 49Gy
Neurological	3.239	1.776	5.656	< 0.001	Radiation dose ≥ 50Gy
Neurological	2.160	1.284	3.484	0.002	≤ 3 fields
Neurological	3.103	1.945	4.823	< 0.001	> 3 fields
Pulmonary	2.300	1.007	4.793	0.034	Radiation dose < 20Gy
Pulmonary	2.960	1.697	4.980	< 0.001	Radiation dose 21 - 35Gy
Pulmonary	1.460	0.585	3.170	0.373	Radiation dose 36 - 49Gy
Pulmonary	1.210	0.551	2.368	0.603	Radiation dose ≥ 50Gy
Pulmonary	2.046	1.239	3.256	0.004	≤ 3 fields
Pulmonary	1.899	1.141	3.038	0.01	> 3 fields
Renal	8.647	4.261	17.195	< 0.001	Radiation dose < 20Gy
Renal	2.386	1.129	4.558	0.014	Radiation dose 21 - 35Gy
Renal	1.958	0.661	4.680	0.169	Radiation dose 36 - 49Gy
Renal	1.703	0.697	3.565	0.194	Radiation dose ≥ 50Gy
Renal	3.075	1.774	5.084	< 0.001	≤ 3 fields
Renal	2.789	1.591	4.647	< 0.001	> 3 fields
Reproductive	0.852	0.202	2.446	0.795	Radiation dose < 20Gy
Reproductive	0.938	0.322	2.170	0.893	Radiation dose 21 - 35Gy
Reproductive	0.730	0.174	2.077	0.607	Radiation dose 36 - 49Gy
Reproductive	0.342	0.056	1.117	0.14	Radiation dose ≥ 50Gy
Reproductive	0.878	0.386	1.740	0.732	≤ 3 fields
Reproductive	0.517	0.180	1.168	0.157	> 3 fields

Table S16. Cumulative burden of 183 condition-specific outcomes in cancer survivors (by cancer diagnostic groups) and controls at 45 years attained age. Cumulative burden per 100 individuals is shown.

Health conditions	Controls			Leukaemias, myeloproliferative diseases, and myelodysplastic diseases			Lymphomas and reticuloendothelial neoplasms			CNS and miscellaneous intracranial and intraspinal neoplasms			Malignant bone tumours			Soft tissue and other extraosseous sarcomas			Germ cell tumours, trophoblastic tumours, and neoplasms of gonads			Other cancers		
	Cumulative burden	Lower CI	Upper CI	Cumulative burden	Lower CI	Upper CI	Cumulative burden	Lower CI	Upper CI	Cumulative burden	Lower CI	Upper CI	Cumulative burden	Lower CI	Upper CI	Cumulative burden	Lower CI	Upper CI	Cumulative burden	Lower CI	Upper CI	Cumulative burden	Lower CI	Upper CI
Mental health	268.05	246.21	286.79	353.51	222.04	443.39	313.66	252.73	376.34	282.57	180.89	386.39	296.75	210.84	387.16	439.73	440.48	738.10	259.73	213.83	336.05	346.31	264.75	393.86
Bacterial infections	30.00	28.36	31.50	104.57	91.38	111.97	74.86	64.58	84.87	70.14	62.31	77.97	126.36	82.28	165.64	134.95	43.30	336.60	57.40	44.51	65.29	53.83	43.64	58.58
Hypertension	26.76	20.48	29.50	146.40	90.05	150.77	34.75	17.46	53.14	69.14	46.48	86.27	58.44	0.00	147.04	140.58	0.00	254.46	30.85	16.64	42.54	31.17	19.26	42.47
Migraine	23.05	21.40	24.69	11.98	7.34	14.97	18.39	11.80	21.02	31.94	20.34	44.76	4.22	0.00	6.76	8.81	0.00	12.84	20.06	13.45	29.32	36.20	30.99	43.17
Hypo or hyperthyroidism	20.84	17.83	25.29	79.29	54.03	84.84	115.27	87.72	160.53	163.76	146.22	206.78	7.91	0.00	15.14	24.05	0.00	35.74	124.45	84.72	221.27	145.66	109.49	178.84
Menorrhagia and polymenorrhoea	17.40	16.23	18.57	38.73	6.42	82.91	15.02	11.15	18.48	12.73	6.91	15.67	3.23	0.00	4.74	3.83	0.00	5.06	7.43	5.36	12.14	25.96	22.49	32.77
Asthma	14.88	14.44	15.34	21.85	19.16	26.52	23.38	19.84	25.25	23.54	20.17	24.80	26.54	18.68	29.94	21.84	11.35	27.15	24.53	21.70	26.56	33.45	28.00	38.39
Oesophagitis and oesophageal ulcer	12.86	11.64	13.86	24.70	16.89	48.79	24.02	21.39	30.85	10.09	6.02	11.69	25.72	7.56	28.47	9.78	2.07	18.87	21.33	11.72	35.57	17.31	14.29	24.35
Diabetic ophthalmic complications	11.85	10.24	13.63	147.63	60.79	195.78	19.79	11.59	27.95	56.72	41.34	69.58	10.44	0.91	23.87	174.71	0.00	268.13	16.72	10.70	24.93	15.27	5.79	29.88
Obesity	11.54	11.17	12.69	10.32	6.19	11.94	13.11	10.02	15.36	21.99	16.29	26.22	14.58	6.36	18.22	16.69	10.26	20.01	18.78	12.53	26.69	19.21	16.24	23.81
Infections of other or unspecified organs	9.86	9.11	10.86	86.82	66.52	116.94	31.13	22.57	39.05	47.88	26.83	49.60	35.08	10.09	52.19	51.65	25.91	52.83	25.56	19.08	35.25	21.01	18.12	23.08
Enthesopathies and synovial disorders	9.61	9.29	10.24	20.71	11.45	28.41	9.38	7.30	11.00	14.22	10.05	22.53	12.39	3.71	12.86	5.47	2.16	10.04	8.31	6.59	10.75	12.69	11.31	14.61
Urolithiasis	9.27	5.13	12.82	7.91	4.13	12.97	25.50	11.66	30.69	3.53	1.48	4.47	22.57	7.09	39.34	4.93	0.00	9.27	8.70	6.33	13.83	9.25	2.43	13.92
Diabetes	9.20	8.68	9.36	13.11	9.12	14.96	11.72	10.00	12.36	12.60	7.18	19.06	18.53	9.70	20.08	13.29	5.65	16.15	10.20	9.24	10.92	15.12	10.79	16.82
Diabetic neurological complications	8.85	8.04	10.24	131.21	55.10	174.45	19.79	11.47	29.10	50.34	36.96	65.55	9.45	0.00	14.59	169.18	0.00	263.48	10.26	5.16	14.49	15.00	5.78	23.27
Infection of skin and subcutaneous tissues	8.68	8.20	9.27	41.22	31.00	53.48	16.43	12.83	19.26	11.84	7.94	16.11	23.21	12.03	25.66	10.88	3.99	14.10	7.63	3.70	10.92	14.70	12.46	18.17
Ear and upper respiratory tract infections	8.44	7.90	8.77	27.54	10.22	42.28	13.94	11.62	16.45	14.07	10.21	19.69	17.37	6.26	22.54	17.51	4.36	31.19	7.30	5.36	8.35	6.77	5.80	8.49
Cholelithiasis	7.92	7.32	9.11	8.21	4.89	10.89	6.21	3.74	8.96	12.65	8.04	17.63	7.70	0.00	11.56	2.36	0.00	3.51	15.36	10.60	18.23	9.89	8.46	13.76
HIV	7.05	6.83	7.49	12.80	7.88	16.68	7.01	5.24	8.70	4.55	2.72	7.05	13.42	2.25	33.54	26.66	1.03	57.14	3.68	2.23	4.81	6.34	5.12	7.10
Lower respiratory tract infections	7.05	6.98	7.80	123.34	100.33	154.38	31.62	27.11	47.24	22.99	13.54	24.89	23.43	13.87	28.71	36.88	18.19	32.69	11.09	5.70	16.79	11.96	9.20	15.20
Gastrooesophageal reflux disease	6.99	6.89	7.33	9.38	6.15	12.49	12.28	10.08	14.16	10.42	6.82	11.80	7.72	2.62	10.86	7.84	2.07	15.05	8.87	6.66	10.78	7.42	6.54	8.96
Coronary heart disease not otherwise specified	6.86	6.57	7.50	32.37	7.63	32.12	26.58	8.20	29.78	3.74	0.00	7.05	9.02	2.23	17.74	17.96	3.11	32.86	10.87	4.23	14.33	8.68	6.68	11.10
Dysmenorrhoea	6.63	6.27	7.80	8.48	0.94	12.49	11.92	7.89	21.19	8.27	4.98	10.13	0.00	0.00	0.00	3.62	0.00	5.41	2.70	1.03	2.82	11.87	8.77	14.54
Urinary incontinence	6.48	5.62	6.82	13.45	6.22	23.08	7.25	5.24	10.22	39.25	21.73	68.27	12.22	0.00	27.17	10.17	3.79	16.97	14.43	9.59	20.22	16.23	12.28	21.12
Infections of the digestive system	6.36	6.21	6.88	62.70	45.32	86.42	17.58	12.02	23.82	12.79	8.03	15.08	17.22	4.22	28.32	32.18	2.54	60.19	10.09	6.80	12.61	13.67	8.63	17.51
Urinary tract infections	6.23	5.70	7.67	24.70	11.36	28.19	14.37	10.82	18.99	24.60	16.90	30.01	29.32	6.40	39.72	53.84	5.52	79.30	18.31	13.54	25.55	18.24	14.40	23.75
Peritonitis	6.12	5.88	6.99	22.31	10.20	33.53	12.22	10.06	12.13	18.23	11.25	21.99	13.96	5.71	15.23	3.90	0.00	9.23	12.71	9.08	19.72	12.21	9.40	13.13
Cholecystitis	6.06	5.47	7.22	7.26	4.16	10.10	7.32	4.36	9.07	13.80	5.97	16.61	5.24	0.00	7.68	2.36	0.00	3.51	9.47	5.33	14.35	6.79	5.17	8.40
Endometriosis	5.64	4.87	6.57	3.95	0.34	5.34	2.01	0.80	2.12	6.58	0.93	7.37	0.00	0.00	0.00	4.08	0.00	7.31	0.43	0.20	0.66	9.11	7.02	12.74
Ulcerative colitis	5.59	3.52	6.69	21.32	0.55	38.47	10.08	2.65	12.40	8.13	1.14	16.29	0.00	0.00	0.00	0.00	0.00	0.00	0.00	0.00	0.00	16.66	3.38	19.10
Cholangitis	5.51	4.80	5.80	8.03	5.28	11.64	13.59	5.26	18.51	9.88	4.09	12.07	5.24	0.00	7.68	2.36	0.00	3.51	9.89	5.89	13.16	6.42	5.06	8.37
Cancer (breast)	5.47	2.08	7.01	19.27	0.00	40.10	19.04	2.00	20.16	0.00	0.00	0.00	0.00	0.00	0.00	0.00	0.00	0.00	13.96	2.72	17.28	80.33	54.19	106.84
Chronic sinusitis	5.20	4.93	5.76	7.81	5.25	9.32	6.88	5.17	8.62	10.11	4.58	16.95	10.73	4.28	14.50	7.61	2.21	10.17	3.25	2.10	4.11	6.46	6.04	7.23
Polycystic ovarian syndrome	5.14	3.80	5.86	12.58	6.94	20.48	1.62	0.78	1.65	4.98	1.31	6.16	0.00	0.00	0.00	5.16	0.00	8.80	1.42	0.83	1.62	4.45	3.56	7.34
Gastritis and duodenitis	4.79	4.49	5.07	12.65	6.42	19.65	6.46	5.34	8.35	6.78	4.03	7.99	5.74	0.00	9.65	8.06	1.91	14.49	5.44	3.80	6.86	6.19	4.92	7.03
Anorectal fistula	4.48	4.15	5.01	1.76	0.33	3.11	9.08	3.15	13.23	4.81	1.34	5.71	10.66	1.30	26.21	5.62	0.00	8.29	5.50	2.10	7.06	3.84	2.38	5.56
Postviral fatigue syndrome, neurasthenia and fibromyalgia	4.30	4.14	4.57	10.85	8.50	13.18	5.73	3.69	7.39	13.92	9.67	15.67	12.60	7.51	15.64	12.39	3.33	14.93	5.26	3.16	6.38	8.38	5.08	11.08
Hepatic failure	4.25	3.31	5.51	32.42	16.05	48.03	10.79	5.19	16.55	2.91	1.18	3.51	3.62	0.00	4.41	17.34	0.00	37.58	4.41	1.84	6.75	9.65	7.74	12.07
Viral diseases (excluding chronic hepatitis/HIV)	4.24	4.11	4.58	48.01	40.19	67.12	21.29	13.41	28.07	7.96	4.56	8.79	14.14	6.44	20.24	8.28	3.20	11.64	7.69	5.35	11.75	8.64	7.93	9.37
Chronic viral hepatitis	4.12	3.92	4.40	3.84	2.41	4.74	7.16	5.58	9.09	3.70	2.02	4.81	1.02	0.00	2.31	6.76	0.00	19.73	4.17	2.22	5.86	8.92	6.99	11.29
Pancreatitis	4.05	1.69	4.79	9.73	3.50	14.10	8.12	0.66	17.44	1.93	0.84	2.44	1.18	0.00	2.18	7.30	0.00	20.71	0.64	0.26	0.65	1.67	0.16	3.17
Other anaemias	3.73	3.47	4.07	14.40	7.07	17.28	7.79	6.27	8.96	6.04	3.31	7.04	15.80	7.45	17.73	13.11	5.20	19.37	2.23	0.98	2.45	6.81	4.36	10.12
Venous thromboembolic disease	3.14	2.20	4.20	11.19	6.04	15.39	2.17	0.94	2.91	19.07	9.88	20.33	7.68	0.00	15.48	1.81	0.00	4.08	7.89	2.35	9.02	4.66	2.59	5.80
Osteoarthritis (excluding spine)	2.97	2.52	3.22	4.88	2.25	5.51	4.32	2.17	6.24	5.91	1.96	7.29	1.24	0.00	2.65	0.00	0.00	0.00	2.54	1.19	2.71	2.83	1.47	3.88
Cancer metastasis (other organs)	2.86	1.50	5.02	26.01	0.33	29.35	9.66	3.65	9.92	56.75	25.46	71.02	260.54	71.06	277.12	110.36	13.02	136.28	41.34	24.65	53.27	111.18	82.29	148.13
COPD	2.82	2.76	3.08	4.80	2.70	6.86	3.65	1.65	4.63	3.02	0.00	6.26	3.23	0.00	3.84	5.54	1.08	9.75	5.23	3.64	8.04	3.41	2.35	5.03
Intracranial hypertension	2.62	1.29	3.98	30.15	1.17	56.08	0.26	0.00	0.28	13.63	10.04	15.08	0.00	0.00	0.00	5.63	0.00	11.38	1.75	0.88	2.59	1.89	0.81	2.73
Septicaemia	2.42	2.28	2.73	35.05	25.12	42.99	15.23	9.94	20.35	13.91	8.68	16.89	13.18	3.84	20.06	6.73	2.05	10.12	1.95	1.08	2.46	9.48	6.53	13.28
Diverticular disease of intestine	2.41	1.53	3.05	0.61	0.00	1.33	2.61	0.84	3.28	4.21	1.12	4.39	3.34	0.00	4.22	0.00	0.00	0.00	5.76	1.61	8.43	1.13	0.64	1.21
Coeliac disease	2.35	2.20	2.66	0.85	0.36	0.91	2.05	1.03	2.46	1.00	0.43	1.14	1.24	0.00	2.46	2.78	0.00	3.98	2.23	1.22	2.59	3.18	1.11	4.47
Erectile dysfunction	2.20	1.91	2.30	2.74	1.64	3.47	5.96	3.44	6.93	2.49	0.89	3.15	0.00	0.00	0.00	5.90	0.00	9.93	6.73	5.36	7.72	1.38	0.68	1.91
Epilepsy	2.14	1.89	2.31	5.95	2.90	5.83	3.50	1.92	4.15	45.44	40.62	52.26	1.99	0.00	3.08	7.06	3.77	10.57	7.59	4.02	10.71	6.85	4.33	10.74
Fatty liver	2.11	1.88	2.47	9.46	5.86	11.47	4.10	3.12	5.39	2.82	0.83	3.45	2.72	0.00	4.00	4.43	0.00	6.37	1.53	0.88	2.04	3.48	3.00	4.13
Nonrheumatic mitral valve disorders	2.11	0.41	5.32	1.95	0.37	2.62	6.39	3.81	8.12	0.63	0.00	1.38	0.00	0.00	0.00	5.34	0.00	10.85	1.92	0.29	2.30	0.00	0.00	0.00
Heart failure	1.98	1.24	2.62	33.10	19.18	42.92	26.40	13.82	45.48	6.43	2.87	7.02	6.47	0.00	10.51	4.40	0.00	6.71	3.48	1.68	3.95	0.57	0.00	1.08
Hypertrophy of nasal turbinates	1.92	1.83	2.12	3.16	0.32	5.08	1.59	0.68	1.64	1.48	0.51	2.31	4.61	0.00	7.69	1.53	0.00	3.68	3.97	2.25	5.93	1.93	1.16	2.29
Peripheral neuropathies	1.88	1.74	2.03	8.46	1.64	8.27	3.56	1.34	5.17	2.76	0.99	3.68	8.98	2.38	15.78	7.28	2.29	12.98	4.29	2.22	5.66	4.19	2.90	4.88
Cancer metastasis (retroperitoneum and peritoneum	1.88	0.61	3.58	23.52	0.00	37.99	6.53	0.80	11.70	0.00	0.00	53.72	231.76	162.19	319.06	103.67	13.02	136.28	39.84	28.35	51.61	66.45	40.03	105.75
Cancer metastasis (pleura)	1.88	0.61	3.58	23.52	0.00	37.99	6.53	0.80	11.70	0.00	0.00	53.72	231.76	162.19	319.06	103.67	13.02	136.28	39.84	28.35	51.61	66.45	40.03	105.75
Cancer metastasis (lung)	1.88	0.61	3.58	23.52	0.00	37.99	6.53	0.80	11.70	0.00	0.00	53.72	244.36	75.12	283.29	103.67	13.02	136.28	41.09	28.35	55.10	67.62	30.64	88.74
Cancer metastasis (liver and intrahepatic bile duct)	1.88	0.61	3.58	23.52	0.00	37.99	6.97	0.80	12.56	0.00	0.00	53.72	231.21	162.19	317.93	103.67	13.02	136.28	39.84	28.35	51.61	66.93	40.14	105.78
Cancer metastasis (bowel)	1.88	0.61	3.58	23.52	0.00	37.99	6.53	0.80	11.70	0.00	0.00	53.72	231.76	162.19	319.06	103.67	13.02	136.28	39.84	28.35	51.61	66.45	40.03	105.75
Meningitis	1.82	1.57	1.86	21.85	14.22	25.49	4.65	3.18	5.86	5.15	2.55	6.00	6.56	1.61	9.58	4.54	0.00	7.94	4.60	1.34	8.11	6.27	4.80	8.15
Other cardiomyopathy	1.73	1.22	2.52	186.64	43.99	454.58	48.18	23.04	76.12	6.17	1.85	6.99	5.29	0.00	6.49	10.26	0.00	13.98	6.77	3.45	8.62	3.23	1.37	5.01
Infections of the heart	1.68	1.38	2.01	16.83	9.88	21.99	5.87	1.20	9.06	2.90	0.94	3.50	2.60	0.00	3.18	4.52	0.00	8.72	0.85	0.00	0.98	5.00	3.09	5.88
Peripheral arterial disease	1.68	0.60	3.58	12.85	1.02	21.04	4.43	2.95	4.63	0.47	0.00	1.05	0.00	0.00	0.00	1.07	0.00	2.25	1.05	0.53	1.48	0.44	0.14	0.45
Female infertility	1.50	1.44	1.68	1.10	0.33	1.88	2.74	1.75	3.76	0.50	0.00	1.01	2.27	0.00	3.45	3.13	0.00	4.19	1.29	0.68	1.52	1.77	1.04	2.41
Cancer metastasis (lymph nodes)	1.49	0.26	2.25	32.32	0.69	60.21	4.35	1.43	4.81	0.00	0.00	0.00	51.07	0.00	85.64	5.31	0.00	8.53	32.66	24.32	45.22	39.05	26.47	51.04
Cancer (leukaemia)	1.43	0.46	1.66	834.87	752.18	1035.39	5.92	3.24	6.54	5.22	0.00	11.53	0.00	0.00	0.00	0.00	0.00	0.00	3.76	0.87	4.71	0.00	0.00	0.00
Pneumothorax	1.38	0.96	2.09	5.08	1.93	6.79	5.40	0.66	5.39	1.10	0.39	1.45	0.00	0.00	5.56	2.25	0.00	3.99	0.22	0.00	0.23	0.66	0.00	1.19
Acute kidney injury	1.38	1.26	1.83	18.43	11.56	21.56	6.44	3.31	7.20	7.92	0.97	11.95	0.00	0.00	0.00	9.61	0.00	14.93	2.81	1.32	2.96	3.13	1.48	4.39
Cancer (brain, other cns and intracranial)	1.35	0.16	1.43	3.59	0.33	4.40	0.00	0.00	0.00	568.17	424.61	633.25	54.36	0.00	126.54	5.65	0.00	8.51	86.13	45.31	138.71	1.61	0.26	1.69
Pulmonary embolism	1.32	1.00	1.51	5.81	2.39	7.14	1.34	0.53	1.36	1.40	0.63	1.67	0.00	0.00	0.00	0.00	0.00	0.00	5.72	0.78	8.43	2.56	1.70	3.58
Mycoses	1.28	1.05	1.35	27.36	13.48	35.81	3.60	1.94	4.66	2.90	0.94	3.50	3.78	0.00	5.37	6.72	0.00	10.69	0.85	0.00	0.98	5.45	4.12	7.30
Portal hypertension	1.28	0.94	1.57	7.97	4.96	8.98	2.73	1.40	4.10	2.21	0.60	3.03	0.00	0.00	0.00	4.43	0.00	6.37	1.10	0.65	1.11	2.59	1.80	3.60
Raynaud's syndrome	1.26	1.11	1.48	6.54	0.41	10.28	1.45	0.74	1.52	1.05	0.41	1.29	0.00	0.00	0.00	0.00	0.00	0.00	0.81	0.30	1.34	0.92	0.52	1.00
Cancer (other skin and subcutaneous tissue)	1.19	0.56	1.24	6.48	0.00	6.61	2.67	0.58	3.37	10.18	3.23	16.65	1.24	0.00	2.46	0.00	0.00	0.00	0.00	0.00	0.00	63.35	31.75	83.73
Sleep apnoea	1.12	0.82	1.36	1.57	0.46	2.48	2.35	1.22	2.57	3.91	2.22	5.91	1.80	0.00	3.71	2.42	0.00	3.72	5.67	2.71	11.03	1.44	0.76	1.92
Male infertility	1.12	1.04	1.28	1.60	0.44	2.19	3.06	1.93	4.02	0.50	0.00	1.01	4.33	0.00	7.07	1.88	0.00	3.43	3.13	1.75	5.35	0.95	0.50	1.06
Cancer (testicular)	1.09	0.77	1.59	0.00	0.00	0.00	0.62	0.31	0.84	0.00	0.00	0.00	0.00	0.00	0.00	2.11	0.00	4.26	107.43	61.31	137.15	0.27	0.00	0.54
Eye infections	1.05	0.90	1.35	9.70	4.95	10.31	2.83	1.36	2.92	1.65	0.40	2.52	4.65	0.00	7.11	1.12	0.00	2.10	4.22	0.90	6.89	2.20	1.15	2.42
Bell's palsy	1.05	0.70	1.23	5.45	0.32	9.58	0.23	0.00	0.24	10.18	5.40	12.14	1.37	0.00	2.64	11.80	6.73	17.60	1.46	0.24	2.81	0.93	0.37	1.25
Cancer (colorectal and anus)	1.04	0.56	1.77	31.36	0.00	50.65	8.37	0.54	9.06	0.00	0.00	0.00	18.66	0.00	48.07	0.00	0.00	0.00	3.63	0.80	6.53	7.75	3.65	8.68
End stage renal disease	1.04	0.82	1.15	42.76	32.47	48.63	16.41	12.95	18.14	18.97	9.92	23.79	10.05	4.07	16.31	12.11	3.64	13.35	13.61	7.14	18.29	3.93	2.82	4.99
Hypertrophic cardiomyopathy	0.99	0.48	1.30	156.75	14.14	235.05	23.07	6.03	34.61	2.48	0.41	4.27	3.15	0.00	6.67	1.07	0.00	2.48	0.00	0.00	0.00	0.57	0.00	1.08
Pleural effusion	0.97	0.83	1.28	7.80	4.25	8.73	7.42	5.13	11.36	5.14	2.83	6.48	7.59	1.89	9.64	11.80	2.68	21.42	1.30	0.71	1.80	14.19	4.47	25.07
Myocardial infarction	0.96	0.65	1.00	31.01	3.43	38.90	25.42	14.62	27.20	3.74	0.00	7.05	2.05	0.00	4.05	6.81	0.00	9.39	12.84	5.35	15.23	2.66	0.82	3.78
Dilated cardiomyopathy	0.95	0.31	1.28	156.67	14.14	235.05	18.83	2.09	27.63	2.48	0.41	4.27	3.15	0.00	6.67	1.07	0.00	2.48	0.00	0.00	0.00	0.57	0.00	1.08
Alcoholic liver disease	0.94	0.75	1.25	3.35	1.50	5.15	2.25	0.90	3.24	0.90	0.38	1.05	1.13	0.00	2.46	0.00	0.00	0.00	0.21	0.00	0.21	0.81	0.25	0.87
Right bundle branch block	0.93	0.65	1.21	1.76	0.80	1.78	0.49	0.00	0.51	19.97	1.08	22.27	0.00	0.00	0.00	0.98	0.00	1.82	0.24	0.00	0.24	0.97	0.53	1.42
Bifascicular block	0.92	0.64	1.21	1.76	0.80	1.78	0.49	0.00	0.51	19.97	1.08	22.27	0.00	0.00	0.00	0.98	0.00	1.82	0.24	0.00	0.24	0.97	0.53	1.42
Supraventricular tachycardia	0.91	0.75	1.10	12.50	1.84	18.16	0.49	0.00	0.53	2.83	0.50	3.76	2.15	0.00	4.36	2.81	0.00	4.99	0.00	0.00	0.00	1.47	0.25	2.42
Oesophageal varices	0.88	0.10	2.31	31.88	4.45	40.12	3.72	0.00	3.98	0.00	0.00	0.00	0.00	0.00	0.00	1.81	0.00	4.35	0.00	0.00	0.00	0.00	0.00	0.00
Ventricular tachycardia	0.87	0.68	1.19	12.50	1.84	18.16	0.49	0.00	0.53	2.83	0.50	3.76	2.15	0.00	4.36	2.81	0.00	4.99	0.00	0.00	0.00	1.47	0.25	2.42
Glomerulonephritis	0.86	0.50	1.36	73.11	3.38	75.19	0.00	0.00	2.08	0.00	0.00	0.00	0.00	0.00	0.00	0.00	0.00	0.00	0.21	0.00	0.21	0.36	0.00	0.67
Barrett's oesophagus	0.85	0.73	1.08	3.52	1.90	5.54	5.48	2.79	9.89	0.42	0.00	0.90	5.80	0.00	11.16	0.00	0.00	0.00	0.88	0.47	0.93	0.89	0.32	1.33
Obstructive and reflux uropathy	0.84	0.66	1.12	4.68	1.92	5.41	6.40	3.23	7.44	0.60	0.00	1.21	18.64	4.63	33.51	0.00	0.00	0.00	6.21	3.32	6.70	5.82	3.02	7.34
Infection of anal and rectal regions	0.81	0.69	1.01	1.37	0.34	1.97	10.37	5.23	13.64	0.40	0.00	0.87	2.05	0.00	4.24	0.00	0.00	0.00	0.26	0.00	0.27	0.00	0.00	0.00
Disorders of autonomic nervous system	0.80	0.48	1.04	0.74	0.00	1.60	0.54	0.24	0.69	1.52	0.42	2.27	4.51	1.22	8.30	2.05	0.00	3.01	2.80	0.92	3.02	1.98	1.44	2.36
Nasal polyp	0.80	0.68	0.97	1.29	0.51	1.47	0.87	0.50	0.93	5.07	0.39	7.01	2.86	0.00	5.71	0.00	0.00	0.00	0.66	0.26	0.82	0.82	0.53	0.86
Trifascicular block	0.80	0.40	1.20	0.96	0.00	2.18	0.49	0.00	0.51	19.97	1.08	22.27	0.00	0.00	0.00	0.98	0.00	1.82	0.24	0.00	0.24	0.55	0.27	0.78
Nonrheumatic aortic valve disorders	0.77	0.33	1.16	16.83	0.49	18.67	2.98	0.29	5.74	0.00	0.00	0.00	0.00	0.00	0.00	0.00	0.00	0.00	0.00	0.00	0.00	0.00	0.00	0.00
Anterior and intermediate uveitis	0.75	0.39	0.86	1.22	0.38	1.57	0.33	0.00	0.35	0.00	0.00	0.00	0.00	0.00	0.00	0.00	0.00	0.00	0.00	0.00	0.00	0.00	0.00	0.00
Pulmonary collapse (excluding pneumothorax)	0.73	0.56	1.02	10.31	6.36	13.11	3.60	1.86	4.38	3.61	1.90	5.35	6.11	0.00	11.42	6.24	1.14	10.38	1.81	1.05	2.10	1.19	0.68	1.34
Stable angina	0.73	0.34	1.13	7.00	0.37	11.11	3.85	1.73	3.69	3.74	0.00	7.05	1.61	0.00	4.14	0.00	0.00	0.00	1.48	0.90	1.56	0.00	0.00	0.00
Stroke(not otherwise specified)	0.69	0.32	0.93	14.37	0.00	29.04	0.75	0.00	0.79	14.86	3.88	20.21	0.00	0.00	0.00	0.00	0.00	0.00	15.20	4.81	18.57	2.54	0.00	4.74
Atrial fibrillation	0.67	0.45	0.87	1.77	0.39	2.53	0.49	0.00	0.53	3.74	0.00	4.74	1.80	0.00	3.71	2.68	0.00	4.93	0.26	0.00	0.27	1.35	0.00	2.30
Endometrial hyperplasia and hypertrophy	0.66	0.59	0.90	3.58	0.83	5.21	0.77	0.48	0.83	0.43	0.00	0.82	2.71	0.00	3.33	1.23	0.00	2.21	0.46	0.22	0.68	1.93	1.10	2.01
Cancer (non-hodgkin lymphoma)	0.66	0.01	0.66	4.05	1.09	6.21	190.30	144.86	231.20	0.88	0.00	1.92	0.00	0.00	0.00	0.00	0.00	0.00	1.79	0.00	1.89	0.00	0.00	0.00
Multiple valve disease	0.65	0.28	1.14	4.81	1.05	6.41	10.48	3.26	11.75	0.00	0.00	1.57	0.00	0.00	0.00	0.00	0.00	0.00	0.00	0.00	0.00	0.00	0.00	0.00
Cancer metastasis (brain, cns and intracranial)	0.64	0.12	0.74	2.58	0.32	4.31	0.00	0.00	0.00	27.47	14.99	42.16	55.07	24.38	79.42	93.64	0.00	255.68	17.32	10.54	21.11	74.24	43.00	108.90
Cancer metastasis (bone)	0.64	0.12	0.74	2.58	0.32	4.31	0.00	0.00	0.00	27.47	14.99	42.16	55.07	24.38	79.42	96.20	0.00	262.82	17.32	10.54	21.11	74.35	47.37	91.43
Cancer metastasis (adrenal gland)	0.64	0.12	0.74	2.58	0.32	4.31	0.00	0.00	0.00	27.47	14.99	42.16	55.07	24.38	79.42	93.64	0.00	255.68	17.32	10.54	21.11	73.08	46.53	88.87
Crohn's disease	0.63	0.45	0.79	0.32	0.00	0.64	0.00	0.00	0.00	0.51	0.00	1.17	5.54	0.00	9.68	1.53	0.00	3.37	0.77	0.38	1.11	1.18	0.67	1.52
Cataract	0.61	0.31	0.62	29.09	20.53	41.02	2.94	1.93	3.04	1.07	0.44	1.20	0.00	0.00	0.00	1.81	0.00	3.43	0.00	0.00	0.00	0.20	0.00	0.40
Cancer (cervical)	0.61	0.37	0.89	0.00	0.00	0.00	0.50	0.00	0.53	0.00	0.00	0.00	0.00	0.00	0.00	0.00	0.00	0.00	0.00	0.00	0.00	2.10	1.24	2.45
Thrombophilia	0.61	0.48	0.64	0.85	0.00	1.85	1.17	0.44	1.41	0.81	0.00	1.57	0.00	0.00	0.00	0.00	0.00	0.00	1.39	0.91	1.50	2.23	0.85	2.70
Encephalitis	0.60	0.34	0.61	8.92	5.45	9.66	2.83	1.36	2.92	2.08	0.76	2.42	2.86	0.00	4.65	1.12	0.00	2.10	3.75	0.45	4.75	1.60	0.72	2.04
Agranulocytosis	0.59	0.33	0.70	26.17	18.63	29.15	16.02	11.37	18.69	1.86	0.61	3.09	0.00	0.00	0.00	5.90	0.00	8.11	3.57	0.25	6.70	2.74	1.67	3.31
Subarachnoid haemorrhage	0.57	0.30	0.94	0.00	0.00	0.00	0.51	0.00	0.54	13.10	4.58	14.86	0.00	0.00	0.00	0.00	0.00	0.00	8.06	0.91	8.39	2.54	0.00	4.74
Transient ischaemic attack	0.56	0.31	0.68	8.89	3.41	8.86	0.89	0.25	1.16	5.31	1.89	6.17	0.00	0.00	0.00	0.00	0.00	0.00	1.42	0.88	1.53	0.37	0.17	0.53
Rheumatoid arthritis	0.54	0.43	0.70	5.84	2.02	6.58	2.80	1.38	3.23	0.49	0.00	0.95	1.40	0.00	2.96	5.90	0.00	10.45	0.73	0.00	0.82	1.20	0.38	1.40
Liver fibrosis sclerosis and cirrhosis	0.54	0.33	0.71	4.43	0.90	6.25	2.34	1.05	2.74	0.00	0.00	0.00	0.00	0.00	0.00	0.00	0.00	0.00	1.82	0.21	3.48	1.06	0.60	1.12
Ischaemic stroke	0.53	0.14	0.71	0.85	0.00	1.85	0.99	0.00	1.03	20.18	11.42	23.53	0.00	0.00	0.00	0.00	0.00	0.00	14.09	8.32	21.19	2.79	0.27	5.10
Left bundle branch block	0.49	0.35	0.57	0.52	0.00	1.04	1.86	0.55	3.39	1.27	0.00	2.90	0.00	0.00	0.00	0.00	0.00	0.00	0.00	0.00	0.00	0.63	0.00	1.13
Scoliosis	0.49	0.34	0.66	1.48	0.52	1.83	1.02	0.58	1.06	4.62	2.78	5.53	0.00	0.00	0.00	4.43	0.00	7.12	0.00	0.00	0.00	0.31	0.12	0.43
Infection of liver	0.49	0.31	0.74	13.48	3.61	17.59	4.70	2.19	5.52	2.03	0.41	3.59	0.00	0.00	0.00	1.12	0.00	2.10	0.75	0.39	1.13	1.71	0.78	2.15
Atrioventricular block, second degree	0.49	0.15	0.92	0.52	0.00	1.04	0.99	0.47	1.47	1.27	0.00	2.90	0.00	0.00	0.00	0.00	0.00	0.00	0.00	0.00	0.00	0.63	0.00	1.13
Atrioventricular block, first degree	0.49	0.15	0.92	0.52	0.00	1.04	0.99	0.47	1.47	1.27	0.00	2.90	0.00	0.00	0.00	0.00	0.00	0.00	0.00	0.00	0.00	0.63	0.00	1.13
Atrioventricular block, complete	0.49	0.15	0.92	0.52	0.00	1.04	0.99	0.47	1.47	1.27	0.00	2.90	0.00	0.00	0.00	0.00	0.00	0.00	0.00	0.00	0.00	0.63	0.00	1.13
Infection of bones and joints	0.48	0.25	0.56	0.41	0.00	0.85	2.15	0.42	3.89	0.00	0.00	3.28	15.89	0.00	30.30	84.53	0.00	146.74	5.28	0.77	5.68	0.79	0.48	0.89
Cancer (thyroid)	0.47	0.03	0.53	3.04	0.94	3.94	0.00	0.00	0.00	1.02	0.00	2.08	0.00	0.00	0.00	0.00	0.00	0.00	0.99	0.32	1.29	49.12	30.65	68.71
Glaucoma	0.46	0.16	0.55	0.34	0.00	0.67	0.00	0.00	0.00	0.00	0.00	0.00	0.00	0.00	0.00	0.00	0.00	0.00	0.00	0.00	0.00	0.68	0.00	1.23
Secondary or other thrombocytopaenia	0.45	0.32	0.60	9.77	6.61	11.37	5.40	3.02	7.63	2.23	0.76	3.01	1.10	0.00	2.59	0.00	0.00	0.00	0.94	0.45	1.37	1.68	0.72	2.31
Enteropathic arthropathy	0.44	0.35	0.67	0.34	0.00	0.68	1.22	0.28	2.03	1.06	0.43	1.19	0.00	0.00	0.00	0.00	0.00	0.00	0.00	0.00	0.00	0.00	0.00	0.00
Non-acute cystitis	0.40	0.29	0.46	0.83	0.33	0.89	0.40	0.00	0.44	0.00	0.00	0.00	3.38	0.00	4.96	1.07	0.00	2.22	0.00	0.00	0.00	1.71	0.54	1.93
Sick sinus syndrome	0.38	0.25	0.50	0.00	0.00	0.00	1.05	0.52	1.51	0.63	0.00	1.21	0.00	0.00	0.00	0.00	0.00	0.00	1.03	0.32	1.35	5.80	0.44	6.14
Essential tremor	0.36	0.21	0.44	0.00	0.00	0.00	5.13	0.00	5.67	1.06	0.00	2.29	1.10	0.00	2.03	0.00	0.00	0.00	0.00	0.00	0.00	0.00	0.00	0.00
Primary or idiopathic thrombocytopaenia	0.34	0.31	0.42	8.94	5.97	9.83	4.58	2.74	5.39	1.64	0.43	2.01	1.10	0.00	2.59	0.00	0.00	0.00	0.73	0.28	0.94	1.68	0.72	2.31
Respiratory failure	0.33	0.21	0.42	13.86	7.05	17.31	1.10	0.64	1.16	2.99	1.04	3.90	4.78	0.00	9.48	18.48	10.87	25.15	0.75	0.46	0.80	0.25	0.00	0.49
Spinal stenosis	0.32	0.22	0.40	13.39	0.00	27.60	1.88	0.60	2.05	0.60	0.00	1.21	4.08	0.00	8.45	0.00	0.00	0.00	0.00	0.00	0.00	0.00	0.00	0.00
Multiple sclerosis	0.31	0.21	0.57	0.00	0.00	0.00	2.09	0.00	2.45	0.49	0.00	0.98	0.00	0.00	0.00	1.08	0.00	2.08	0.36	0.00	0.39	0.57	0.15	0.78
Pericardial effusion (noninflammatory)	0.31	0.20	0.47	5.19	3.19	7.28	4.23	1.35	5.43	0.00	0.00	0.00	0.00	0.00	0.00	1.96	0.00	3.65	0.19	0.00	0.20	8.01	1.30	14.13
Intracerebral haemorrhage	0.31	0.08	0.47	2.07	0.47	2.71	0.51	0.00	0.54	9.29	4.73	12.14	0.00	0.00	0.00	0.00	0.00	0.00	7.34	0.22	7.60	2.54	0.00	4.74
Cancer (malignant melanoma)	0.30	0.08	0.47	2.47	0.00	4.91	0.79	0.00	0.80	0.00	0.00	0.00	0.00	0.00	0.00	0.00	0.00	0.00	2.72	1.17	3.68	59.07	45.35	74.92
Osteoporosis	0.29	0.21	0.40	5.68	3.78	7.23	1.50	0.87	1.64	1.37	0.44	2.24	2.72	0.00	4.04	1.06	0.00	2.22	0.41	0.21	0.63	1.54	0.62	1.84
Lupus erythematosus (local and systemic)	0.29	0.10	0.28	0.61	0.00	1.27	0.78	0.49	0.82	0.00	0.00	0.00	0.00	0.00	0.00	1.81	0.00	3.25	0.41	0.00	0.45	0.18	0.00	0.34
Unstable angina	0.26	0.13	0.40	7.00	0.37	11.11	1.92	1.05	2.44	0.00	0.00	0.00	1.61	0.00	4.14	0.00	0.00	0.00	1.04	0.55	1.11	0.00	0.00	0.00
Autoimmune liver disease	0.24	0.09	0.34	4.76	1.41	9.27	0.97	0.24	1.34	1.23	0.40	1.67	0.00	0.00	0.00	0.00	0.00	0.00	0.00	0.00	0.00	0.54	0.33	0.56
Cancer (other organs)	0.23	0.08	0.40	0.41	0.00	0.82	6.85	4.10	10.63	3.70	0.46	4.55	19.04	2.33	27.51	73.93	30.83	129.91	13.32	9.00	14.48	7.55	4.43	10.96
Hyperparathyroidism	0.17	0.04	0.23	79.89	0.52	78.77	1.01	0.31	1.35	18.69	6.32	27.01	0.00	0.00	0.00	303.46	1.26	436.53	2.89	0.45	5.29	0.00	0.00	0.00
Macular degeneration	0.16	0.10	0.23	2.74	0.77	3.60	0.00	0.00	0.00	1.02	0.42	1.30	1.02	0.00	2.20	5.76	0.00	7.48	0.86	0.00	0.93	0.93	0.42	1.29
Ankylosing spondylitis	0.16	0.05	0.17	0.00	0.00	0.00	0.39	0.00	0.43	0.00	0.00	0.00	0.00	0.00	0.00	0.00	0.00	0.00	0.00	0.00	0.00	0.00	0.00	0.00
Secondary polycythaemia	0.14	0.07	0.29	1.23	0.37	1.75	0.26	0.00	0.27	0.00	0.00	0.00	0.00	0.00	0.00	0.98	0.00	1.93	0.64	0.24	1.06	0.56	0.26	0.87
Abdominal aortic aneurysm	0.14	0.08	0.30	6.41	0.00	13.67	0.57	0.25	0.87	0.00	0.00	0.00	0.00	0.00	0.00	0.00	0.00	0.00	0.00	0.00	0.00	0.00	0.00	0.00
Aplastic anaemias	0.13	0.07	0.22	5.73	2.82	6.60	0.26	0.00	0.27	0.88	0.41	0.88	0.00	0.00	0.00	0.00	0.00	0.00	0.00	0.00	0.00	0.97	0.40	1.37
Cancer (stomach)	0.13	0.00	0.25	0.00	0.00	0.00	1.97	0.00	2.14	0.00	0.00	0.00	0.00	0.00	0.00	0.00	0.00	0.00	0.22	0.00	0.23	0.00	0.00	0.00
Primary pulmonary hypertension	0.11	0.06	0.15	0.00	0.00	0.00	0.00	0.00	0.00	0.00	0.00	0.00	0.00	0.00	0.00	0.00	0.00	0.00	1.89	0.42	2.25	0.00	0.00	0.00
Secondary pulmonary hypertension	0.09	0.06	0.13	0.00	0.00	0.00	0.00	0.00	0.00	0.00	0.00	0.00	0.00	0.00	0.00	0.00	0.00	0.00	1.89	0.42	2.25	0.00	0.00	0.00
Angiodysplasia of colon	0.09	0.05	0.15	0.00	0.00	1.03	0.33	0.00	0.34	0.00	0.00	0.00	0.00	0.00	0.00	0.00	0.00	0.00	0.00	0.00	0.00	0.00	0.00	0.00
Bronchiectasis	0.09	0.05	0.13	5.08	2.32	5.60	2.78	1.16	3.21	0.51	0.00	1.15	0.00	0.00	0.00	0.00	0.00	0.00	0.56	0.27	0.84	0.42	0.00	0.74
Dementia	0.08	0.03	0.09	0.40	0.00	0.81	0.28	0.00	0.30	0.40	0.00	0.84	0.00	0.00	0.00	0.00	0.00	0.00	0.00	0.00	0.00	0.20	0.00	0.37
Other haemolytic anaemias	0.07	0.03	0.11	1.18	0.33	2.01	1.67	0.85	1.85	0.00	0.00	0.00	0.00	0.00	0.00	0.00	0.00	0.00	0.00	0.00	0.00	0.00	0.00	0.00
Systemic sclerosis	0.07	0.00	0.11	0.80	0.37	0.79	0.94	0.00	1.06	0.50	0.00	1.02	0.00	0.00	0.00	0.00	0.00	0.00	0.00	0.00	0.00	0.00	0.00	0.00
MGUS	0.05	0.00	0.10	1.75	0.38	2.05	1.16	0.56	1.19	0.00	0.00	0.00	0.00	0.00	0.00	0.00	0.00	0.00	0.19	0.00	0.20	0.38	0.15	0.53
Immunodeficiencies	0.05	0.01	0.06	0.72	0.00	0.74	2.15	0.95	2.18	0.00	0.00	0.00	0.00	0.00	0.00	0.00	0.00	0.00	0.00	0.00	0.00	1.35	0.00	2.20
Cancer (hodgkin lymphoma)	0.04	0.00	0.07	0.00	0.00	0.00	413.42	391.89	499.02	0.00	0.00	0.00	0.00	0.00	0.00	0.00	0.00	0.00	6.05	0.00	6.36	3.76	0.00	7.38
Cancer (ovarian)	0.03	0.01	0.04	0.00	0.00	0.00	0.49	0.00	0.55	0.00	0.00	0.00	0.00	0.00	0.00	0.00	0.00	0.00	31.72	1.87	33.49	2.70	0.00	4.44
Cancer (myelodysplastic syndromes)	0.03	0.00	0.05	109.00	50.97	139.01	0.52	0.00	0.55	0.00	0.00	0.00	0.00	0.00	0.00	0.00	0.00	0.00	0.00	0.00	0.00	0.00	0.00	0.00
Posterior uveitis	0.02	0.00	0.05	0.39	0.00	0.81	0.00	0.00	0.00	0.00	0.00	0.00	0.00	0.00	0.00	0.00	0.00	0.00	0.00	0.00	0.00	0.48	0.00	0.96
Cancer (bone and articular cartilage)	0.02	0.00	0.02	9.19	3.56	11.22	0.44	0.00	0.51	99.52	2.08	178.84	429.99	273.80	635.42	27.49	7.30	42.59	1.10	0.00	1.21	0.00	0.00	0.00
Cancer (oro-pharyngeal)	0.02	0.00	0.04	0.00	0.00	0.00	11.70	0.00	16.23	0.00	0.00	0.00	0.00	0.00	0.00	0.00	0.00	0.00	0.00	0.00	0.00	9.30	5.44	13.29
Other interstitial pulmonary diseases with fibrosis	0.02	0.00	0.03	12.31	1.31	25.45	1.89	0.54	2.24	5.24	0.00	10.29	0.00	0.00	0.00	0.00	0.00	0.00	0.41	0.00	0.46	0.00	0.00	0.00
Cancer (lung and trachea)	0.01	0.00	0.02	12.32	0.00	18.73	2.75	0.00	3.00	0.00	0.00	0.00	4.44	0.00	5.88	0.00	0.00	0.00	0.19	0.00	0.20	0.36	0.00	0.68
Cancer (bladder)	0.01	0.00	0.01	0.00	0.00	4.10	0.00	0.00	0.00	0.00	0.00	0.00	0.00	0.00	0.00	0.00	0.00	0.00	0.00	0.00	0.00	0.89	0.36	1.40
Cancer (uterine	0.00	0.00	0.00	0.00	0.00	0.00	0.00	0.00	0.00	0.00	0.00	0.00	0.00	0.00	0.00	0.00	0.00	0.00	0.26	0.00	0.28	0.00	0.00	0.00
Cancer (pancreatic)	0.00	0.00	0.00	0.00	0.00	0.00	0.00	0.00	0.00	0.00	0.00	0.00	0.00	0.00	0.00	1.23	0.00	2.29	0.00	0.00	0.00	13.55	0.32	25.42
Cancer (oesophageal)	0.00	0.00	0.00	0.00	0.00	0.00	3.15	1.40	4.56	0.00	0.00	0.00	0.00	0.00	0.00	0.00	0.00	0.00	0.00	0.00	0.00	0.00	0.00	0.00
Cancer (multiple myeloma and malignant plasma cell neoplasms)	0.00	0.00	0.00	2.25	0.79	3.06	0.00	0.00	0.00	0.00	0.00	0.00	0.00	0.00	0.00	0.00	0.00	0.00	0.00	0.00	0.00	0.00	0.00	0.00
Cancer (multiple independent sites)	0.00	0.00	0.00	0.00	0.00	0.00	0.00	0.00	0.00	0.00	0.00	0.00	0.00	0.00	0.00	0.00	0.00	0.00	0.36	0.00	0.40	2.96	1.47	3.46
Cancer (liver)	0.00	0.00	0.00	0.00	0.00	0.00	0.00	0.00	0.00	0.00	0.00	0.00	0.00	0.00	0.00	0.00	0.00	0.00	0.00	0.00	0.00	0.00	0.00	0.00
Cancer (kidney and ureter)	0.00	0.00	0.00	0.00	0.00	0.00	5.11	0.00	5.65	0.00	0.00	0.00	14.66	0.00	21.61	2.68	0.00	4.57	0.00	0.00	0.00	0.00	0.00	0.00
Cancer (biliary tract)	0.00	0.00	0.00	0.00	0.00	0.00	0.00	0.00	0.00	0.00	0.00	0.00	0.00	0.00	0.00	0.00	0.00	0.00	0.00	0.00	0.00	0.00	0.00	0.00

Table S17. Cumulative burden of 183 condition-specific outcomes in cancer survivors and controls by indices of multiple deprivation (IMD) status at 45 years attained age. Cumulative burden per 100 individuals is shown.

Health conditions	Controls						All cancer survivors					
	IMD 1 (least deprived)			IMD 5 (most deprived)			IMD 1 (least deprived)			IMD 5 (most deprived)		
	Cumulative burden	Lower CI	Upper CI	Cumulative burden	Lower CI	Upper CI	Cumulative burden	Lower CI	Upper CI	Cumulative burden	Lower CI	Upper CI
Mental health	173.53	140.29	202.11	371.36	319.50	412.25	309.99	231.21	333.99	414.11	361.57	433.51
Bacterial infections	21.29	18.77	20.92	39.41	34.02	41.87	71.34	66.89	100.73	89.02	69.76	94.13
Hypertension	23.25	15.38	27.16	35.39	23.80	44.20	46.14	17.61	47.27	37.04	17.10	53.15
Migraine	21.18	16.81	23.03	25.46	20.77	28.13	26.67	22.90	32.45	33.58	24.84	38.07
Hypo or hyperthyroidism	7.03	3.78	8.65	36.15	16.96	50.04	98.89	72.51	123.85	183.18	136.48	222.58
Menorrhagia and polymenorrhoea	14.41	11.63	16.81	20.62	17.49	23.13	11.41	7.12	12.93	27.90	19.19	36.84
Asthma	12.42	11.31	12.68	16.73	15.37	16.86	21.78	19.63	23.94	30.89	26.26	34.06
Oesophagitis and oesophageal ulcer	9.76	8.51	11.31	15.11	12.01	15.45	12.48	10.29	16.33	24.77	14.28	27.29
Diabetic ophthalmic complications	7.67	4.05	11.90	14.61	10.16	15.98	45.44	12.14	53.32	40.00	24.56	58.30
Obesity	7.71	6.61	8.18	16.48	14.06	16.75	12.41	9.58	15.31	24.33	22.62	27.35
Infections of other or unspecified organs	7.28	5.89	7.97	12.23	10.35	13.60	31.95	20.99	39.61	38.10	34.37	42.18
Enthesopathies and synovial disorders	9.39	7.87	9.82	9.29	7.94	10.63	12.58	9.92	13.75	9.03	6.52	10.68
Urolithiasis	2.92	2.27	3.73	8.45	4.98	10.11	5.62	2.81	6.76	10.61	5.44	18.42
Diabetes	7.90	6.24	9.01	12.29	10.20	12.88	8.09	6.38	9.63	20.81	16.66	27.33
Diabetic neurological complications	6.41	2.42	8.90	11.02	6.87	13.82	45.73	14.97	51.77	37.45	20.42	57.26
Infection of skin and subcutaneous tissues	6.17	5.34	6.60	13.20	11.86	13.65	12.45	9.96	16.45	21.35	17.69	25.19
Ear and upper respiratory tract infections	6.39	5.08	7.39	10.57	9.72	10.92	10.11	7.26	11.87	24.53	18.05	28.83
Cholelithiasis	8.17	5.99	9.15	8.89	7.16	9.65	4.98	2.90	6.20	11.08	7.16	14.53
HIV	6.02	4.93	6.43	6.95	5.85	7.63	3.30	1.91	4.39	11.98	7.34	13.86
Lower respiratory tract infections	6.64	3.24	8.12	9.68	8.07	9.93	33.68	22.45	36.15	36.90	29.96	44.86
Gastrooesophageal reflux disease	4.93	4.10	5.49	7.88	6.93	8.14	7.43	5.22	10.24	13.40	11.25	16.67
Coronary heart disease not otherwise specified	4.92	4.05	6.03	8.47	7.03	9.58	9.16	4.87	12.96	5.88	3.31	6.33
Dysmenorrhoea	6.31	5.73	7.55	8.77	5.90	9.83	9.27	5.83	12.59	12.15	6.85	15.74
Urinary incontinence	4.70	3.14	6.56	9.02	5.97	9.81	11.09	8.85	13.21	22.55	16.38	32.15
Infections of the digestive system	4.84	4.00	5.58	7.67	5.39	8.24	26.62	17.47	34.05	17.23	14.07	24.11
Urinary tract infections	7.56	4.00	14.99	6.60	5.47	7.25	22.24	18.56	31.51	26.72	17.01	37.95
Peritonitis	4.94	4.04	6.39	8.81	6.63	10.49	15.71	12.81	17.84	15.30	10.24	20.69
Cholecystitis	4.19	2.73	4.63	6.66	5.56	7.29	5.33	3.00	6.54	9.08	7.39	11.17
Endometriosis	4.00	2.60	5.65	6.17	2.59	7.30	2.06	1.02	2.26	4.08	1.58	4.72
Ulcerative colitis	6.27	4.71	8.02	4.33	2.55	4.74	23.14	9.93	26.74	0.00	0.00	0.00
Cholangitis	4.32	3.91	4.98	6.58	5.76	7.06	6.34	3.87	9.13	7.95	5.61	9.42
Cancer (breast)	0.11	0.00	0.21	5.03	0.00	5.72	15.76	3.20	19.28	24.09	9.86	30.61
Chronic sinusitis	4.27	3.49	4.51	4.45	3.73	5.05	3.93	2.66	5.37	7.01	5.02	9.59
Polycystic ovarian syndrome	2.81	2.23	3.39	9.42	6.71	11.55	1.70	0.97	1.91	8.16	4.94	8.29
Gastritis and duodenitis	2.95	2.51	3.15	6.29	5.51	6.53	8.39	5.70	10.93	11.66	9.38	12.84
Anorectal fistula	3.77	2.95	3.72	7.11	5.85	8.77	4.50	2.55	4.94	4.53	3.13	7.30
Postviral fatigue syndrome, neurasthenia and fibromyalgia	2.89	2.05	3.06	6.28	5.05	7.27	8.27	6.74	10.21	10.48	9.12	12.51
Hepatic failure	3.55	1.36	5.13	8.31	3.47	9.49	10.81	8.04	16.22	17.32	13.45	25.89
Viral diseases (excluding chronic hepatitis/HIV)	3.81	3.11	3.91	5.08	3.82	4.88	17.12	9.48	22.78	14.41	11.71	16.02
Chronic viral hepatitis	4.75	4.07	4.99	4.05	3.45	4.56	4.40	3.21	6.49	7.56	5.95	9.28
Pancreatitis	0.08	0.05	0.18	2.69	1.35	3.83	0.56	0.26	0.90	1.81	0.55	1.84
Other anaemias	2.49	2.08	2.48	4.85	4.10	5.60	8.01	6.24	9.67	11.25	10.19	12.21
Venous thromboembolic disease	1.69	0.20	4.12	5.47	4.39	6.06	5.46	2.95	6.76	9.80	7.23	12.41
Osteoarthritis (excluding spine)	2.18	1.56	2.75	2.80	1.99	3.03	1.05	0.46	1.32	9.07	5.85	11.63
Cancer metastasis (other organs)	0.44	0.00	0.89	8.93	1.73	16.23	34.14	22.68	38.59	104.60	77.51	136.80
COPD	1.63	1.07	1.81	4.63	3.91	5.21	2.05	1.06	2.27	6.68	4.42	8.05
Intracranial hypertension	1.51	0.38	1.55	7.07	1.42	16.33	6.02	2.77	10.58	3.98	2.36	5.15
Septicaemia	3.11	2.18	2.97	3.20	2.19	3.29	10.93	8.19	15.07	16.14	12.21	18.88
Diverticular disease of intestine	1.97	1.17	2.84	5.58	3.80	8.54	1.70	0.78	2.04	1.45	0.37	2.46
Coeliac disease	2.51	1.92	3.09	1.75	1.36	1.99	1.64	0.86	1.68	3.55	1.91	4.19
Erectile dysfunction	1.45	1.09	1.66	3.00	2.32	3.02	4.23	2.27	6.02	3.53	2.14	3.61
Epilepsy	1.82	1.34	1.79	2.85	2.24	3.25	8.46	6.62	10.39	14.60	11.70	17.95
Fatty liver	1.90	1.21	2.68	2.49	1.96	3.02	3.87	2.30	5.71	4.58	3.18	5.10
Nonrheumatic mitral valve disorders	0.89	0.36	1.44	0.32	0.07	0.67	2.27	0.47	4.04	2.98	1.62	3.55
Heart failure	3.74	2.22	6.31	1.63	0.73	1.67	11.75	4.40	23.27	16.19	5.50	16.70
Hypertrophy of nasal turbinates	1.47	1.14	1.48	1.74	1.37	1.85	0.66	0.38	0.79	6.12	3.93	7.51
Peripheral neuropathies	1.99	1.26	2.62	1.80	1.01	2.20	3.56	1.59	4.83	3.14	1.59	4.00
Cancer metastasis (retroperitoneum and peritoneum	0.37	0.00	0.75	8.84	1.54	16.13	25.03	10.52	45.03	74.18	41.26	99.07
Cancer metastasis (pleura)	0.37	0.00	0.75	8.84	1.54	16.13	25.03	10.52	45.03	74.18	41.26	99.07
Cancer metastasis (lung)	0.37	0.00	0.75	8.84	1.54	16.13	26.52	12.96	30.49	75.70	44.32	101.31
Cancer metastasis (liver and intrahepatic bile duct)	0.37	0.00	0.75	8.84	1.54	16.13	25.01	10.52	44.96	74.45	41.54	99.07
Cancer metastasis (bowel)	0.37	0.00	0.75	8.84	1.54	16.13	25.03	10.52	45.03	74.18	41.26	99.07
Meningitis	2.02	1.47	2.32	1.56	1.25	1.69	6.99	4.22	8.37	10.47	8.95	12.26
Other cardiomyopathy	1.85	0.04	3.62	2.51	1.08	3.07	7.33	4.08	7.13	40.68	6.58	42.78
Infections of the heart	1.56	1.05	2.03	2.83	1.47	4.27	4.10	1.71	3.98	8.51	4.42	12.08
Peripheral arterial disease	4.05	1.16	6.56	2.16	0.78	2.77	2.78	1.61	3.47	0.81	0.26	1.10
Female infertility	1.49	1.18	1.70	1.92	1.44	2.12	1.39	0.76	1.39	1.62	0.74	1.91
Cancer metastasis (lymph nodes)	0.20	0.05	0.30	6.02	2.88	15.23	26.62	12.09	43.25	41.74	22.80	53.59
Cancer (leukaemia)	1.06	0.00	2.16	0.16	0.00	0.46	71.38	46.03	108.26	151.55	115.23	270.84
Pneumothorax	0.63	0.18	0.90	1.28	0.84	1.87	0.79	0.51	0.78	0.00	0.00	0.00
Acute kidney injury	1.75	0.31	3.52	2.31	0.95	3.53	5.50	2.94	9.43	3.08	1.57	3.37
Cancer (brain, other cns and intracranial)	0.00	0.00	0.00	0.00	0.00	0.00	52.36	33.81	57.87	104.40	76.33	156.80
Pulmonary embolism	0.58	0.16	1.21	2.05	0.85	3.49	4.18	0.92	6.12	2.10	0.72	2.13
Mycoses	1.69	0.99	2.45	1.12	1.01	1.20	4.95	1.53	5.10	9.42	5.86	10.99
Portal hypertension	0.77	0.38	1.04	1.99	1.47	2.98	2.83	1.08	3.58	3.78	2.15	4.91
Raynaud's syndrome	1.42	0.92	1.80	1.42	1.08	1.81	2.20	1.23	2.50	0.27	0.00	0.29
Cancer (other skin and subcutaneous tissue)	1.02	0.17	1.84	0.41	0.17	0.64	8.22	3.85	9.21	95.70	40.47	99.32
Sleep apnoea	0.17	0.07	0.21	1.98	1.77	2.21	1.71	0.83	2.08	4.10	1.78	6.56
Male infertility	1.23	1.00	1.58	1.40	1.25	1.64	3.89	2.31	6.12	1.42	0.77	1.54
Cancer (testicular)	0.22	0.11	0.56	2.13	0.95	2.20	29.94	25.05	54.18	18.75	7.63	33.85
Eye infections	0.66	0.35	0.88	0.82	0.49	1.02	4.62	3.06	6.55	5.02	2.79	6.61
Bell's palsy	1.22	0.42	1.47	0.93	0.68	1.20	4.10	2.66	4.31	1.91	0.95	2.09
Cancer (colorectal and anus)	0.08	0.00	0.16	4.98	2.94	10.46	8.93	2.24	13.09	4.00	0.00	4.18
End stage renal disease	1.66	0.90	1.92	1.25	0.92	1.50	15.72	12.82	18.64	18.32	14.31	20.67
Hypertrophic cardiomyopathy	1.81	0.00	3.61	0.70	0.40	1.37	1.37	0.40	1.74	36.83	18.93	36.35
Pleural effusion	0.47	0.05	0.74	2.35	0.87	3.08	3.08	1.61	3.91	20.09	5.58	38.30
Myocardial infarction	0.00	0.00	0.00	2.26	1.11	2.91	4.71	2.39	4.75	7.96	3.01	9.87
Dilated cardiomyopathy	1.81	0.00	3.61	0.70	0.40	1.37	2.16	0.40	2.63	36.77	18.93	36.29
Alcoholic liver disease	0.52	0.28	0.66	1.48	0.89	2.15	0.77	0.32	0.86	1.04	0.29	1.11
Right bundle branch block	1.12	0.42	1.46	0.79	0.45	1.00	0.48	0.20	0.58	2.63	1.60	3.00
Bifascicular block	1.12	0.42	1.46	0.75	0.43	0.98	0.48	0.20	0.58	2.63	1.60	3.00
Supraventricular tachycardia	1.69	0.44	2.40	0.84	0.29	0.92	1.06	0.00	1.11	1.50	0.92	1.73
Oesophageal varices	0.00	0.00	0.00	3.66	0.04	7.36	0.00	0.00	0.00	11.16	1.40	11.03
Ventricular tachycardia	1.69	0.44	2.40	0.84	0.29	0.92	1.06	0.00	1.11	1.50	0.92	1.73
Glomerulonephritis	0.96	0.26	1.05	0.45	0.14	0.76	0.56	0.00	0.62	0.00	0.00	0.00
Barrett's oesophagus	0.84	0.38	1.01	0.97	0.61	1.21	2.10	0.77	2.27	2.09	0.56	3.87
Obstructive and reflux uropathy	0.72	0.26	0.94	1.10	0.59	1.41	3.47	1.27	3.60	7.65	3.60	10.16
Infection of anal and rectal regions	0.47	0.26	0.52	0.80	0.40	1.08	5.46	0.73	5.46	0.96	0.00	1.00
Disorders of autonomic nervous system	0.60	0.17	1.05	0.99	0.16	1.53	2.18	0.51	2.47	1.53	0.72	1.82
Nasal polyp	0.65	0.29	0.92	0.95	0.45	1.65	0.25	0.00	0.26	2.31	0.96	2.75
Trifascicular block	0.83	0.28	1.24	0.65	0.14	0.85	0.48	0.20	0.58	1.45	0.83	1.97
Nonrheumatic aortic valve disorders	0.13	0.07	0.30	0.71	0.14	1.17	2.67	0.00	2.84	0.00	0.00	0.00
Anterior and intermediate uveitis	1.32	0.30	1.62	0.66	0.08	1.41	0.34	0.00	0.34	0.00	0.00	0.00
Pulmonary collapse (excluding pneumothorax)	0.79	0.22	1.16	0.35	0.11	0.39	3.06	1.90	3.55	3.93	1.74	5.59
Stable angina	1.18	0.10	3.33	1.88	0.62	2.46	0.40	0.00	0.40	1.17	0.31	1.48
Stroke(not otherwise specified)	0.15	0.04	0.22	1.77	0.53	2.54	4.92	1.95	5.47	11.10	7.20	12.87
Atrial fibrillation	0.54	0.08	0.70	0.73	0.31	0.79	0.36	0.00	0.38	1.85	0.94	1.83
Endometrial hyperplasia and hypertrophy	0.13	0.07	0.27	0.87	0.43	0.87	0.20	0.00	0.20	1.95	0.88	2.59
Cancer (non-hodgkin lymphoma)	0.05	0.00	0.09	0.00	0.00	0.00	57.46	21.13	70.12	28.09	16.41	35.16
Multiple valve disease	0.11	0.07	0.25	0.92	0.14	1.65	0.32	0.00	0.34	2.14	0.85	3.26
Cancer metastasis (brain, cns and intracranial)	0.37	0.00	0.75	2.46	0.50	3.90	17.41	6.53	31.21	44.58	24.98	52.49
Cancer metastasis (bone)	0.37	0.00	0.75	2.46	0.50	3.90	17.20	10.95	27.36	44.56	24.98	53.46
Cancer metastasis (adrenal gland)	0.37	0.00	0.75	2.46	0.50	3.90	17.20	10.95	27.36	43.82	24.98	51.95
Crohn's disease	0.57	0.31	0.83	0.81	0.33	1.18	1.17	0.28	1.41	0.54	0.00	0.60
Cataract	0.24	0.05	0.39	0.29	0.15	0.40	7.61	4.65	12.35	2.74	1.48	2.90
Cancer (cervical)	0.05	0.00	0.11	0.00	0.00	0.00	0.48	0.00	0.51	1.92	0.83	2.01
Thrombophilia	0.48	0.36	0.54	0.57	0.30	0.66	0.79	0.26	1.07	0.00	0.00	0.00
Encephalitis	0.82	0.38	1.04	0.37	0.09	0.56	3.97	2.15	5.73	3.61	1.86	5.35
Agranulocytosis	0.60	0.07	1.06	0.23	0.07	0.37	13.86	8.40	19.84	7.07	3.86	8.98
Subarachnoid haemorrhage	1.56	0.21	4.47	0.51	0.07	0.95	1.92	0.00	1.98	8.48	4.59	8.81
Transient ischaemic attack	0.31	0.21	0.39	0.85	0.14	1.35	0.28	0.00	0.32	4.25	2.32	4.99
Rheumatoid arthritis	0.20	0.08	0.24	0.66	0.23	0.99	0.20	0.00	0.20	2.36	0.79	2.50
Liver fibrosis sclerosis and cirrhosis	0.45	0.21	1.02	0.94	0.43	1.08	1.48	0.70	1.50	1.60	0.80	2.31
Ischaemic stroke	0.11	0.00	0.21	1.12	0.24	1.79	5.08	3.14	5.79	10.70	5.82	14.43
Left bundle branch block	0.29	0.11	0.34	0.26	0.14	0.33	0.20	0.00	0.20	1.32	0.00	1.54
Scoliosis	0.49	0.29	0.65	0.39	0.18	0.47	0.28	0.00	0.31	1.47	0.75	2.11
Infection of liver	0.72	0.43	0.96	0.35	0.12	0.41	3.12	1.97	3.09	3.81	1.63	4.40
Atrioventricular block, second degree	0.26	0.12	0.29	0.26	0.14	0.33	0.20	0.00	0.20	0.44	0.00	0.51
Atrioventricular block, first degree	0.26	0.12	0.29	0.26	0.14	0.33	0.20	0.00	0.20	0.44	0.00	0.51
Atrioventricular block, complete	0.26	0.12	0.29	0.26	0.14	0.33	0.20	0.00	0.20	0.44	0.00	0.51
Infection of bones and joints	0.10	0.00	0.20	0.13	0.08	0.16	15.58	0.27	30.40	2.04	0.31	3.63
Cancer (thyroid)	0.00	0.00	0.00	0.56	0.37	1.09	3.90	1.97	6.86	10.19	3.21	12.53
Glaucoma	0.31	0.08	0.35	0.05	0.00	0.11	0.00	0.00	0.00	0.00	0.00	0.00
Secondary or other thrombocytopaenia	0.78	0.21	1.18	0.46	0.35	0.61	3.59	2.01	4.48	2.91	1.54	3.55
Enteropathic arthropathy	0.71	0.23	0.90	0.20	0.07	0.32	1.00	0.27	1.76	0.00	0.00	0.00
Non-acute cystitis	0.65	0.33	0.76	0.44	0.17	0.64	0.53	0.25	0.71	2.55	0.68	2.46
Sick sinus syndrome	0.89	0.27	1.22	0.37	0.14	0.85	0.00	0.00	0.00	8.55	0.99	8.77
Essential tremor	0.13	0.07	0.28	0.09	0.04	0.17	0.00	0.00	0.00	0.31	0.00	0.33
Primary or idiopathic thrombocytopaenia	0.47	0.17	0.51	0.34	0.17	0.43	3.59	2.01	4.48	2.11	1.09	2.90
Respiratory failure	0.31	0.08	0.47	0.50	0.15	0.80	3.89	2.00	5.00	3.09	1.73	4.15
Spinal stenosis	0.19	0.04	0.32	1.08	0.41	1.49	0.00	0.00	0.00	0.00	0.00	0.00
Multiple sclerosis	0.14	0.08	0.29	0.63	0.26	0.89	0.24	0.00	0.24	0.00	0.00	0.00
Pericardial effusion (noninflammatory)	0.20	0.08	0.27	0.22	0.12	0.26	1.25	0.80	1.42	11.11	2.17	19.18
Intracerebral haemorrhage	0.17	0.06	0.26	0.82	0.38	1.53	1.92	0.00	1.98	5.06	1.98	6.14
Cancer (malignant melanoma)	0.24	0.14	0.39	0.18	0.04	0.26	21.12	11.57	28.38	7.77	3.49	16.11
Osteoporosis	0.16	0.10	0.20	0.28	0.07	0.38	2.19	0.74	2.52	2.49	1.45	3.03
Lupus erythematosus (local and systemic)	0.13	0.05	0.18	0.75	0.39	1.04	0.50	0.22	0.65	0.00	0.00	0.00
Unstable angina	0.00	0.00	0.00	1.03	0.45	1.61	0.00	0.00	0.00	0.78	0.31	1.04
Autoimmune liver disease	0.51	0.14	0.56	0.32	0.00	0.61	1.18	0.44	1.16	1.83	0.99	2.91
Cancer (other organs)	0.14	0.00	0.29	0.40	0.09	1.13	8.05	3.99	10.46	5.95	2.02	8.95
Hyperparathyroidism	0.00	0.00	0.00	0.60	0.13	1.70	8.22	1.72	12.63	12.68	2.67	21.44
Macular degeneration	0.16	0.09	0.19	0.18	0.11	0.22	2.01	0.91	2.37	1.76	0.50	3.03
Ankylosing spondylitis	0.30	0.07	0.64	0.20	0.07	0.25	0.00	0.00	0.00	0.00	0.00	0.00
Secondary polycythaemia	0.00	0.00	0.00	0.14	0.00	0.28	0.45	0.00	0.48	0.61	0.32	0.89
Abdominal aortic aneurysm	0.18	0.00	0.36	0.04	0.00	0.09	0.00	0.00	0.00	0.00	0.00	0.00
Aplastic anaemias	0.21	0.07	0.43	0.06	0.00	0.12	0.86	0.49	1.05	2.44	1.34	3.05
Cancer (stomach)	0.00	0.00	0.00	0.60	0.00	1.20	0.00	0.00	0.00	2.02	0.00	2.12
Primary pulmonary hypertension	0.08	0.04	0.16	0.21	0.08	0.26	1.70	0.00	2.00	0.00	0.00	0.00
Secondary pulmonary hypertension	0.08	0.04	0.16	0.13	0.07	0.17	1.70	0.00	2.00	0.00	0.00	0.00
Angiodysplasia of colon	0.00	0.00	0.00	0.04	0.00	0.08	0.34	0.00	0.35	0.00	0.00	0.00
Bronchiectasis	0.15	0.07	0.19	0.08	0.04	0.15	2.14	1.30	2.45	1.49	0.80	1.67
Dementia	0.00	0.00	0.00	0.14	0.00	0.28	0.00	0.00	0.00	0.59	0.26	0.83
Other haemolytic anaemias	0.11	0.04	0.16	0.00	0.00	0.00	0.28	0.00	0.31	0.81	0.28	0.97
Systemic sclerosis	0.25	0.00	0.49	0.07	0.00	0.13	0.00	0.00	0.00	0.00	0.00	0.00
MGUS	0.00	0.00	0.00	0.00	0.00	0.00	0.73	0.20	1.26	1.32	0.79	1.57
Immunodeficiencies	0.12	0.04	0.16	0.00	0.00	0.00	0.81	0.27	0.89	1.54	0.72	2.20
Cancer (hodgkin lymphoma)	0.18	0.00	0.36	0.00	0.00	0.00	84.48	37.08	151.29	63.13	44.96	75.72
Cancer (ovarian)	0.04	0.00	0.07	0.07	0.00	0.15	31.36	1.09	64.02	1.36	0.39	1.72
Cancer (myelodysplastic syndromes)	0.00	0.00	0.00	0.00	0.00	0.00	3.99	0.86	7.04	30.56	0.46	31.68
Posterior uveitis	0.11	0.00	0.22	0.00	0.00	0.00	0.00	0.00	0.00	0.54	0.00	0.57
Cancer (bone and articular cartilage)	0.04	0.00	0.07	0.00	0.00	0.00	1.47	0.84	1.52	19.33	9.98	26.22
Cancer (oro-pharyngeal)	0.00	0.00	0.00	0.09	0.00	0.19	5.84	1.76	5.92	14.68	3.09	13.78
Other interstitial pulmonary diseases with fibrosis	0.08	0.00	0.16	0.00	0.00	0.00	0.00	0.00	0.00	0.29	0.00	0.31
Cancer (lung and trachea)	0.00	0.00	0.00	0.06	0.00	0.12	3.21	0.20	3.63	0.22	0.00	0.23
Cancer (bladder)	0.00	0.00	0.00	0.00	0.00	0.00	0.00	0.00	0.00	0.00	0.00	0.00
Cancer (uterine	0.00	0.00	0.00	0.00	0.00	0.00	0.00	0.00	0.00	0.34	0.00	0.36
Cancer (pancreatic)	0.00	0.00	0.00	0.00	0.00	0.00	0.00	0.00	0.00	0.31	0.00	0.33
Cancer (oesophageal)	0.00	0.00	0.00	0.00	0.00	0.00	0.00	0.00	0.00	3.11	1.47	4.61
Cancer (multiple myeloma and malignant plasma cell neoplasms)	0.00	0.00	0.00	0.00	0.00	0.00	0.00	0.00	0.00	0.00	0.00	0.00
Cancer (multiple independent sites)	0.00	0.00	0.00	0.00	0.00	0.00	0.00	0.00	0.00	1.29	0.00	1.44
Cancer (liver)	0.00	0.00	0.00	0.00	0.00	0.00	0.00	0.00	0.00	0.34	0.00	0.36
Cancer (kidney and ureter)	0.00	0.00	0.00	0.00	0.00	0.00	0.00	0.00	0.00	6.97	1.61	8.79
Cancer (biliary tract)	0.00	0.00	0.00	0.00	0.00	0.00	0.00	0.00	0.00	0.34	0.00	0.36

Table S18. Cumulative burden of 183 condition-specific outcomes in cancer survivors by treatment type at 45 years attained age. Cumulative burden per 100 individuals is shown.

Health conditions	Chemotherapy and radiotherapy			Chemotherapy and surgery			Chemotherapy only			Radiotherapy and surgery			Radiotherapy only			Surgery only		
	Cumulative burden	Lower CI	Upper CI	Cumulative burden	Lower CI	Upper CI	Cumulative burden	Lower CI	Upper CI	Cumulative burden	Lower CI	Upper CI	Cumulative burden	Lower CI	Upper CI	Cumulative burden	Lower CI	Upper CI
Mental health	290.93	161.59	420.44	434.20	305.91	502.68	248.50	199.26	330.41	241.93	188.29	306.88	354.26	259.13	722.75	327.12	275.89	378.54
Bacterial infections	190.11	110.19	242.63	110.50	113.34	169.35	76.97	73.66	80.40	103.98	68.35	169.08	72.71	45.67	80.33	62.40	51.95	69.33
Hypertension	188.71	117.27	322.90	112.13	115.84	198.90	46.57	33.29	66.00	37.29	10.83	47.29	94.86	48.30	140.58	31.26	16.75	38.18
Migraine	11.02	4.16	11.67	28.89	13.19	34.91	19.25	11.24	24.31	8.92	6.29	12.92	24.34	8.49	26.18	26.58	25.05	32.98
Hypo or hyperthyroidism	335.88	199.01	388.03	49.33	17.78	61.38	57.00	34.48	79.28	186.27	141.57	249.86	189.97	89.23	213.14	97.30	80.60	112.56
Menorrhagia and polymenorrhoea	37.68	5.72	75.80	28.35	13.92	33.77	7.51	6.24	10.26	2.38	0.77	4.98	0.00	0.00	0.00	18.87	14.51	21.74
Asthma	23.18	14.61	28.13	23.66	19.70	26.02	18.95	15.54	24.31	30.03	20.51	37.56	25.39	17.00	32.06	30.13	28.20	31.31
Oesophagitis and oesophageal ulcer	42.48	18.24	68.53	15.71	7.47	19.42	17.84	14.96	23.57	27.63	15.13	37.35	23.06	13.45	25.07	22.94	23.30	32.42
Diabetic ophthalmic complications	109.68	49.08	195.74	21.28	7.71	27.16	64.78	12.44	118.02	46.79	21.03	48.28	102.47	91.06	208.21	27.40	18.07	31.03
Obesity	23.52	7.83	31.72	10.73	8.05	13.15	11.26	9.31	13.91	21.42	14.02	19.67	12.29	7.50	15.25	16.95	15.65	18.56
Infections of other or unspecified organs	229.69	93.17	369.12	57.07	40.37	75.45	37.11	34.61	43.08	51.86	34.33	75.35	40.98	21.54	53.97	30.16	24.43	33.49
Enthesopathies and synovial disorders	22.17	16.84	36.10	9.72	5.64	12.27	6.45	4.97	7.75	3.59	2.42	6.08	18.67	8.86	28.27	12.31	9.41	12.91
Urolithiasis	77.33	40.24	146.09	12.15	11.02	22.43	10.24	7.59	13.93	11.09	2.56	20.04	0.00	0.00	0.00	8.69	6.17	13.11
Diabetes	18.00	10.82	25.82	10.06	9.50	13.11	14.07	12.32	18.43	16.39	9.98	16.07	12.93	8.93	20.71	14.81	13.56	15.95
Diabetic neurological complications	47.40	45.89	90.60	21.28	7.71	27.16	62.11	11.47	116.00	48.86	18.96	49.12	90.64	85.23	199.69	24.44	13.30	26.65
Infection of skin and subcutaneous tissues	42.59	37.21	54.08	21.44	21.83	30.16	27.93	25.01	32.36	25.78	14.62	33.02	28.32	15.38	31.23	13.70	8.56	14.14
Ear and upper respiratory tract infections	106.25	20.79	202.73	18.28	16.05	29.66	8.94	4.85	12.20	8.97	5.90	14.42	16.61	8.00	22.19	8.66	7.51	8.49
Cholelithiasis	9.86	1.40	14.46	2.19	1.36	4.31	6.23	3.74	11.38	3.52	1.00	5.73	12.06	2.70	33.11	13.28	10.56	21.19
HIV	10.21	6.01	13.26	16.26	5.31	21.91	7.56	4.73	9.58	1.39	1.26	3.06	0.00	0.00	0.00	7.05	5.22	8.67
Lower respiratory tract infections	232.02	93.15	384.33	47.59	29.79	71.11	51.36	40.49	70.85	36.72	28.19	44.16	48.09	32.58	80.14	16.53	12.18	22.12
Gastrooesophageal reflux disease	6.80	5.24	9.14	5.16	4.14	5.73	12.79	10.32	16.64	11.70	7.75	20.77	13.73	9.30	15.49	9.45	9.85	11.75
Coronary heart disease not otherwise specified	71.01	33.57	99.21	16.50	4.74	20.77	0.96	0.61	1.81	0.00	0.00	0.00	5.42	0.00	12.20	8.26	7.04	11.19
Dysmenorrhoea	9.44	2.90	17.25	9.10	3.27	11.37	5.33	3.38	10.89	18.13	7.48	32.18	0.00	0.00	0.00	8.24	7.25	10.14
Urinary incontinence	63.05	19.13	91.30	15.38	10.20	21.84	9.86	7.49	12.45	17.61	10.38	24.66	5.61	0.00	8.66	18.92	17.17	25.30
Infections of the digestive system	53.50	34.74	68.16	24.20	22.44	38.51	39.93	19.48	56.89	22.67	12.44	27.77	6.74	3.56	8.51	12.46	9.04	14.90
Urinary tract infections	85.53	40.86	120.10	23.59	19.06	40.51	22.09	17.90	26.42	20.83	6.14	30.00	1.12	0.00	2.16	17.60	14.76	21.11
Peritonitis	6.50	2.14	10.17	15.87	15.81	24.28	22.16	15.00	24.06	18.02	10.63	25.85	6.67	1.29	8.84	12.26	10.15	14.23
Cholecystitis	8.72	1.40	12.33	3.20	3.14	5.94	5.37	2.82	12.35	3.52	1.00	5.73	12.06	1.39	30.63	9.25	8.18	12.38
Endometriosis	2.58	0.00	5.49	0.88	0.00	0.88	2.43	0.99	5.74	10.68	4.17	17.19	1.25	0.00	2.56	5.29	3.33	6.58
Ulcerative colitis	0.00	0.00	0.00	16.72	0.00	34.11	6.37	0.40	12.81	8.08	0.00	16.76	0.00	0.00	0.00	7.72	1.90	12.61
Cholangitis	8.69	1.40	12.29	5.68	3.14	12.53	5.27	2.74	12.04	3.52	1.00	5.73	4.59	1.39	10.82	9.95	8.29	13.42
Cancer (breast)	33.92	13.28	73.11	42.66	4.87	70.78	13.51	12.48	27.32	185.84	30.74	291.84	15.10	2.77	33.28	11.45	0.98	21.88
Chronic sinusitis	11.00	7.46	12.67	5.63	4.16	6.54	5.93	6.39	7.64	2.96	1.62	3.29	4.00	2.56	7.16	6.96	5.70	7.60
Polycystic ovarian syndrome	3.14	0.00	3.34	0.00	0.00	0.00	6.04	3.77	9.33	5.98	3.35	5.72	8.87	7.80	17.79	3.36	2.83	4.52
Gastritis and duodenitis	12.06	7.82	16.79	1.85	0.00	3.23	7.25	5.13	10.63	4.36	2.52	5.44	5.34	2.53	9.79	8.54	7.89	9.63
Anorectal fistula	4.71	2.85	9.53	21.06	13.66	36.63	3.93	2.70	4.71	0.00	0.00	0.00	5.37	2.73	8.50	3.76	2.23	5.09
Postviral fatigue syndrome, neurasthenia and fibromyalgia	13.87	12.04	16.03	9.84	8.18	14.85	5.14	3.09	7.19	11.87	7.22	14.87	11.38	5.76	15.57	7.78	6.84	9.01
Hepatic failure	6.06	1.90	13.42	14.30	7.34	23.41	18.56	7.84	26.25	3.02	1.99	4.12	7.86	1.34	14.04	6.92	4.36	8.40
Viral diseases (excluding chronic hepatitis/HIV)	98.07	55.35	146.87	27.89	13.31	46.73	21.42	17.40	25.90	8.23	3.92	11.22	24.85	13.04	41.30	7.12	5.69	8.16
Chronic viral hepatitis	6.93	2.01	10.35	8.73	5.56	11.43	5.36	3.78	6.55	6.09	2.95	7.65	0.00	0.00	0.00	5.80	4.93	6.82
Pancreatitis	3.03	0.00	5.17	21.10	7.08	39.98	5.51	3.08	8.89	1.70	0.00	3.31	0.00	0.00	0.00	0.74	0.26	0.66
Other anaemias	13.32	8.03	20.69	11.23	8.65	12.70	9.12	6.19	11.58	9.77	7.20	11.76	5.91	4.07	9.65	6.87	5.36	8.22
Venous thromboembolic disease	8.33	2.89	11.34	8.30	2.87	10.99	5.26	3.38	9.96	12.65	7.48	13.15	20.10	12.77	27.17	6.49	6.36	11.57
Osteoarthritis (excluding spine)	9.91	3.96	15.23	4.81	0.00	7.19	3.50	2.30	4.07	2.94	1.92	4.58	4.26	1.14	6.40	2.60	2.20	3.26
Cancer metastasis (other organs)	198.96	83.39	426.20	112.83	74.30	235.11	75.34	70.17	134.72	379.53	166.91	622.01	68.44	25.00	95.09	51.56	18.74	50.42
COPD	8.76	3.82	10.36	5.79	1.91	9.12	5.18	1.77	7.22	3.26	1.84	5.98	1.36	0.00	2.74	3.74	2.57	4.18
Intracranial hypertension	50.83	2.09	100.55	1.29	0.00	1.82	19.17	1.63	39.81	6.09	2.50	8.46	11.10	5.25	13.43	2.88	1.80	3.87
Septicaemia	47.84	32.79	57.56	34.56	34.13	55.15	13.74	9.30	16.85	19.21	10.13	25.80	24.18	12.71	32.85	9.99	5.28	14.95
Diverticular disease of intestine	1.46	0.00	3.44	0.59	0.00	1.78	7.26	0.40	7.86	6.08	0.00	9.01	0.00	0.00	0.00	1.73	1.26	2.45
Coeliac disease	2.90	0.67	2.36	3.88	2.67	7.48	1.35	0.44	1.54	0.00	0.00	0.00	0.00	0.00	0.00	2.96	1.96	4.09
Erectile dysfunction	3.24	1.07	5.17	10.49	7.77	13.19	5.18	3.31	10.97	0.76	0.00	0.79	4.43	2.79	6.97	4.07	2.61	5.38
Epilepsy	7.89	4.01	12.41	5.89	4.17	7.17	4.66	2.53	8.12	18.77	12.59	21.63	23.94	16.45	28.92	13.46	11.36	15.29
Fatty liver	3.99	1.90	7.88	5.33	2.97	8.65	6.54	4.47	9.13	2.79	0.77	3.51	8.07	2.83	12.76	2.80	2.11	4.10
Nonrheumatic mitral valve disorders	2.28	0.00	3.69	12.65	5.34	19.97	1.59	0.62	2.52	0.00	0.00	0.00	7.46	1.29	13.77	0.71	0.09	1.60
Heart failure	58.88	21.23	85.01	14.84	12.38	26.29	13.96	9.40	20.85	7.47	0.00	7.55	13.68	2.41	27.37	2.29	1.09	2.76
Hypertrophy of nasal turbinates	2.97	1.13	4.35	3.73	2.23	6.39	0.00	0.00	0.56	1.72	0.00	3.70	5.76	2.85	8.03	2.56	1.90	3.75
Peripheral neuropathies	13.53	8.43	21.17	3.05	2.68	5.43	2.65	1.79	4.38	6.47	0.98	11.09	12.74	5.13	15.95	4.44	3.64	5.33
Cancer metastasis (retroperitoneum and peritoneum	180.15	63.40	409.50	104.23	60.10	227.58	68.47	61.42	126.13	160.97	97.87	309.93	33.33	13.51	55.78	41.22	10.71	40.34
Cancer metastasis (pleura)	180.15	63.40	409.50	104.23	60.10	227.58	68.47	61.42	126.13	160.97	97.87	309.93	33.33	13.51	55.78	41.22	10.71	40.34
Cancer metastasis (lung)	181.18	64.41	413.68	104.23	60.10	227.58	69.99	61.42	127.63	160.97	97.87	309.93	33.33	13.51	55.78	42.78	10.90	42.05
Cancer metastasis (liver and intrahepatic bile duct)	180.15	63.40	409.50	104.23	60.10	227.58	68.93	61.91	127.38	163.05	97.87	311.81	33.33	13.51	55.78	41.49	11.03	40.51
Cancer metastasis (bowel)	180.15	63.40	409.50	104.23	60.10	227.58	68.47	61.42	126.13	160.97	97.87	309.93	33.33	13.51	55.78	41.22	10.71	40.34
Meningitis	40.92	36.80	54.24	16.40	7.79	25.99	4.77	3.15	7.39	11.11	5.27	19.00	24.12	12.08	29.95	4.13	3.09	6.07
Other cardiomyopathy	163.45	46.91	261.25	10.12	1.32	16.95	155.47	31.57	353.16	0.00	0.00	0.00	7.89	2.66	14.69	4.34	4.13	7.30
Infections of the heart	17.67	10.91	24.03	7.40	4.91	10.92	5.35	3.31	6.33	8.89	2.60	17.08	15.82	7.45	19.62	2.63	1.93	4.89
Peripheral arterial disease	0.00	0.00	0.00	0.54	0.00	1.63	1.10	0.00	2.62	6.34	0.99	10.99	1.25	0.00	2.56	1.46	0.73	1.89
Female infertility	0.00	0.00	0.00	2.64	0.52	4.48	2.01	0.65	3.31	0.91	0.00	1.95	7.41	3.33	12.91	1.64	1.20	2.29
Cancer metastasis (lymph nodes)	15.73	13.05	27.82	44.88	19.97	84.33	40.85	25.65	64.80	110.63	64.48	216.71	10.10	6.47	20.56	12.99	7.53	21.38
Cancer (leukaemia)	904.73	595.32	1234.27	302.30	140.85	512.10	200.94	164.35	276.72	59.43	34.43	83.08	151.01	118.53	220.39	28.93	1.64	53.29
Pneumothorax	2.44	0.00	4.99	0.76	0.00	1.43	2.79	1.97	4.92	0.99	0.00	1.91	1.50	0.00	2.98	1.74	1.00	2.31
Acute kidney injury	21.85	14.47	30.70	6.14	4.16	13.67	10.92	3.72	16.27	6.75	4.05	9.31	6.39	4.13	12.26	3.19	1.94	4.21
Cancer (brain, other cns and intracranial)	57.95	11.78	94.63	8.29	1.51	15.39	8.99	6.55	13.98	298.58	250.47	445.51	134.23	78.22	254.23	61.44	56.95	95.91
Pulmonary embolism	9.01	4.38	16.07	0.88	0.00	0.88	1.24	0.32	2.64	7.69	3.26	10.62	4.99	1.56	13.93	3.35	1.14	4.28
Mycoses	87.37	11.81	170.45	8.46	5.86	10.39	6.38	4.41	9.59	8.89	2.60	17.08	14.39	4.51	14.90	2.93	1.64	5.01
Portal hypertension	3.99	1.90	7.88	4.76	2.38	8.13	4.19	2.82	6.20	2.03	0.00	3.19	5.01	0.00	9.65	2.01	1.33	2.72
Raynaud's syndrome	6.64	1.67	15.01	0.54	0.00	1.63	0.42	0.00	1.16	0.00	0.00	0.00	0.00	0.00	3.38	1.02	0.66	1.39
Cancer (other skin and subcutaneous tissue)	3.95	0.71	5.04	0.59	0.00	2.35	1.26	0.61	2.49	0.00	0.00	0.00	2.86	0.00	3.00	45.54	18.03	73.40
Sleep apnoea	6.85	4.57	15.32	1.60	0.00	3.75	1.17	0.33	1.94	6.46	3.25	7.73	2.54	1.10	5.61	1.96	1.49	2.57
Male infertility	0.95	0.00	1.65	5.94	1.33	10.95	4.13	2.51	6.08	0.91	0.00	1.95	10.10	6.21	20.58	0.97	0.32	1.34
Cancer (testicular)	2.03	1.00	3.93	35.03	27.61	61.78	11.88	4.33	11.68	33.47	1.26	103.66	2.43	0.00	4.83	28.68	18.47	40.95
Eye infections	26.40	22.20	36.79	9.68	5.30	13.07	1.89	1.16	2.67	1.05	0.00	2.06	9.38	3.45	11.46	1.65	0.81	1.95
Bell's palsy	5.10	0.00	5.95	4.92	0.72	8.50	0.00	0.00	0.93	3.96	3.14	9.67	9.90	1.45	10.46	1.95	0.72	2.93
Cancer (colorectal and anus)	0.00	0.00	0.00	16.78	0.00	35.83	18.78	0.00	54.20	7.12	0.00	13.86	0.00	0.00	0.00	5.89	2.59	9.30
End stage renal disease	50.97	45.56	60.45	19.76	13.26	27.65	17.92	17.39	22.46	16.31	10.42	20.76	26.12	18.51	29.71	8.32	6.61	9.24
Hypertrophic cardiomyopathy	88.04	1.54	125.79	0.00	0.00	0.00	154.60	30.74	352.48	0.00	0.00	0.00	2.84	1.58	4.10	2.50	0.44	4.89
Pleural effusion	9.20	5.45	13.00	8.42	6.74	15.85	23.26	8.51	42.33	13.48	9.71	19.95	9.63	7.43	14.02	4.37	3.59	6.22
Myocardial infarction	71.01	33.57	99.21	8.34	1.32	13.57	0.34	0.00	0.69	0.00	0.00	0.00	5.42	0.00	12.20	3.87	1.20	8.23
Dilated cardiomyopathy	88.13	1.54	125.83	0.00	0.00	0.00	156.18	34.12	355.31	0.00	0.00	0.00	2.84	1.58	4.10	0.55	0.39	1.10
Alcoholic liver disease	0.00	0.00	0.00	3.04	0.00	6.52	3.04	1.65	3.98	0.00	0.00	0.00	1.49	0.00	2.69	0.93	0.50	1.79
Right bundle branch block	1.30	0.00	2.67	0.00	0.00	0.00	0.79	0.00	1.55	31.39	0.00	67.21	2.81	2.65	6.35	1.06	0.48	1.43
Bifascicular block	1.30	0.00	2.67	0.00	0.00	0.00	0.79	0.00	1.55	31.39	0.00	67.21	2.81	2.65	6.35	1.06	0.48	1.43
Supraventricular tachycardia	3.78	0.00	7.16	2.41	2.24	4.36	2.50	0.31	5.97	2.78	0.00	5.41	0.00	0.00	0.00	2.26	0.94	4.26
Oesophageal varices	0.00	0.00	0.00	0.00	0.00	0.00	12.52	1.89	16.71	0.00	0.00	0.00	0.00	0.00	0.00	0.00	0.00	0.00
Ventricular tachycardia	3.78	0.00	7.16	2.41	2.24	4.36	2.50	0.31	5.97	2.78	0.00	5.41	0.00	0.00	0.00	2.26	0.94	4.26
Glomerulonephritis	0.00	0.00	9.80	3.47	0.00	7.02	43.72	0.00	92.37	0.81	0.00	1.57	5.72	0.00	11.74	0.25	0.00	0.50
Barrett's oesophagus	11.33	0.00	13.96	1.96	1.15	3.35	3.82	1.68	6.46	0.00	0.00	0.00	1.36	0.00	2.77	0.86	0.42	1.32
Obstructive and reflux uropathy	22.72	10.19	40.58	10.45	7.44	22.44	6.77	2.68	9.01	8.00	5.18	13.88	0.00	0.00	0.00	3.60	1.95	7.29
Infection of anal and rectal regions	1.06	0.00	2.21	8.31	6.25	17.33	3.88	0.77	8.16	1.79	0.00	3.51	0.00	0.00	0.00	0.29	0.13	0.43
Disorders of autonomic nervous system	5.00	2.12	7.36	0.00	0.00	0.00	0.96	0.32	1.88	5.01	3.56	10.75	2.50	1.16	3.31	2.75	2.72	3.64
Nasal polyp	1.30	0.00	2.25	1.80	0.00	3.17	0.83	0.00	2.05	0.00	0.00	0.00	2.80	1.40	6.12	0.76	0.32	1.14
Trifascicular block	1.30	0.00	2.67	0.00	0.00	0.00	0.00	0.00	0.00	29.21	0.00	67.21	2.81	2.65	6.35	1.06	0.48	1.43
Nonrheumatic aortic valve disorders	0.00	0.00	0.00	0.00	0.00	0.00	14.68	10.29	18.82	0.00	0.00	0.00	1.36	0.00	1.46	0.00	0.00	0.00
Anterior and intermediate uveitis	6.61	2.26	9.87	0.00	0.00	0.00	0.00	0.00	0.00	0.00	0.00	0.00	0.00	0.00	0.00	0.22	0.00	0.63
Pulmonary collapse (excluding pneumothorax)	22.43	15.49	24.27	3.79	1.38	7.88	4.54	2.48	7.71	4.58	2.27	7.76	2.90	1.34	7.62	2.30	1.71	3.33
Stable angina	0.00	0.00	9.80	0.76	0.00	2.33	0.00	0.00	0.00	0.00	0.00	0.00	0.00	0.00	0.00	1.49	0.92	2.06
Stroke(not otherwise specified)	0.00	0.00	0.00	0.00	0.00	0.00	13.01	0.81	23.50	2.51	0.00	2.57	19.05	13.00	41.31	5.89	2.31	14.68
Atrial fibrillation	0.00	0.00	0.00	1.21	0.00	2.49	1.08	0.31	1.92	3.20	0.00	3.39	2.52	0.00	3.42	0.68	0.14	0.82
Endometrial hyperplasia and hypertrophy	1.14	0.00	1.22	1.42	0.52	3.46	0.00	0.00	0.00	5.58	2.05	6.20	1.15	0.00	2.42	1.36	0.78	1.99
Cancer (non-hodgkin lymphoma)	96.07	38.72	94.91	136.38	36.08	257.93	18.98	17.21	30.69	49.37	15.10	103.87	18.28	0.00	23.53	24.53	6.76	45.21
Multiple valve disease	4.97	0.00	8.07	0.00	0.00	0.00	2.43	0.64	3.68	0.00	0.00	0.00	3.97	1.37	6.64	0.00	0.00	0.00
Cancer metastasis (brain, cns and intracranial)	155.45	35.82	330.21	9.13	4.75	12.69	15.18	11.89	24.77	278.19	122.20	451.88	48.18	16.40	80.22	15.44	9.04	18.32
Cancer metastasis (bone)	159.15	35.82	338.52	8.59	4.75	12.13	14.85	11.89	24.11	280.43	122.20	453.81	47.06	16.40	79.15	15.05	9.04	17.75
Cancer metastasis (adrenal gland)	155.45	35.82	330.21	8.59	4.75	12.13	14.85	11.89	24.11	278.19	122.20	451.88	47.06	16.40	79.15	15.05	9.04	17.75
Crohn's disease	1.66	0.00	3.77	0.73	0.00	0.74	0.72	0.37	1.50	0.81	0.00	1.62	0.00	0.00	0.00	0.72	0.38	1.24
Cataract	28.76	26.41	51.81	0.75	0.00	1.43	8.27	5.22	10.61	1.07	0.00	2.09	15.89	5.92	23.42	0.14	0.14	0.28
Cancer (cervical)	7.38	0.91	16.61	0.00	0.00	0.00	0.00	0.00	0.00	2.96	1.00	4.00	0.00	0.00	0.00	0.56	0.00	1.27
Thrombophilia	0.00	0.00	0.00	1.99	0.62	2.43	0.52	0.00	1.65	1.07	0.00	1.09	2.19	0.00	4.69	1.65	1.03	2.56
Encephalitis	29.26	22.20	38.92	9.07	2.64	16.86	1.04	0.32	1.99	1.05	0.00	2.06	8.27	3.45	10.16	1.46	0.54	1.83
Agranulocytosis	33.50	26.87	47.59	22.56	10.94	38.15	7.99	6.31	11.00	13.45	7.64	17.34	26.54	9.13	26.71	6.19	2.28	8.62
Subarachnoid haemorrhage	0.00	0.00	0.00	0.00	0.00	0.00	1.52	0.81	2.56	7.94	0.00	10.88	4.03	3.89	8.14	4.92	1.49	12.06
Transient ischaemic attack	5.64	0.00	11.93	0.00	0.00	0.00	1.82	0.60	3.39	0.91	0.00	0.96	5.26	1.65	11.40	0.78	0.54	1.67
Rheumatoid arthritis	6.13	3.62	7.72	0.00	0.00	0.00	0.42	0.00	0.88	1.62	0.00	2.37	0.00	0.00	0.00	2.69	1.45	4.00
Liver fibrosis sclerosis and cirrhosis	0.00	0.00	0.00	3.04	0.00	6.52	3.10	0.91	5.70	0.76	0.00	1.52	0.00	0.00	0.00	0.71	0.25	1.05
Ischaemic stroke	0.00	0.00	0.00	0.00	0.00	0.00	2.86	1.15	4.38	6.81	1.05	11.65	42.96	15.14	69.82	5.79	1.93	13.55
Left bundle branch block	0.85	0.00	1.68	0.00	0.00	0.00	2.29	0.00	4.67	0.00	0.00	0.00	0.00	0.00	0.00	0.91	0.46	1.78
Scoliosis	5.12	1.56	9.36	0.00	0.00	0.00	1.51	1.10	2.62	0.95	0.00	2.02	1.42	0.00	1.67	3.20	1.81	4.26
Infection of liver	24.13	9.93	38.29	9.26	6.08	18.03	3.61	1.08	6.26	1.57	0.00	3.14	2.65	1.33	5.82	1.54	0.86	2.08
Atrioventricular block, second degree	0.85	0.00	1.68	0.00	0.00	0.00	0.76	0.00	1.56	0.00	0.00	0.00	0.00	0.00	0.00	0.91	0.46	1.78
Atrioventricular block, first degree	0.85	0.00	1.68	0.00	0.00	0.00	0.76	0.00	1.56	0.00	0.00	0.00	0.00	0.00	0.00	0.91	0.46	1.78
Atrioventricular block, complete	0.85	0.00	1.68	0.00	0.00	0.00	0.76	0.00	1.56	0.00	0.00	0.00	0.00	0.00	0.00	0.91	0.46	1.78
Infection of bones and joints	3.69	1.85	6.44	1.12	0.00	2.18	2.39	0.00	6.55	24.44	2.27	64.17	0.00	0.00	0.00	2.07	0.21	2.34
Cancer (thyroid)	0.00	0.00	0.00	2.44	0.00	2.50	1.44	1.38	2.92	129.00	22.86	198.38	3.88	0.00	7.40	9.10	5.23	12.48
Glaucoma	1.06	1.01	2.21	0.00	0.00	0.00	0.00	0.00	0.00	0.00	0.00	0.00	0.00	0.00	0.00	0.00	0.00	0.00
Secondary or other thrombocytopaenia	10.01	4.84	18.27	6.85	3.02	11.34	4.27	2.94	5.04	2.67	1.01	4.82	6.44	5.77	10.17	2.04	0.66	3.07
Enteropathic arthropathy	0.00	0.00	0.00	0.59	0.00	1.22	1.75	0.00	3.39	0.00	0.00	0.00	1.50	0.00	2.98	0.33	0.00	0.92
Non-acute cystitis	3.50	0.00	4.85	0.73	0.00	1.42	1.04	0.36	1.03	0.00	0.00	0.00	1.12	0.00	2.16	0.62	0.26	0.97
Sick sinus syndrome	1.71	0.00	3.58	1.21	0.00	2.49	8.17	0.00	15.97	0.00	0.00	0.00	0.00	0.00	0.00	1.26	0.55	1.68
Essential tremor	20.27	7.38	28.38	0.00	0.00	0.00	0.00	0.00	0.00	1.19	0.00	3.57	0.00	0.00	0.00	0.00	0.00	0.00
Primary or idiopathic thrombocytopaenia	8.43	4.30	15.10	6.85	3.02	11.34	4.04	2.87	5.04	2.67	1.01	4.82	3.09	1.45	8.30	1.48	0.51	2.04
Respiratory failure	15.56	9.38	20.04	2.41	1.16	2.73	5.64	3.25	10.70	3.53	1.60	6.64	4.10	0.00	8.14	2.30	1.52	3.04
Spinal stenosis	75.07	4.12	159.00	1.24	0.00	2.49	1.30	0.00	2.55	0.00	0.00	0.00	0.00	0.00	0.00	0.12	0.00	0.24
Multiple sclerosis	8.69	0.00	14.12	0.00	0.00	0.00	0.00	0.00	0.00	0.91	0.00	0.96	2.25	0.00	6.43	0.41	0.00	0.88
Pericardial effusion (noninflammatory)	4.14	0.00	9.04	6.95	2.41	13.19	14.12	4.03	25.48	1.46	0.00	1.70	0.00	0.00	0.00	0.66	0.00	1.33
Intracerebral haemorrhage	0.00	0.00	0.00	0.00	0.00	0.00	1.99	0.81	3.01	5.24	2.58	8.78	5.64	5.48	11.36	4.69	1.13	11.48
Cancer (malignant melanoma)	2.85	0.00	5.26	0.00	0.00	0.00	2.20	0.00	4.57	41.81	14.74	81.93	1.25	0.00	1.28	21.31	15.39	28.45
Osteoporosis	7.23	4.04	8.78	1.53	1.07	2.51	2.74	2.26	3.38	2.17	0.00	4.44	2.84	0.00	5.34	1.06	0.53	1.50
Lupus erythematosus (local and systemic)	0.00	0.00	0.00	0.00	0.00	0.00	0.40	0.00	0.42	0.00	0.00	0.00	0.00	0.00	0.00	0.60	0.25	0.58
Unstable angina	0.00	0.00	0.00	0.76	0.00	2.33	0.00	0.00	0.00	0.00	0.00	0.00	1.50	0.00	3.06	1.31	0.74	1.88
Autoimmune liver disease	0.00	0.00	0.00	0.87	0.00	2.60	3.42	1.58	5.66	0.76	0.00	1.52	0.00	0.00	0.00	0.24	0.10	0.44
Cancer (other organs)	110.76	30.14	224.55	13.66	1.45	20.71	66.84	15.24	179.79	15.39	3.49	34.16	14.32	8.59	19.15	7.41	4.31	9.68
Hyperparathyroidism	5.47	2.36	6.79	1.39	0.00	2.69	75.38	12.00	144.75	51.56	0.00	174.46	0.00	0.00	0.00	35.48	2.91	69.02
Macular degeneration	2.07	0.00	4.52	0.00	0.00	0.00	0.79	0.33	0.97	1.05	0.00	2.06	1.90	0.00	4.39	1.26	0.40	1.63
Ankylosing spondylitis	1.37	0.00	3.11	0.00	0.00	0.00	0.00	0.00	0.00	0.00	0.00	0.00	0.00	0.00	0.00	0.00	0.00	0.00
Secondary polycythaemia	1.99	0.00	2.87	1.93	0.00	2.05	0.64	0.00	1.62	0.00	0.00	0.00	0.00	0.00	0.00	0.21	0.09	0.36
Abdominal aortic aneurysm	1.14	0.00	3.11	0.00	0.00	0.00	4.21	0.00	8.83	0.00	0.00	0.00	0.00	0.00	0.00	0.14	0.00	0.30
Aplastic anaemias	1.37	0.00	2.76	0.00	0.00	0.00	1.97	0.97	2.40	2.15	0.78	2.82	0.00	0.00	0.00	1.11	0.27	1.50
Cancer (stomach)	6.62	0.00	13.23	0.59	0.00	1.13	0.00	0.00	0.00	0.00	0.00	0.00	0.00	0.00	0.00	0.00	0.00	0.00
Primary pulmonary hypertension	0.00	0.00	0.00	0.00	0.00	0.00	0.00	0.00	0.00	0.00	0.00	0.00	0.00	0.00	0.00	0.81	0.18	1.40
Secondary pulmonary hypertension	0.00	0.00	0.00	0.00	0.00	0.00	0.00	0.00	0.00	0.00	0.00	0.00	0.00	0.00	0.00	0.81	0.18	1.40
Angiodysplasia of colon	0.00	0.00	0.00	0.75	0.00	2.13	0.00	0.00	1.11	0.00	0.00	0.00	0.00	0.00	0.00	0.00	0.00	0.00
Bronchiectasis	6.58	2.98	10.44	0.72	0.00	1.99	0.72	0.37	1.40	0.00	0.00	0.00	3.78	2.33	7.11	0.97	0.69	1.65
Dementia	0.00	0.00	0.00	0.87	0.00	2.60	0.63	0.30	0.92	0.85	0.00	0.89	0.00	0.00	0.00	0.00	0.00	0.00
Other haemolytic anaemias	0.00	0.00	0.00	0.76	0.00	2.14	1.25	0.62	1.52	0.00	0.00	0.00	0.00	0.00	0.00	0.16	0.00	0.16
Systemic sclerosis	2.07	0.00	2.44	0.00	0.00	0.00	0.00	0.00	0.00	0.00	0.00	0.00	0.00	0.00	0.00	0.43	0.36	0.79
MGUS	0.00	0.00	0.00	0.00	0.00	0.00	0.69	0.00	0.98	0.00	0.00	0.00	0.00	0.00	0.00	0.56	0.26	0.89
Immunodeficiencies	0.00	0.00	0.00	2.32	1.33	5.08	0.68	0.00	1.57	0.00	0.00	0.00	0.00	0.00	0.00	0.41	0.00	0.85
Cancer (hodgkin lymphoma)	324.19	62.21	449.92	76.46	68.04	88.80	141.29	75.00	142.59	62.00	20.83	93.54	73.82	21.10	129.90	85.67	21.33	139.79
Cancer (ovarian)	1.37	0.00	4.18	0.85	0.78	1.67	0.00	0.00	0.00	0.00	0.00	0.00	0.00	0.00	0.00	15.45	1.36	42.08
Cancer (myelodysplastic syndromes)	100.99	1.02	204.39	0.00	0.00	0.00	42.34	18.92	63.13	1.62	0.00	3.25	49.69	11.07	74.85	0.48	0.25	1.18
Posterior uveitis	2.07	0.00	4.52	0.00	0.00	0.00	0.00	0.00	0.00	0.00	0.00	0.00	0.00	0.00	0.00	0.28	0.00	0.55
Cancer (bone and articular cartilage)	127.22	38.93	129.36	8.37	6.57	11.49	62.01	41.61	80.35	32.53	12.27	69.21	12.35	0.00	28.43	75.13	0.19	75.48
Cancer (oro-pharyngeal)	63.48	2.82	89.86	0.00	0.00	0.00	0.00	0.00	0.00	3.97	0.00	7.72	0.00	0.00	0.00	0.41	0.00	0.80
Other interstitial pulmonary diseases with fibrosis	8.84	0.00	13.12	0.00	0.00	0.00	7.01	0.81	13.40	0.00	0.00	0.00	0.00	0.00	0.00	1.77	0.62	4.14
Cancer (lung and trachea)	0.85	0.00	1.79	6.21	0.00	11.99	7.54	1.02	18.12	0.00	0.00	0.00	0.00	0.00	0.00	0.34	0.00	0.36
Cancer (bladder)	0.00	0.00	0.00	0.00	0.00	0.00	0.00	0.00	4.44	0.00	0.00	0.00	0.00	0.00	0.00	0.62	0.23	0.98
Cancer (uterine	0.00	0.00	0.00	0.00	0.00	0.00	0.00	0.00	0.00	0.00	0.00	0.00	0.00	0.00	0.00	0.13	0.00	0.25
Cancer (pancreatic)	0.00	0.00	0.00	60.68	0.00	171.87	0.00	0.00	0.00	0.00	0.00	0.00	0.00	0.00	0.00	0.11	0.11	0.21
Cancer (oesophageal)	13.66	0.00	18.81	0.00	0.00	0.00	0.00	0.00	0.00	0.00	0.00	0.00	0.00	0.00	0.00	0.00	0.00	0.00
Cancer (multiple myeloma and malignant plasma cell neoplasms)	1.30	0.00	2.67	0.00	0.00	0.00	0.83	0.00	0.88	0.00	0.00	0.00	0.00	0.00	0.00	0.00	0.00	0.00
Cancer (multiple independent sites)	0.00	0.00	0.00	0.00	0.00	0.00	0.00	0.00	0.00	0.00	0.00	0.00	0.00	0.00	0.00	1.59	0.20	2.53
Cancer (liver)	0.00	0.00	0.00	0.76	0.00	1.43	0.00	0.00	0.00	2.17	0.00	5.00	0.00	0.00	0.00	0.00	0.00	0.00
Cancer (kidney and ureter)	0.00	0.00	0.00	5.55	3.68	14.07	0.00	0.00	0.56	0.00	0.00	0.00	0.00	0.00	0.00	2.69	0.83	3.48
Cancer (biliary tract)	0.00	0.00	0.00	0.76	0.00	1.43	0.00	0.00	0.00	2.17	0.00	5.00	0.00	0.00	0.00	0.00	0.00	0.00

Table S19. Cumulative burden of 183 condition-specific outcomes in cancer survivors by chemotherapy type at 45 years attained age. Cumulative burden per 100 individuals is shown.

Health conditions	Alkylating agents			Anthracyclines			Antimetabolites			Chemotherapy unspecified			Hormonal agents (including corticosteroid hormones and sex hormones)			Non-anthracycline antitumour antibiotics			Plant alkaloids and natural products (excluding vinca alkaloids)			Platinum agents			Vinca alkaloids		
	Cumulative burden	Lower CI	Upper CI	Cumulative burden	Lower CI	Upper CI	Cumulative burden	Lower CI	Upper CI	Cumulative burden	Lower CI	Upper CI	Cumulative burden	Lower CI	Upper CI	Cumulative burden	Lower CI	Upper CI	Cumulative burden	Lower CI	Upper CI	Cumulative burden	Lower CI	Upper CI	Cumulative burden	Lower CI	Upper CI
Mental health	542.03	326.20	896.01	503.94	341.75	725.39	573.97	304.01	751.61	268.33	210.24	298.77	298.47	141.21	475.92	389.09	279.76	656.07	619.96	284.97	818.03	729.16	517.30	1368.17	335.22	207.79	482.91
Bacterial infections	93.66	67.99	111.82	136.42	110.43	160.06	306.96	155.18	489.03	86.50	70.39	99.10	98.92	75.32	117.96	49.20	43.64	77.90	186.40	93.19	241.38	198.64	74.55	344.39	63.61	46.37	73.14
Hypertension	223.71	170.77	302.97	411.43	227.64	624.56	554.17	233.72	745.87	46.85	38.30	65.29	9.63	4.31	18.85	0.00	0.00	0.00	512.96	237.85	858.46	384.41	43.75	544.35	0.00	0.00	5.88
Migraine	14.08	10.26	19.01	11.52	6.08	19.21	8.10	0.00	12.93	21.03	15.38	29.82	15.41	8.94	19.47	12.35	8.70	23.66	54.28	15.93	74.17	75.27	4.00	144.44	28.24	20.74	37.97
Hypo or hyperthyroidism	6.85	1.67	8.44	22.91	1.67	44.59	25.44	23.20	42.48	95.17	49.91	130.33	33.79	12.78	53.26	6.43	3.92	9.84	25.38	2.44	40.64	7.69	0.00	13.04	21.22	7.62	37.02
Menorrhagia and polymenorrhoea	34.27	16.60	61.02	29.89	12.08	38.67	38.68	27.38	64.89	9.19	4.66	9.52	42.25	23.92	82.50	2.72	0.00	4.56	33.66	0.00	47.65	33.32	10.64	94.76	9.52	3.09	16.88
Asthma	37.86	25.70	40.85	26.72	25.67	38.85	22.68	17.30	33.69	18.80	16.34	20.10	25.08	18.39	25.23	41.69	28.06	65.17	18.74	14.08	30.12	17.87	9.28	34.81	38.88	31.93	45.39
Oesophagitis and oesophageal ulcer	13.58	0.00	22.40	13.25	2.44	21.26	0.00	0.00	0.00	15.85	10.85	19.20	7.29	7.77	9.20	31.99	15.91	41.25	29.76	2.07	37.50	48.19	0.00	94.06	31.25	12.93	48.92
Diabetic ophthalmic complications	71.24	48.61	92.92	82.51	47.04	111.83	63.85	10.48	79.59	19.23	8.81	28.89	15.10	0.00	29.74	0.00	0.00	0.00	84.79	54.00	132.61	34.66	0.00	50.33	26.98	0.00	58.74
Obesity	39.33	28.94	55.53	33.96	25.40	35.90	12.82	6.69	23.50	8.07	6.29	10.05	20.21	14.40	22.05	20.23	15.26	39.13	25.10	13.71	32.40	10.00	0.00	16.67	36.44	19.93	44.28
Infections of other or unspecified organs	45.70	25.16	85.72	59.10	43.42	96.03	113.09	75.64	157.94	45.62	38.16	54.58	35.78	20.19	36.05	18.36	15.06	31.65	47.66	33.91	82.97	64.08	29.91	86.60	20.66	9.53	24.65
Enthesopathies and synovial disorders	4.55	0.00	6.98	7.67	3.92	14.32	12.13	2.55	20.38	8.43	6.09	10.61	5.80	1.72	5.43	2.12	2.48	5.54	2.48	0.00	7.85	10.88	0.00	14.15	1.72	0.00	1.61
Urolithiasis	3.49	0.00	4.66	3.45	0.00	5.23	64.46	0.00	127.31	16.50	11.30	29.12	26.01	6.67	37.65	4.61	2.56	7.21	26.95	0.00	30.32	56.90	14.80	104.40	3.48	3.16	6.73
Diabetes	34.94	32.76	42.62	35.90	28.25	54.40	17.78	10.14	18.37	8.08	7.31	9.45	3.80	1.79	7.43	28.03	6.90	44.58	31.69	24.15	42.15	19.65	5.26	26.80	30.76	12.62	50.34
Diabetic neurological complications	68.64	46.10	90.73	80.19	47.04	109.72	61.41	5.24	74.33	18.83	8.81	27.98	15.10	0.00	29.74	0.00	0.00	0.00	81.91	47.37	132.61	34.66	0.00	50.33	24.96	0.00	56.93
Infection of skin and subcutaneous tissues	21.17	9.11	25.67	33.32	25.41	36.61	69.17	42.74	100.55	20.18	19.46	28.76	33.44	24.73	50.02	8.45	6.36	13.97	38.96	9.18	45.66	34.75	23.08	67.85	19.48	14.36	33.84
Ear and upper respiratory tract infections	13.20	3.99	20.84	13.22	7.61	19.96	13.29	13.44	22.27	14.33	8.94	21.44	10.54	5.20	13.09	20.47	3.78	41.17	11.85	2.36	17.21	2.17	0.00	5.77	10.27	4.85	12.40
Cholelithiasis	7.46	2.70	12.81	4.63	4.33	8.63	3.64	0.00	10.88	2.61	1.50	3.05	2.41	0.00	4.65	2.96	0.00	3.35	0.00	0.00	0.00	0.00	0.00	0.00	6.53	2.85	11.84
HIV	2.27	0.00	5.41	2.08	0.00	5.56	6.18	0.00	17.01	10.60	6.68	16.31	4.03	0.00	7.24	3.85	0.00	6.67	0.00	0.00	0.00	7.55	0.00	19.52	1.85	1.59	3.64
Lower respiratory tract infections	16.60	10.95	24.60	22.89	19.00	27.34	124.59	117.15	209.90	54.86	47.45	64.24	55.26	33.99	61.27	11.02	6.98	24.02	27.00	17.66	35.73	30.59	16.63	49.71	26.11	11.56	28.60
Gastrooesophageal reflux disease	10.27	0.00	15.24	9.98	4.51	13.30	0.00	0.00	0.00	5.65	3.04	6.63	1.65	0.00	4.31	15.90	4.52	19.33	10.64	3.33	11.11	9.74	0.00	17.08	14.66	8.16	23.73
Coronary heart disease not otherwise specified	3.03	0.00	5.88	4.09	1.22	6.48	2.16	0.00	2.84	7.37	0.83	9.57	3.89	0.00	5.74	6.25	0.00	11.76	25.40	8.70	39.51	0.00	0.00	66.67	12.04	2.04	18.36
Dysmenorrhoea	2.94	0.00	4.35	2.70	0.00	4.35	2.83	0.00	3.02	9.25	5.28	12.84	2.12	0.00	4.24	0.00	0.00	0.00	3.85	0.00	7.14	6.25	0.00	11.11	2.27	0.00	7.41
Urinary incontinence	24.63	5.08	33.11	18.57	13.67	26.76	38.20	12.91	66.89	13.74	8.90	20.58	14.47	3.49	18.03	2.94	0.00	7.50	35.61	6.30	38.55	25.02	8.33	53.06	6.30	4.44	9.25
Infections of the digestive system	14.49	8.89	29.70	30.51	23.83	42.06	84.84	43.54	114.29	19.53	17.01	27.64	28.57	20.19	40.28	6.25	0.00	15.38	45.78	9.12	53.75	49.73	9.73	82.40	27.64	13.73	34.38
Urinary tract infections	22.27	5.08	30.49	16.18	9.94	24.71	93.31	12.91	176.80	20.46	12.35	31.49	40.46	14.37	48.65	2.94	0.00	7.50	54.33	6.30	59.12	76.63	28.23	140.51	4.17	3.77	7.32
Peritonitis	2.94	0.00	3.13	6.42	3.70	6.91	24.97	5.21	44.25	15.57	9.63	17.90	5.67	1.89	10.01	4.55	4.00	8.70	17.70	2.42	24.78	18.00	2.56	41.24	6.05	4.26	9.57
Cholecystitis	7.46	2.70	12.81	4.63	4.33	8.63	7.23	0.00	10.88	3.31	2.59	3.89	4.62	2.27	8.32	2.96	0.00	3.35	0.00	0.00	0.00	0.00	0.00	0.00	9.12	4.01	11.84
Endometriosis	0.00	0.00	0.00	0.00	0.00	0.00	0.00	0.00	0.00	1.35	0.44	3.25	0.00	0.00	0.00	0.00	0.00	0.00	0.00	0.00	0.00	0.00	0.00	0.00	0.00	0.00	0.00
Ulcerative colitis	0.00	0.00	0.00	0.00	0.00	0.00	0.00	0.00	0.00	11.51	0.00	25.72	57.56	0.00	138.14	0.00	0.00	0.00	0.00	0.00	0.00	0.00	0.00	0.00	0.00	0.00	0.00
Cholangitis	7.46	2.70	12.81	4.63	4.33	8.63	10.97	0.00	11.25	5.34	3.83	7.59	8.71	4.32	13.28	2.96	0.00	3.35	0.00	0.00	0.00	0.00	0.00	0.00	11.90	4.01	15.17
Cancer (breast)	560.88	222.69	732.04	518.13	296.76	692.74	667.56	135.60	743.04	49.25	24.23	65.30	389.20	185.51	504.38	0.00	0.00	0.00	541.80	129.22	743.37	409.87	164.77	688.81	389.41	10.80	1223.24
Chronic sinusitis	16.45	14.09	27.00	13.23	7.05	21.33	0.00	0.00	0.00	5.21	4.16	7.42	6.19	1.99	10.60	25.08	2.86	40.00	10.65	2.53	17.87	0.00	0.00	0.00	14.61	0.00	18.85
Polycystic ovarian syndrome	0.00	0.00	0.00	0.00	0.00	0.00	0.00	0.00	0.00	3.75	2.09	5.01	0.00	0.00	0.00	0.00	0.00	0.00	0.00	0.00	0.00	0.00	0.00	0.00	0.00	0.00	0.00
Gastritis and duodenitis	11.79	1.72	18.33	8.74	2.33	11.76	4.91	0.00	5.49	5.67	2.95	8.76	10.10	8.95	14.21	20.28	3.74	42.76	10.33	0.00	15.90	2.22	0.00	4.55	9.92	2.13	16.13
Anorectal fistula	24.08	11.64	49.06	24.00	10.62	34.83	28.98	17.79	34.81	8.86	6.35	13.20	15.64	5.48	28.96	0.00	0.00	0.00	20.88	6.57	36.47	24.14	21.40	54.19	5.56	3.11	11.90
Postviral fatigue syndrome, neurasthenia and fibromyalgia	20.00	3.67	20.93	7.16	3.56	9.25	10.49	3.48	19.96	7.81	6.45	10.06	15.53	6.40	21.65	10.16	5.08	14.10	12.71	2.42	17.36	14.46	5.32	22.04	11.83	3.62	20.97
Hepatic failure	2.17	1.79	5.66	4.43	4.24	8.06	61.17	11.64	67.61	20.82	11.28	20.62	32.60	4.32	62.06	0.00	0.00	0.00	7.04	0.00	10.43	7.27	0.00	16.34	41.48	1.92	44.15
Viral diseases (excluding chronic hepatitis/HIV)	18.41	8.82	19.42	20.93	11.37	35.14	64.22	72.86	106.71	23.02	15.05	27.70	19.20	12.60	34.85	12.30	5.66	21.09	14.90	1.82	18.50	29.06	13.82	44.55	19.20	13.44	29.65
Chronic viral hepatitis	10.77	3.78	13.75	10.48	6.09	22.63	7.65	2.75	14.05	5.60	3.95	10.53	3.33	1.49	4.69	16.13	3.23	13.54	0.00	0.00	0.00	5.98	3.92	14.50	14.90	8.20	19.16
Pancreatitis	6.52	4.36	13.21	12.95	4.24	24.78	16.94	3.49	43.77	15.52	4.63	19.28	18.34	10.11	24.42	4.80	0.00	7.43	4.88	2.20	10.21	5.88	2.44	9.24	13.96	0.00	30.00
Other anaemias	15.67	8.33	21.58	14.84	8.57	17.02	31.11	11.63	37.55	6.29	4.74	7.51	21.87	16.25	34.95	2.94	2.56	5.56	16.42	8.73	24.47	13.24	0.00	28.36	10.64	6.95	17.16
Venous thromboembolic disease	16.58	0.00	32.56	0.00	0.00	0.00	12.37	0.00	16.73	5.89	1.31	8.57	5.90	0.00	11.51	0.00	0.00	0.00	0.00	0.00	25.00	0.00	0.00	0.00	6.59	0.00	6.95
Osteoarthritis (excluding spine)	0.00	0.00	25.00	0.00	0.00	15.38	0.00	0.00	18.75	4.68	2.52	6.55	2.41	1.94	4.18	0.00	0.00	0.00	0.00	0.00	0.00	0.00	0.00	0.00	0.00	0.00	7.14
Cancer metastasis (other organs)	65.43	22.24	97.01	355.76	258.42	532.19	742.00	574.10	1121.56	90.23	74.27	118.61	110.40	21.80	156.52	12.82	4.76	11.11	502.09	256.04	821.74	381.09	13.45	436.41	34.31	24.17	48.79
COPD	12.79	1.63	16.63	3.13	1.33	7.58	11.69	6.62	30.45	4.73	1.69	5.93	0.00	0.00	0.00	18.43	3.74	44.28	1.77	0.00	2.96	2.56	0.00	6.38	7.53	1.73	11.87
Intracranial hypertension	3.23	1.46	5.14	0.00	0.00	0.00	0.00	0.00	0.00	3.27	1.79	6.73	8.43	1.55	13.92	0.00	0.00	0.00	0.00	0.00	0.00	0.00	0.00	0.00	0.00	0.00	0.00
Septicaemia	22.61	14.47	25.84	55.11	46.83	71.11	139.56	87.73	204.41	19.90	16.20	23.60	38.58	30.04	51.69	5.54	3.68	7.74	52.38	9.78	91.79	81.54	30.82	95.63	23.64	14.63	25.86
Diverticular disease of intestine	0.00	0.00	0.00	2.11	1.86	4.24	3.36	3.07	5.21	0.39	0.00	0.81	0.00	0.00	0.00	0.00	0.00	0.00	2.47	0.00	5.29	2.63	0.00	2.63	0.00	0.00	0.00
Coeliac disease	5.09	0.00	13.53	8.27	3.29	13.08	12.42	7.58	17.25	1.75	0.83	2.18	8.33	5.09	12.45	0.00	0.00	0.00	8.90	4.12	14.12	9.38	0.00	17.86	0.00	0.00	0.00
Erectile dysfunction	2.79	0.00	6.28	2.71	0.00	7.29	4.37	0.00	6.27	9.33	4.48	12.04	0.00	0.00	0.00	4.63	0.00	7.13	8.10	3.74	15.06	10.60	5.00	13.41	0.00	0.00	0.00
Epilepsy	17.36	9.76	24.56	9.56	0.00	16.77	10.30	0.00	11.25	9.95	9.23	12.19	7.81	2.32	10.63	1.69	0.00	5.33	9.97	3.64	14.28	22.25	4.63	25.43	15.71	7.16	29.18
Fatty liver	4.29	2.33	10.47	6.55	4.24	10.45	11.57	5.21	14.38	5.32	2.43	6.28	7.59	5.59	11.32	2.72	0.00	5.18	4.94	2.20	7.83	2.63	0.00	2.63	11.70	4.47	14.11
Nonrheumatic mitral valve disorders	0.00	0.00	0.00	0.00	0.00	0.00	0.00	0.00	0.00	8.83	0.38	12.11	0.00	0.00	0.00	0.00	0.00	0.00	0.00	0.00	0.00	0.00	0.00	0.00	0.00	0.00	0.00
Heart failure	0.00	0.00	0.00	0.00	0.00	0.00	15.58	0.00	37.64	17.18	10.87	20.09	11.96	0.00	23.90	0.00	0.00	0.00	11.41	0.00	12.42	12.69	0.00	34.72	0.00	0.00	0.00
Hypertrophy of nasal turbinates	11.99	3.99	20.54	8.68	0.00	13.01	0.00	0.00	0.00	1.46	0.50	1.86	0.00	0.00	0.00	20.47	3.78	41.17	10.21	2.02	17.21	2.17	0.00	5.77	6.92	0.00	11.79
Peripheral neuropathies	1.62	0.00	3.93	4.70	2.49	6.06	5.47	4.31	8.68	4.11	2.69	5.16	0.00	0.00	0.00	2.13	0.00	4.55	5.95	3.22	11.74	4.35	0.00	5.26	2.08	0.00	3.89
Cancer metastasis (retroperitoneum and peritoneum	27.30	9.77	35.33	317.37	234.65	503.73	659.93	539.55	1026.12	74.86	57.46	107.13	74.96	16.10	104.91	12.82	4.76	11.11	463.47	193.28	817.59	358.74	13.45	411.46	28.61	18.10	48.79
Cancer metastasis (pleura)	27.30	9.77	35.33	317.37	234.65	503.73	659.93	539.55	1026.12	74.86	57.46	107.13	74.96	16.10	104.91	12.82	4.76	11.11	463.47	193.28	817.59	358.74	13.45	411.46	28.61	18.10	48.79
Cancer metastasis (lung)	95.63	15.02	144.09	386.13	282.35	630.75	738.95	545.27	1123.51	77.13	59.81	108.77	85.96	21.94	104.91	12.82	4.76	11.11	521.06	198.41	868.81	413.21	71.99	485.27	171.06	18.10	399.99
Cancer metastasis (liver and intrahepatic bile duct)	44.31	15.02	59.87	334.76	246.67	515.67	683.34	539.55	1057.39	74.86	57.46	107.13	74.96	16.10	104.91	12.82	4.76	11.11	477.21	193.28	830.53	372.33	38.66	430.01	63.73	18.10	136.59
Cancer metastasis (bowel)	27.30	9.77	35.33	317.37	234.65	503.73	659.93	539.55	1026.12	74.86	57.46	107.13	74.96	16.10	104.91	12.82	4.76	11.11	463.47	193.28	817.59	358.74	13.45	411.46	28.61	18.10	48.79
Meningitis	11.66	9.27	16.38	13.44	10.96	20.33	19.96	12.98	27.42	11.93	9.36	14.34	10.41	8.19	15.95	3.79	3.47	6.35	5.86	2.55	9.18	10.01	0.00	18.85	7.71	5.51	10.98
Other cardiomyopathy	0.00	0.00	0.00	1.33	0.00	4.00	2.16	0.00	2.84	11.31	7.54	15.11	0.00	0.00	0.00	0.00	0.00	0.00	0.00	0.00	0.00	0.00	0.00	0.00	0.00	0.00	0.00
Infections of the heart	4.08	0.00	8.69	6.30	5.05	9.31	12.45	5.21	19.87	8.80	5.98	9.43	6.71	1.76	11.02	1.69	0.00	3.39	5.86	2.55	9.18	6.91	0.00	15.60	1.66	0.00	3.60
Peripheral arterial disease	2.27	0.00	2.38	2.08	0.00	2.27	0.00	0.00	0.00	1.50	0.38	1.54	3.69	0.00	7.33	3.85	3.33	8.00	2.86	0.00	5.71	0.00	0.00	0.00	1.85	1.61	4.44
Female infertility	0.00	0.00	0.00	0.00	0.00	0.00	0.00	0.00	0.00	2.18	1.88	3.73	0.00	0.00	0.00	0.00	0.00	0.00	0.00	0.00	0.00	0.00	0.00	0.00	0.00	0.00	0.00
Cancer metastasis (lymph nodes)	66.51	45.68	66.55	67.25	50.63	82.58	217.20	29.99	372.17	45.76	22.95	89.76	14.47	3.66	23.16	7.52	0.00	7.98	162.45	64.88	177.31	129.35	18.82	309.75	16.61	10.03	34.74
Cancer (leukaemia)	171.93	25.78	285.71	296.59	156.22	414.17	481.91	464.38	756.67	256.65	179.08	339.52	208.08	176.95	352.12	0.00	0.00	0.00	166.66	83.33	414.66	0.00	0.00	0.00	254.88	181.50	408.34
Pneumothorax	0.00	0.00	0.00	0.00	0.00	0.00	0.00	0.00	0.00	2.46	1.20	2.26	0.00	0.00	4.17	0.00	0.00	0.00	0.00	0.00	0.00	0.00	0.00	0.00	0.00	0.00	0.00
Acute kidney injury	6.06	0.00	7.69	13.49	7.96	23.52	48.37	23.02	76.45	6.25	4.28	7.61	17.80	0.00	29.73	0.00	0.00	0.00	8.85	0.00	21.03	30.71	0.00	47.06	5.98	0.00	9.28
Cancer (brain, other cns and intracranial)	150.90	66.96	194.14	0.00	0.00	0.00	0.00	0.00	0.00	56.25	44.06	98.70	3.52	0.00	6.27	0.00	0.00	0.00	69.84	0.00	121.11	144.91	15.38	301.64	52.30	8.65	72.96
Pulmonary embolism	0.00	0.00	0.00	0.00	0.00	0.00	3.64	0.00	8.20	0.81	0.37	1.15	0.00	0.00	0.00	0.00	0.00	0.00	0.00	0.00	0.00	0.00	0.00	0.00	0.00	0.00	0.00
Mycoses	4.08	0.00	8.69	6.30	5.05	9.31	12.45	5.21	19.87	8.86	5.71	11.26	6.71	1.76	11.02	1.69	0.00	3.39	5.86	2.55	9.18	6.91	0.00	15.60	3.47	0.00	5.63
Portal hypertension	2.17	1.79	5.66	4.43	4.24	8.06	11.57	5.21	14.38	5.32	2.43	6.28	5.37	3.13	9.95	0.00	0.00	0.00	2.47	0.00	5.29	2.63	0.00	2.63	8.87	1.92	9.77
Raynaud's syndrome	0.00	0.00	0.00	0.00	0.00	0.00	0.00	0.00	0.00	0.96	0.38	1.15	1.93	0.00	3.63	0.00	0.00	0.00	0.00	0.00	0.00	0.00	0.00	0.00	0.00	0.00	0.00
Cancer (other skin and subcutaneous tissue)	0.00	0.00	0.00	0.00	0.00	0.00	0.00	0.00	0.00	7.56	3.18	12.19	23.50	0.00	46.16	0.00	0.00	0.00	0.00	0.00	0.00	0.00	0.00	0.00	0.00	0.00	0.00
Sleep apnoea	8.41	0.00	13.12	8.46	0.00	11.40	0.00	0.00	0.00	1.97	1.08	3.66	0.00	0.00	0.00	9.03	6.95	28.02	16.08	11.03	21.47	5.59	0.00	11.76	0.00	0.00	0.00
Male infertility	0.00	0.00	0.00	0.00	0.00	0.00	0.00	0.00	0.00	5.08	3.22	10.43	0.00	0.00	0.00	0.00	0.00	0.00	0.00	0.00	0.00	0.00	0.00	0.00	0.00	0.00	0.00
Cancer (testicular)	0.00	0.00	0.00	0.00	0.00	0.00	0.00	0.00	0.00	33.39	19.43	71.71	1.93	1.65	3.59	23.33	29.45	53.37	19.32	2.55	56.90	30.29	3.57	48.37	0.00	0.00	0.00
Eye infections	7.89	3.53	11.43	7.61	5.10	12.14	10.66	5.70	13.29	6.17	5.00	12.93	3.97	3.49	8.01	2.12	1.92	5.54	5.00	0.00	9.09	13.00	3.57	29.84	8.82	6.67	12.85
Bell's palsy	0.00	0.00	0.00	0.00	0.00	0.00	0.00	0.00	0.00	3.80	0.64	6.48	0.00	0.00	0.00	0.00	0.00	0.00	0.00	0.00	0.00	0.00	0.00	0.00	0.00	0.00	0.00
Cancer (colorectal and anus)	0.00	0.00	0.00	0.00	0.00	0.00	79.95	0.00	170.67	7.12	1.49	18.91	0.00	0.00	0.00	0.00	0.00	0.00	38.56	0.00	43.89	85.69	34.78	209.61	0.00	0.00	0.00
End stage renal disease	30.91	20.97	39.87	31.13	22.11	32.30	42.33	32.33	56.43	19.37	18.49	22.75	30.08	19.78	40.18	11.14	4.76	11.07	18.25	12.07	25.08	17.57	10.81	22.59	22.13	12.63	21.10
Hypertrophic cardiomyopathy	0.00	0.00	0.00	0.00	0.00	0.00	0.00	0.00	0.00	4.10	1.87	9.56	0.00	0.00	0.00	0.00	0.00	0.00	0.00	0.00	0.00	0.00	0.00	0.00	0.00	0.00	0.00
Pleural effusion	20.08	4.32	27.30	22.14	11.03	43.66	18.78	12.92	28.96	6.23	3.82	8.05	8.61	4.93	16.62	16.83	10.47	24.93	24.29	14.22	35.24	12.76	0.00	15.59	30.54	17.62	48.37
Myocardial infarction	0.00	0.00	0.00	1.33	0.00	4.00	2.16	0.00	2.84	5.47	0.47	7.20	0.00	0.00	0.00	0.00	0.00	0.00	0.00	0.00	0.00	0.00	0.00	0.00	0.00	0.00	0.00
Dilated cardiomyopathy	0.00	0.00	0.00	0.00	0.00	0.00	0.00	0.00	0.00	5.58	3.39	10.98	0.00	0.00	0.00	0.00	0.00	0.00	0.00	0.00	0.00	0.00	0.00	0.00	0.00	0.00	0.00
Alcoholic liver disease	0.00	0.00	0.00	0.00	0.00	0.00	0.00	0.00	10.00	2.63	0.66	2.87	0.00	0.00	7.69	0.00	0.00	0.00	0.00	0.00	0.00	0.00	0.00	0.00	0.00	0.00	7.69
Right bundle branch block	0.00	0.00	0.00	0.00	0.00	0.00	0.00	0.00	0.00	0.00	0.00	0.00	0.00	0.00	0.00	0.00	0.00	0.00	0.00	0.00	0.00	0.00	0.00	0.00	0.00	0.00	0.00
Bifascicular block	0.00	0.00	0.00	0.00	0.00	0.00	0.00	0.00	0.00	0.00	0.00	0.00	0.00	0.00	0.00	0.00	0.00	0.00	0.00	0.00	0.00	0.00	0.00	0.00	0.00	0.00	0.00
Supraventricular tachycardia	0.00	0.00	0.00	0.00	0.00	0.00	0.00	0.00	0.00	3.68	0.88	4.58	0.00	0.00	0.00	0.00	0.00	0.00	0.00	0.00	0.00	0.00	0.00	0.00	0.00	0.00	0.00
Oesophageal varices	0.00	0.00	0.00	0.00	0.00	0.00	51.37	0.00	62.20	9.28	0.00	9.60	31.84	0.00	62.19	0.00	0.00	0.00	0.00	0.00	0.00	0.00	0.00	0.00	39.21	0.00	45.34
Ventricular tachycardia	0.00	0.00	0.00	0.00	0.00	0.00	0.00	0.00	0.00	3.68	0.88	4.58	0.00	0.00	0.00	0.00	0.00	0.00	0.00	0.00	0.00	0.00	0.00	0.00	0.00	0.00	0.00
Glomerulonephritis	0.00	0.00	0.00	0.00	0.00	0.00	0.00	0.00	0.00	2.31	0.00	2.34	0.00	0.00	0.00	0.00	0.00	0.00	0.00	0.00	0.00	0.00	0.00	0.00	0.00	0.00	0.00
Barrett's oesophagus	5.09	0.00	11.20	5.00	0.00	7.18	0.00	0.00	0.00	5.60	3.52	6.16	7.29	7.77	9.20	7.52	0.00	7.98	0.00	0.00	0.00	0.00	0.00	0.00	8.30	0.00	13.53
Obstructive and reflux uropathy	0.00	0.00	0.00	0.00	0.00	0.00	64.46	0.00	127.31	7.67	2.74	13.94	23.87	0.00	35.31	0.00	0.00	0.00	23.57	0.00	25.96	56.90	14.80	104.40	0.00	0.00	0.00
Infection of anal and rectal regions	0.00	0.00	0.00	0.00	0.00	0.00	0.00	0.00	0.00	4.29	0.79	6.94	0.00	0.00	0.00	0.00	0.00	0.00	0.00	0.00	0.00	0.00	0.00	0.00	0.00	0.00	0.00
Disorders of autonomic nervous system	5.29	0.00	8.11	2.08	0.00	2.27	0.00	0.00	0.00	0.38	0.00	0.77	1.77	0.00	5.23	3.85	3.33	8.00	6.29	0.00	9.37	7.69	0.00	13.04	3.93	2.04	6.71
Nasal polyp	10.00	0.00	18.18	6.67	0.00	9.09	0.00	0.00	0.00	0.35	0.00	0.70	0.00	0.00	0.00	16.67	0.00	40.00	8.33	0.00	14.29	0.00	0.00	0.00	4.00	0.00	6.90
Trifascicular block	0.00	0.00	0.00	0.00	0.00	0.00	0.00	0.00	0.00	0.00	0.00	0.00	0.00	0.00	0.00	0.00	0.00	0.00	0.00	0.00	0.00	0.00	0.00	0.00	0.00	0.00	0.00
Nonrheumatic aortic valve disorders	0.00	0.00	0.00	1.33	0.00	4.00	2.16	0.00	2.84	5.22	0.00	10.57	0.00	0.00	0.00	0.00	0.00	0.00	0.00	0.00	0.00	0.00	0.00	0.00	0.00	0.00	0.00
Anterior and intermediate uveitis	0.00	0.00	0.00	0.00	0.00	0.00	0.00	0.00	0.00	1.17	1.08	2.40	0.00	0.00	0.00	0.00	0.00	0.00	0.00	0.00	0.00	0.00	0.00	0.00	0.00	0.00	0.00
Pulmonary collapse (excluding pneumothorax)	9.47	2.33	13.99	6.92	0.00	11.84	10.08	2.61	16.28	10.56	9.19	12.41	5.70	0.00	5.97	0.00	0.00	0.00	8.82	2.86	13.45	6.45	0.00	10.00	16.61	0.00	40.15
Stable angina	0.00	0.00	0.00	0.00	0.00	0.00	4.37	0.00	11.07	0.54	0.00	1.48	0.00	0.00	0.00	0.00	0.00	0.00	3.23	0.00	3.76	3.85	0.00	11.11	0.00	0.00	0.00
Stroke(not otherwise specified)	0.00	0.00	0.00	53.58	0.00	56.93	75.68	0.00	165.05	2.16	0.00	5.91	0.00	0.00	0.00	0.00	0.00	0.00	68.74	0.00	143.54	0.00	0.00	0.00	0.00	0.00	0.00
Atrial fibrillation	0.00	0.00	0.00	0.00	0.00	0.00	0.00	0.00	0.00	1.52	0.71	1.69	0.00	0.00	0.00	0.00	0.00	0.00	0.00	0.00	0.00	0.00	0.00	0.00	0.00	0.00	0.00
Endometrial hyperplasia and hypertrophy	0.00	0.00	0.00	0.00	0.00	0.00	0.00	0.00	0.00	1.33	0.67	1.41	3.13	0.00	6.06	0.00	0.00	0.00	0.00	0.00	0.00	0.00	0.00	0.00	0.00	0.00	0.00
Cancer (non-hodgkin lymphoma)	18.94	7.57	30.16	17.05	12.41	26.32	19.37	10.49	21.17	141.28	69.07	203.96	135.64	17.92	136.91	10.51	6.53	14.74	5.45	0.00	8.57	5.59	2.70	10.34	16.16	10.78	38.31
Multiple valve disease	0.00	0.00	0.00	0.00	0.00	0.00	0.00	0.00	0.00	1.40	0.00	3.37	0.00	0.00	0.00	0.00	0.00	0.00	0.00	0.00	0.00	0.00	0.00	0.00	0.00	0.00	0.00
Cancer metastasis (brain, cns and intracranial)	32.82	14.06	46.40	24.12	8.99	44.90	36.39	14.66	42.42	52.37	28.02	86.07	92.32	14.16	137.12	0.00	0.00	0.00	35.97	9.77	45.34	22.92	9.09	30.95	22.24	12.49	40.15
Cancer metastasis (bone)	32.82	14.06	46.40	24.12	8.99	44.90	36.39	14.66	42.42	53.64	28.75	89.08	96.65	14.16	144.67	0.00	0.00	0.00	35.97	9.77	45.34	22.92	9.09	30.95	22.24	12.49	40.15
Cancer metastasis (adrenal gland)	32.82	14.06	46.40	24.12	8.99	44.90	36.39	14.66	42.42	51.99	28.02	85.69	92.32	14.16	137.12	0.00	0.00	0.00	35.97	9.77	45.34	22.92	9.09	30.95	22.24	12.49	40.15
Crohn's disease	0.00	0.00	0.00	0.00	0.00	0.00	0.00	0.00	0.00	1.22	0.33	2.51	0.00	0.00	0.00	0.00	0.00	0.00	0.00	0.00	0.00	0.00	0.00	0.00	0.00	0.00	0.00
Cataract	0.00	0.00	0.00	0.00	0.00	0.00	5.27	0.00	4.78	8.22	4.72	12.74	0.00	0.00	0.00	0.00	0.00	0.00	0.00	0.00	0.00	0.00	0.00	0.00	0.00	0.00	0.00
Cancer (cervical)	0.00	0.00	0.00	0.00	0.00	0.00	0.00	0.00	0.00	0.40	0.00	0.76	0.00	0.00	0.00	0.00	0.00	0.00	0.00	0.00	0.00	5.77	0.00	6.00	0.00	0.00	0.00
Thrombophilia	0.00	0.00	0.00	0.00	0.00	0.00	0.00	0.00	0.00	1.29	0.39	2.16	0.00	0.00	0.00	0.00	0.00	0.00	0.00	0.00	0.00	0.00	0.00	0.00	0.00	0.00	0.00
Encephalitis	7.89	3.53	11.43	7.61	5.10	12.14	8.65	5.24	10.71	5.94	3.93	9.69	3.97	3.49	8.01	2.12	1.92	5.54	0.00	0.00	0.00	3.33	0.00	8.33	6.13	3.85	7.78
Agranulocytosis	24.76	9.34	36.02	33.98	21.06	44.01	60.06	31.62	76.80	13.81	11.93	14.70	29.80	25.91	40.37	5.95	3.49	9.88	7.56	1.69	18.61	35.94	15.16	42.95	30.05	11.54	34.93
Subarachnoid haemorrhage	0.00	0.00	0.00	0.00	0.00	0.00	0.00	0.00	0.00	2.16	0.00	5.91	0.00	0.00	0.00	0.00	0.00	0.00	0.00	0.00	0.00	0.00	0.00	0.00	0.00	0.00	0.00
Transient ischaemic attack	5.09	0.00	9.67	5.00	0.00	7.18	0.00	0.00	0.00	0.00	0.00	0.00	0.00	0.00	0.00	0.00	0.00	0.00	4.94	0.00	8.31	0.00	0.00	0.00	0.00	0.00	0.00
Rheumatoid arthritis	0.00	0.00	0.00	0.00	0.00	0.00	0.00	0.00	0.00	0.00	0.00	0.00	0.00	0.00	0.00	0.00	0.00	0.00	0.00	0.00	0.00	0.00	0.00	0.00	0.00	0.00	0.00
Liver fibrosis sclerosis and cirrhosis	0.00	0.00	0.00	0.00	0.00	0.00	4.44	0.00	4.76	4.08	1.42	6.21	2.63	0.00	4.88	0.00	0.00	0.00	0.00	0.00	0.00	0.00	0.00	0.00	3.33	0.00	3.85
Ischaemic stroke	0.00	0.00	0.00	0.00	0.00	0.00	0.00	0.00	0.00	4.15	1.08	9.46	0.00	0.00	0.00	0.00	0.00	0.00	0.00	0.00	0.00	0.00	0.00	0.00	0.00	0.00	0.00
Left bundle branch block	0.00	0.00	0.00	0.00	0.00	0.00	2.16	0.00	5.38	2.10	0.00	6.45	0.00	0.00	0.00	0.00	0.00	0.00	0.00	0.00	0.00	0.00	0.00	0.00	0.00	0.00	0.00
Scoliosis	0.00	0.00	0.00	0.00	0.00	0.00	0.00	0.00	0.00	1.73	1.11	2.12	0.00	0.00	0.00	0.00	0.00	0.00	0.00	0.00	0.00	0.00	0.00	0.00	0.00	0.00	0.00
Infection of liver	6.75	0.00	11.89	19.07	10.17	36.11	28.33	27.71	41.03	5.27	2.39	7.67	0.00	0.00	0.00	7.62	6.86	19.05	13.37	0.00	31.86	24.13	0.00	29.03	7.46	0.00	14.43
Atrioventricular block, second degree	0.00	0.00	0.00	0.00	0.00	0.00	2.16	0.00	5.38	0.70	0.00	2.15	0.00	0.00	0.00	0.00	0.00	0.00	0.00	0.00	0.00	0.00	0.00	0.00	0.00	0.00	0.00
Atrioventricular block, first degree	0.00	0.00	0.00	0.00	0.00	0.00	2.16	0.00	5.38	0.70	0.00	2.15	0.00	0.00	0.00	0.00	0.00	0.00	0.00	0.00	0.00	0.00	0.00	0.00	0.00	0.00	0.00
Atrioventricular block, complete	0.00	0.00	0.00	0.00	0.00	0.00	2.16	0.00	5.38	0.70	0.00	2.15	0.00	0.00	0.00	0.00	0.00	0.00	0.00	0.00	0.00	0.00	0.00	0.00	0.00	0.00	0.00
Infection of bones and joints	0.00	0.00	0.00	0.00	0.00	0.00	0.00	0.00	0.00	2.79	1.93	3.72	1.87	0.00	3.51	0.00	0.00	0.00	0.00	0.00	0.00	0.00	0.00	0.00	0.00	0.00	0.00
Cancer (thyroid)	0.00	0.00	0.00	0.00	0.00	0.00	0.00	0.00	0.00	1.09	0.00	2.27	11.79	5.00	22.61	0.00	0.00	0.00	0.00	0.00	0.00	0.00	0.00	0.00	0.00	0.00	0.00
Glaucoma	0.00	0.00	0.00	0.00	0.00	0.00	0.00	0.00	0.00	0.00	0.00	0.00	0.00	0.00	0.00	0.00	0.00	0.00	0.00	0.00	0.00	0.00	0.00	0.00	0.00	0.00	0.00
Secondary or other thrombocytopaenia	16.58	8.04	23.52	12.12	9.18	19.37	30.34	21.24	39.06	4.43	1.72	5.63	9.54	6.69	12.99	0.00	0.00	0.00	7.32	0.00	18.91	7.73	0.00	10.64	8.98	5.51	12.15
Enteropathic arthropathy	0.00	0.00	0.00	0.00	0.00	0.00	0.00	0.00	0.00	0.40	0.00	0.89	2.28	0.00	5.96	0.00	0.00	0.00	0.00	0.00	0.00	0.00	0.00	0.00	0.00	0.00	0.00
Non-acute cystitis	0.00	0.00	0.00	0.00	0.00	0.00	0.00	0.00	0.00	0.43	0.00	0.89	1.87	0.00	2.12	0.00	0.00	0.00	0.00	0.00	0.00	0.00	0.00	0.00	0.00	0.00	0.00
Sick sinus syndrome	0.00	0.00	0.00	0.00	0.00	0.00	0.00	0.00	0.00	0.81	0.00	1.58	0.00	0.00	0.00	0.00	0.00	0.00	0.00	0.00	0.00	0.00	0.00	0.00	0.00	0.00	0.00
Essential tremor	0.00	0.00	0.00	0.00	0.00	0.00	0.00	0.00	0.00	4.08	0.00	8.36	0.00	0.00	0.00	0.00	0.00	0.00	0.00	0.00	0.00	0.00	0.00	0.00	0.00	0.00	0.00
Primary or idiopathic thrombocytopaenia	16.58	8.04	23.52	12.12	9.18	19.37	30.34	21.24	39.06	4.04	1.51	5.33	9.54	6.69	12.99	0.00	0.00	0.00	7.32	0.00	18.91	7.73	0.00	10.64	8.98	5.51	12.15
Respiratory failure	1.32	0.00	2.59	1.33	0.00	2.71	3.59	0.00	9.17	6.58	3.42	6.64	5.98	0.00	7.78	1.69	0.00	3.39	4.35	3.33	9.52	0.00	0.00	0.00	3.47	0.00	5.63
Spinal stenosis	0.00	0.00	0.00	0.00	0.00	0.00	0.00	0.00	0.00	2.07	0.00	2.31	0.00	0.00	0.00	0.00	0.00	0.00	0.00	0.00	0.00	0.00	0.00	0.00	0.00	0.00	0.00
Multiple sclerosis	0.00	0.00	0.00	0.00	0.00	0.00	0.00	0.00	0.00	0.00	0.00	0.00	0.00	0.00	0.00	0.00	0.00	0.00	0.00	0.00	0.00	0.00	0.00	0.00	0.00	0.00	0.00
Pericardial effusion (noninflammatory)	0.00	0.00	0.00	0.00	0.00	0.00	0.00	0.00	0.00	5.51	2.84	6.97	0.00	0.00	0.00	0.00	0.00	0.00	0.00	0.00	0.00	0.00	0.00	0.00	0.00	0.00	0.00
Intracerebral haemorrhage	2.94	0.00	5.13	0.00	0.00	0.00	0.00	0.00	0.00	2.16	0.00	5.91	0.00	0.00	0.00	0.00	0.00	0.00	0.00	0.00	0.00	0.00	0.00	0.00	0.00	0.00	0.00
Cancer (malignant melanoma)	77.80	0.00	102.66	77.47	0.00	121.22	111.26	0.00	144.39	26.64	0.00	57.11	0.00	0.00	0.00	0.00	0.00	0.00	83.23	0.00	151.43	84.55	0.00	146.00	118.34	0.00	412.52
Osteoporosis	0.00	0.00	0.00	0.00	0.00	0.00	2.92	0.00	4.71	2.49	1.26	2.96	2.04	0.00	4.08	0.00	0.00	0.00	0.00	0.00	0.00	0.00	0.00	0.00	1.95	0.00	2.08
Lupus erythematosus (local and systemic)	0.00	0.00	0.00	0.00	0.00	0.00	0.00	0.00	0.00	0.00	0.00	0.00	0.00	0.00	0.00	0.00	0.00	0.00	0.00	0.00	0.00	0.00	0.00	0.00	0.00	0.00	0.00
Unstable angina	0.00	0.00	0.00	0.00	0.00	0.00	4.37	0.00	11.07	0.54	0.00	1.48	0.00	0.00	0.00	0.00	0.00	0.00	3.23	0.00	3.76	3.85	0.00	11.11	0.00	0.00	0.00
Autoimmune liver disease	0.00	0.00	0.00	3.17	2.49	5.45	8.98	4.91	11.85	2.15	0.61	3.84	2.26	0.00	4.43	0.00	0.00	0.00	3.91	0.00	9.78	4.35	0.00	5.26	2.78	0.00	3.03
Cancer (other organs)	96.79	33.08	154.64	89.34	41.14	157.53	559.51	3.48	987.13	25.35	13.49	42.55	5.53	1.90	10.26	0.00	0.00	0.00	104.72	20.34	152.14	133.52	46.61	186.92	73.53	5.88	254.31
Hyperparathyroidism	0.00	0.00	0.00	0.00	0.00	0.00	0.00	0.00	0.00	1.41	0.00	2.52	0.00	0.00	0.00	0.00	0.00	0.00	0.00	0.00	0.00	0.00	0.00	0.00	0.00	0.00	0.00
Macular degeneration	0.00	0.00	0.00	0.00	0.00	0.00	0.00	0.00	0.00	0.00	0.00	0.00	0.00	0.00	0.00	0.00	0.00	0.00	0.00	0.00	0.00	0.00	0.00	0.00	0.00	0.00	0.00
Ankylosing spondylitis	0.00	0.00	0.00	0.00	0.00	0.00	0.00	0.00	0.00	0.61	0.00	1.24	0.00	0.00	0.00	0.00	0.00	0.00	0.00	0.00	0.00	0.00	0.00	0.00	0.00	0.00	0.00
Secondary polycythaemia	5.09	0.00	13.53	5.00	0.00	13.30	6.83	0.00	9.26	1.15	0.37	1.49	1.87	0.00	3.51	0.00	0.00	0.00	4.94	0.00	16.68	5.00	0.00	5.88	0.00	0.00	0.00
Abdominal aortic aneurysm	0.00	0.00	0.00	0.00	0.00	0.00	0.00	0.00	0.00	0.46	0.00	0.95	0.00	0.00	0.00	0.00	0.00	0.00	0.00	0.00	0.00	0.00	0.00	0.00	0.00	0.00	0.00
Aplastic anaemias	2.17	0.00	3.57	0.00	0.00	0.00	3.73	0.00	4.20	1.82	0.84	3.08	2.26	0.00	4.43	0.00	0.00	0.00	0.00	0.00	0.00	0.00	0.00	0.00	2.78	0.00	3.03
Cancer (stomach)	20.38	0.00	44.79	19.99	0.00	28.72	0.00	0.00	0.00	0.40	0.00	0.45	0.00	0.00	0.00	30.09	0.00	31.94	0.00	0.00	0.00	0.00	0.00	0.00	33.22	0.00	54.12
Primary pulmonary hypertension	0.00	0.00	0.00	0.00	0.00	0.00	0.00	0.00	0.00	0.00	0.00	0.00	0.00	0.00	0.00	0.00	0.00	0.00	0.00	0.00	0.00	0.00	0.00	0.00	0.00	0.00	0.00
Secondary pulmonary hypertension	0.00	0.00	0.00	0.00	0.00	0.00	0.00	0.00	0.00	0.00	0.00	0.00	0.00	0.00	0.00	0.00	0.00	0.00	0.00	0.00	0.00	0.00	0.00	0.00	0.00	0.00	0.00
Angiodysplasia of colon	0.00	0.00	0.00	0.00	0.00	0.00	0.00	0.00	0.00	0.39	0.00	0.77	0.00	0.00	0.00	0.00	0.00	0.00	0.00	0.00	0.00	0.00	0.00	0.00	0.00	0.00	0.00
Bronchiectasis	0.00	0.00	0.00	0.00	0.00	0.00	0.00	0.00	0.00	0.82	0.00	1.24	0.00	0.00	0.00	0.00	0.00	0.00	0.00	0.00	0.00	0.00	0.00	0.00	0.00	0.00	0.00
Dementia	0.00	0.00	0.00	3.17	2.49	5.45	8.13	5.13	14.11	0.00	0.00	0.00	0.00	0.00	0.00	0.00	0.00	0.00	3.91	0.00	9.78	4.35	0.00	5.26	0.00	0.00	0.00
Other haemolytic anaemias	0.00	0.00	0.00	2.78	0.00	5.71	2.75	0.00	5.91	0.54	0.00	1.13	0.00	0.00	0.00	6.25	4.76	12.50	3.45	0.00	4.00	0.00	0.00	0.00	0.00	0.00	0.00
Systemic sclerosis	0.00	0.00	0.00	0.00	0.00	0.00	0.00	0.00	0.00	0.55	0.51	1.13	0.00	0.00	0.00	0.00	0.00	0.00	0.00	0.00	0.00	0.00	0.00	0.00	0.00	0.00	0.00
MGUS	0.00	0.00	0.00	0.00	0.00	0.00	2.83	0.00	7.72	0.55	0.00	1.54	0.00	0.00	0.00	0.00	0.00	0.00	0.00	0.00	0.00	0.00	0.00	0.00	0.00	0.00	0.00
Immunodeficiencies	0.00	0.00	0.00	0.00	0.00	0.00	0.00	0.00	0.00	1.46	0.37	3.02	0.00	0.00	0.00	0.00	0.00	0.00	0.00	0.00	0.00	0.00	0.00	0.00	0.00	0.00	0.00
Cancer (hodgkin lymphoma)	254.78	133.70	236.98	219.46	90.90	294.51	695.37	133.56	1160.95	103.73	25.65	116.13	512.84	139.45	904.90	243.80	195.36	354.18	161.30	35.68	226.13	593.78	97.27	1026.22	234.35	179.14	297.85
Cancer (ovarian)	3.03	0.00	5.88	2.78	0.00	6.25	0.00	0.00	0.00	0.42	0.00	1.24	2.12	0.00	1.88	0.00	0.00	0.00	3.45	0.00	6.25	0.00	0.00	0.00	0.00	0.00	0.00
Cancer (myelodysplastic syndromes)	15.15	0.00	18.52	13.89	0.00	15.63	13.73	0.00	25.51	32.50	0.00	65.37	9.94	0.00	28.30	0.00	0.00	0.00	0.00	0.00	0.00	0.00	0.00	0.00	10.42	0.00	19.23
Posterior uveitis	0.00	0.00	0.00	0.00	0.00	0.00	0.00	0.00	0.00	0.00	0.00	0.00	0.00	0.00	0.00	0.00	0.00	0.00	0.00	0.00	0.00	0.00	0.00	0.00	0.00	0.00	0.00
Cancer (bone and articular cartilage)	34.87	22.71	68.85	123.50	0.00	233.52	126.70	20.85	155.33	40.51	3.35	71.16	235.04	0.00	222.00	37.53	0.00	89.19	58.25	21.33	104.17	299.98	9.67	326.55	10.70	0.00	20.13
Cancer (oro-pharyngeal)	0.00	0.00	0.00	0.00	0.00	0.00	0.00	0.00	0.00	1.67	0.41	2.89	0.00	0.00	0.00	0.00	0.00	0.00	0.00	0.00	0.00	42.22	0.00	86.36	0.00	0.00	0.00
Other interstitial pulmonary diseases with fibrosis	0.00	0.00	0.00	0.00	0.00	0.00	0.00	0.00	0.00	0.00	0.00	0.00	0.00	0.00	0.00	0.00	0.00	0.00	0.00	0.00	0.00	0.00	0.00	0.00	0.00	0.00	0.00
Cancer (lung and trachea)	0.00	0.00	0.00	0.00	0.00	0.00	0.00	0.00	0.00	11.54	5.23	15.36	0.00	0.00	4.17	0.00	0.00	0.00	0.00	0.00	0.00	0.00	0.00	0.00	0.00	0.00	0.00
Cancer (bladder)	0.00	0.00	0.00	0.00	0.00	0.00	0.00	0.00	0.00	0.00	0.00	0.00	0.00	0.00	0.00	0.00	0.00	0.00	0.00	0.00	0.00	0.00	0.00	0.00	0.00	0.00	0.00
Cancer (uterine	0.00	0.00	0.00	0.00	0.00	0.00	0.00	0.00	0.00	0.00	0.00	0.00	0.00	0.00	0.00	0.00	0.00	0.00	0.00	0.00	0.00	0.00	0.00	0.00	0.00	0.00	0.00
Cancer (pancreatic)	0.00	0.00	0.00	242.58	180.75	391.71	318.91	245.26	535.51	0.38	0.00	0.77	1.87	0.00	3.51	0.00	0.00	0.00	241.95	0.00	522.22	248.81	0.00	300.19	0.00	0.00	0.00
Cancer (oesophageal)	15.28	0.00	33.59	14.99	0.00	21.54	0.00	0.00	0.00	0.00	0.00	0.00	0.00	0.00	0.00	22.57	0.00	23.95	0.00	0.00	0.00	0.00	0.00	0.00	24.91	0.00	40.59
Cancer (multiple myeloma and malignant plasma cell neoplasms)	0.00	0.00	0.00	0.00	0.00	0.00	0.00	0.00	0.00	0.00	0.00	0.00	0.00	0.00	0.00	0.00	0.00	0.00	0.00	0.00	0.00	0.00	0.00	0.00	0.00	0.00	0.00
Cancer (multiple independent sites)	0.00	0.00	0.00	0.00	0.00	0.00	0.00	0.00	0.00	0.00	0.00	0.00	0.00	0.00	0.00	0.00	0.00	0.00	0.00	0.00	0.00	0.00	0.00	0.00	0.00	0.00	0.00
Cancer (liver)	0.00	0.00	0.00	0.00	0.00	0.00	0.00	0.00	0.00	0.54	0.54	1.14	0.00	0.00	0.00	0.00	0.00	0.00	0.00	0.00	0.00	0.00	0.00	0.00	0.00	0.00	0.00
Cancer (kidney and ureter)	0.00	0.00	0.00	0.00	0.00	0.00	22.64	0.00	53.18	3.41	0.00	7.72	0.00	0.00	0.00	0.00	0.00	0.00	16.75	0.00	19.57	17.79	0.00	48.00	0.00	0.00	0.00
Cancer (biliary tract)	0.00	0.00	0.00	0.00	0.00	0.00	0.00	0.00	0.00	0.54	0.54	1.14	0.00	0.00	0.00	0.00	0.00	0.00	0.00	0.00	0.00	0.00	0.00	0.00	0.00	0.00	0.00

Table S20. Cumulative burden of 25 infections and immunological conditions in cancer survivors. Patients were stratified into three groups: (i) patients who did not have any cancer events during the survivorship phase, (ii) patients with cancer recurrences and (iii) patients with subsequent (new) cancers. Cumulative burden per individual is shown.

Health conditions	35 years			40 years			45 years			Strata
	Cumulative Burden	Lower CI	Upper CI	Cumulative Burden	Lower CI	Upper CI	Cumulative Burden	Lower CI	Upper CI	
Autoimmune liver disease	0.01	0.01	0.01	0.01	0.01	0.01	0.01	0.01	0.01	No recurrence or subsequent cancer
Bacterial infections	0.30	0.26	0.33	0.36	0.32	0.38	0.47	0.38	0.55	No recurrence or subsequent cancer
Chronic viral hepatitis	0.04	0.04	0.05	0.04	0.04	0.05	0.05	0.04	0.05	No recurrence or subsequent cancer
Ear and upper respiratory tract infections	0.06	0.05	0.07	0.08	0.07	0.09	0.09	0.08	0.11	No recurrence or subsequent cancer
Encephalitis	0.01	0.01	0.01	0.01	0.01	0.02	0.01	0.01	0.02	No recurrence or subsequent cancer
Eye infections	0.02	0.01	0.02	0.02	0.02	0.02	0.02	0.02	0.02	No recurrence or subsequent cancer
HIV	0.04	0.03	0.05	0.05	0.04	0.07	0.05	0.04	0.07	No recurrence or subsequent cancer
Immunodeficiencies	0.00	0.00	0.01	0.00	0.00	0.01	0.00	0.00	0.01	No recurrence or subsequent cancer
Infection of anal and rectal regions	0.01	0.01	0.02	0.01	0.01	0.02	0.01	0.01	0.02	No recurrence or subsequent cancer
Infection of bones and joints	0.00	0.00	0.00	0.00	0.00	0.00	0.04	0.00	0.10	No recurrence or subsequent cancer
Infection of liver	0.01	0.01	0.01	0.01	0.01	0.01	0.01	0.01	0.02	No recurrence or subsequent cancer
Infection of skin and subcutaneous tissues	0.07	0.06	0.08	0.08	0.07	0.10	0.09	0.07	0.10	No recurrence or subsequent cancer
Infections of other or unspecified organs	0.10	0.09	0.10	0.12	0.11	0.13	0.14	0.11	0.16	No recurrence or subsequent cancer
Infections of the digestive system	0.08	0.06	0.09	0.10	0.08	0.10	0.13	0.08	0.14	No recurrence or subsequent cancer
Infections of the heart	0.03	0.02	0.04	0.03	0.02	0.04	0.03	0.02	0.05	No recurrence or subsequent cancer
Lower respiratory tract infections	0.07	0.06	0.08	0.09	0.08	0.10	0.10	0.08	0.11	No recurrence or subsequent cancer
Lupus erythematosus (local and systemic)	0.00	0.00	0.00	0.00	0.00	0.01	0.00	0.00	0.01	No recurrence or subsequent cancer
Meningitis	0.02	0.02	0.02	0.02	0.02	0.02	0.02	0.02	0.03	No recurrence or subsequent cancer
Mycoses	0.01	0.01	0.01	0.01	0.01	0.01	0.01	0.01	0.01	No recurrence or subsequent cancer
Non-acute cystitis	0.00	0.00	0.01	0.00	0.00	0.01	0.01	0.01	0.01	No recurrence or subsequent cancer
Peritonitis	0.08	0.05	0.07	0.10	0.07	0.09	0.13	0.10	0.13	No recurrence or subsequent cancer
Septicaemia	0.03	0.02	0.04	0.04	0.02	0.04	0.04	0.03	0.05	No recurrence or subsequent cancer
Systemic sclerosis	0.00	0.00	0.00	0.00	0.00	0.00	0.00	0.00	0.00	No recurrence or subsequent cancer
Urinary tract infections	0.06	0.05	0.07	0.07	0.05	0.08	0.11	0.07	0.14	No recurrence or subsequent cancer
Viral diseases (excluding chronic hepatitis/HIV)	0.05	0.04	0.06	0.06	0.05	0.07	0.06	0.05	0.07	No recurrence or subsequent cancer
Autoimmune liver disease	0.00	0.00	0.00	0.00	0.00	0.00	0.00	0.00	0.00	With cancer recurrence
Bacterial infections	0.74	0.70	0.83	0.86	0.82	0.94	0.97	0.90	1.15	With cancer recurrence
Chronic viral hepatitis	0.06	0.05	0.09	0.06	0.05	0.09	0.08	0.07	0.10	With cancer recurrence
Ear and upper respiratory tract infections	0.33	0.17	0.49	0.37	0.19	0.53	0.37	0.19	0.53	With cancer recurrence
Encephalitis	0.11	0.06	0.12	0.11	0.06	0.12	0.11	0.06	0.12	With cancer recurrence
Eye infections	0.09	0.06	0.11	0.09	0.06	0.11	0.09	0.06	0.11	With cancer recurrence
HIV	0.12	0.09	0.20	0.13	0.09	0.21	0.14	0.11	0.23	With cancer recurrence
Immunodeficiencies	0.00	0.00	0.01	0.01	0.00	0.02	0.01	0.00	0.02	With cancer recurrence
Infection of anal and rectal regions	0.01	0.01	0.01	0.01	0.01	0.01	0.01	0.01	0.01	With cancer recurrence
Infection of bones and joints	0.01	0.00	0.01	0.01	0.00	0.01	0.01	0.00	0.01	With cancer recurrence
Infection of liver	0.07	0.03	0.12	0.08	0.03	0.14	0.08	0.03	0.14	With cancer recurrence
Infection of skin and subcutaneous tissues	0.26	0.21	0.32	0.26	0.21	0.32	0.26	0.21	0.32	With cancer recurrence
Infections of other or unspecified organs	0.96	0.67	1.28	1.00	0.71	1.29	1.10	0.80	1.33	With cancer recurrence
Infections of the digestive system	0.16	0.15	0.22	0.18	0.16	0.24	0.21	0.20	0.27	With cancer recurrence
Infections of the heart	0.09	0.06	0.10	0.09	0.06	0.10	0.09	0.06	0.10	With cancer recurrence
Lower respiratory tract infections	1.08	0.53	1.51	1.10	0.53	1.53	1.16	0.58	1.56	With cancer recurrence
Lupus erythematosus (local and systemic)	0.00	0.00	0.00	0.00	0.00	0.00	0.00	0.00	0.00	With cancer recurrence
Meningitis	0.18	0.13	0.20	0.18	0.13	0.20	0.18	0.13	0.20	With cancer recurrence
Mycoses	0.31	0.16	0.49	0.31	0.16	0.49	0.31	0.16	0.49	With cancer recurrence
Non-acute cystitis	0.01	0.01	0.03	0.01	0.01	0.03	0.01	0.01	0.03	With cancer recurrence
Peritonitis	0.07	0.05	0.10	0.10	0.09	0.15	0.11	0.10	0.17	With cancer recurrence
Septicaemia	0.24	0.21	0.29	0.25	0.21	0.29	0.27	0.23	0.32	With cancer recurrence
Systemic sclerosis	0.01	0.00	0.01	0.01	0.00	0.01	0.01	0.00	0.01	With cancer recurrence
Urinary tract infections	0.23	0.19	0.30	0.28	0.23	0.33	0.32	0.31	0.41	With cancer recurrence
Viral diseases (excluding chronic hepatitis/HIV)	0.41	0.31	0.54	0.43	0.32	0.56	0.46	0.38	0.58	With cancer recurrence
Autoimmune liver disease	0.02	0.01	0.02	0.02	0.01	0.02	0.03	0.01	0.04	With subsequent cancer
Bacterial infections	2.20	1.83	2.74	2.55	2.16	3.21	2.71	2.31	3.41	With subsequent cancer
Chronic viral hepatitis	0.05	0.03	0.06	0.06	0.03	0.07	0.06	0.03	0.07	With subsequent cancer
Ear and upper respiratory tract infections	0.17	0.10	0.18	0.19	0.13	0.22	0.24	0.16	0.30	With subsequent cancer
Encephalitis	0.09	0.05	0.17	0.10	0.06	0.18	0.10	0.06	0.18	With subsequent cancer
Eye infections	0.09	0.05	0.17	0.10	0.06	0.18	0.10	0.06	0.18	With subsequent cancer
HIV	0.12	0.05	0.19	0.14	0.05	0.20	0.14	0.05	0.20	With subsequent cancer
Immunodeficiencies	0.02	0.01	0.03	0.02	0.01	0.03	0.02	0.01	0.03	With subsequent cancer
Infection of anal and rectal regions	0.16	0.01	0.28	0.16	0.01	0.28	0.16	0.01	0.28	With subsequent cancer
Infection of bones and joints	0.23	0.07	0.27	0.24	0.08	0.28	0.31	0.11	0.39	With subsequent cancer
Infection of liver	0.11	0.03	0.12	0.15	0.08	0.19	0.15	0.08	0.19	With subsequent cancer
Infection of skin and subcutaneous tissues	0.71	0.50	0.95	0.82	0.61	0.99	0.82	0.61	0.99	With subsequent cancer
Infections of other or unspecified organs	1.32	0.83	1.95	1.48	1.04	2.05	1.48	1.04	2.05	With subsequent cancer
Infections of the digestive system	1.08	0.99	2.38	1.18	1.10	2.47	1.29	1.15	2.50	With subsequent cancer
Infections of the heart	0.24	0.17	0.34	0.27	0.20	0.36	0.27	0.20	0.36	With subsequent cancer
Lower respiratory tract infections	0.79	0.71	0.87	0.97	0.80	1.18	1.04	0.84	1.19	With subsequent cancer
Lupus erythematosus (local and systemic)	0.00	0.00	0.01	0.00	0.00	0.01	0.00	0.00	0.01	With subsequent cancer
Meningitis	0.31	0.21	0.41	0.36	0.23	0.43	0.36	0.23	0.43	With subsequent cancer
Mycoses	0.27	0.18	0.40	0.31	0.21	0.42	0.31	0.21	0.42	With subsequent cancer
Non-acute cystitis	0.02	0.00	0.05	0.02	0.00	0.05	0.02	0.00	0.05	With subsequent cancer
Peritonitis	0.08	0.05	0.09	0.13	0.09	0.14	0.17	0.10	0.17	With subsequent cancer
Septicaemia	0.58	0.39	0.79	0.70	0.49	0.88	0.70	0.49	0.88	With subsequent cancer
Systemic sclerosis	0.00	0.00	0.00	0.00	0.00	0.00	0.01	0.00	0.02	With subsequent cancer
Urinary tract infections	0.61	0.36	0.71	0.72	0.45	0.81	0.72	0.45	0.81	With subsequent cancer
Viral diseases (excluding chronic hepatitis/HIV)	0.45	0.29	0.53	0.53	0.34	0.67	0.53	0.34	0.67	With subsequent cancer

Table S21. Excess years of life lost (YLL) attributable to health conditions (grouped by organ systems) among cancer survivors. Excess YLL denotes the difference in years of life lost between two groups: survivors who developed a health condition compared with survivors who did not develop a health condition. Excess YLL was estimated based on the specific age of onset of the health condition.

Health conditions by organ system	Excess YLL	Lower CI	Upper CI	Age at disease onset
Cardiovascular	10.13	7.13	14.30	32.5
Cardiovascular	5.94	1.64	10.02	35.0
Cardiovascular	4.02	0.14	6.47	37.5
Cardiovascular	3.76	0.24	5.82	40.0
Cardiovascular	3.19	0.68	5.83	42.5
Cardiovascular	-0.29	-0.29	-0.29	45.0
Endocrine	3.36	1.25	8.47	32.5
Endocrine	3.08	1.46	5.85	35.0
Endocrine	2.51	1.38	7.98	37.5
Endocrine	2.04	0.09	4.87	40.0
Endocrine	1.72	-0.37	4.76	42.5
Endocrine	1.22	0.00	2.94	45.0
Gastrointestinal	2.57	0.64	5.20	32.5
Gastrointestinal	2.15	0.67	4.26	35.0
Gastrointestinal	-0.03	-0.65	1.61	37.5
Gastrointestinal	-0.12	-1.03	0.11	40.0
Gastrointestinal	0.20	-0.71	1.88	42.5
Gastrointestinal	-0.30	-0.30	-0.30	45.0
Haematological	19.93	15.33	27.34	32.5
Haematological	15.41	8.82	23.88	35.0
Haematological	10.02	7.20	17.74	37.5
Haematological	7.62	3.89	19.88	40.0
Haematological	5.82	-0.41	8.08	42.5
Haematological	-0.26	-0.26	-0.26	45.0
Immunology and infection	6.72	5.14	10.90	32.5
Immunology and infection	4.62	3.15	6.63	35.0
Immunology and infection	3.63	1.06	5.44	37.5
Immunology and infection	3.00	1.01	6.30	40.0
Immunology and infection	2.88	2.08	4.99	42.5
Immunology and infection	0.94	0.00	1.98	45.0
Mental health	2.22	0.28	3.72	32.5
Mental health	2.33	-0.74	6.50	35.0
Mental health	2.03	1.23	5.00	37.5
Mental health	2.11	0.33	4.53	40.0
Mental health	2.46	0.82	5.50	42.5
Mental health	1.04	0.00	1.97	45.0
Musculoskeletal, Ocular & Otorhinolaryngological	3.33	1.61	4.01	32.5
Musculoskeletal, Ocular & Otorhinolaryngological	3.08	0.52	7.64	35.0
Musculoskeletal, Ocular & Otorhinolaryngological	1.97	-0.24	3.68	37.5
Musculoskeletal, Ocular & Otorhinolaryngological	1.98	-0.18	3.68	40.0
Musculoskeletal, Ocular & Otorhinolaryngological	2.31	1.12	4.99	42.5
Musculoskeletal, Ocular & Otorhinolaryngological	1.04	0.00	2.08	45.0
Neoplasm	11.67	9.29	15.27	32.5
Neoplasm	8.87	7.11	10.99	35.0
Neoplasm	5.03	3.39	8.02	37.5
Neoplasm	4.01	2.60	5.75	40.0
Neoplasm	3.38	1.72	6.41	42.5
Neoplasm	1.06	0.00	2.98	45.0
Neurological	10.98	7.27	13.34	32.5
Neurological	8.65	4.04	15.96	35.0
Neurological	6.45	3.28	11.02	37.5
Neurological	5.95	3.26	8.42	40.0
Neurological	4.34	0.87	9.81	42.5
Neurological	1.48	0.28	4.76	45.0
Pulmonary	8.07	6.07	11.65	32.5
Pulmonary	5.51	2.50	9.63	35.0
Pulmonary	3.98	0.85	7.42	37.5
Pulmonary	3.40	0.64	8.64	40.0
Pulmonary	3.09	0.03	5.81	42.5
Pulmonary	1.28	0.00	2.30	45.0
Renal	12.50	8.46	16.33	32.5
Renal	7.93	3.54	8.42	35.0
Renal	4.40	1.25	9.00	37.5
Renal	4.08	0.86	6.62	40.0
Renal	4.38	1.72	12.02	42.5
Renal	1.74	0.00	2.51	45.0
Reproductive	0.42	-1.83	2.29	32.5
Reproductive	0.32	-2.06	2.51	35.0
Reproductive	1.30	-0.11	4.93	37.5
Reproductive	1.14	-0.68	3.61	40.0
Reproductive	0.78	-0.58	3.49	42.5
Reproductive	1.36	1.11	3.29	45.0
